# Supplementary material for: A potent small-molecule inhibitor of the DCN1-UBC12 interaction that selectively blocks cullin 3 neddylation
Source: Nat Commun. 2017 Oct 27;8:1150. doi: 10.1038/s41467-017-01243-7 (PMC5658359; doi:10.1038/s41467-017-01243-7)
Supplement: Supplementary file 1 — Supplementary Information [file 41467_2017_1243_MOESM1_ESM.pdf]

Supplementary Table 1. Summary of binding affinities between the designed lead compounds and indicated DCN recombinant proteins.

	**DCN1** (FP, K_i, nM)	**DCN2** (FP, K_i, nM)	**DCN3 (BLI)** (BLI, K_d, nM)	**DCN4** (BLI, K_d, nM)	**DCN5** (BLI, K_d, nM)
**DI-591**	12.4 ± 1.2	10.4 ± 2.1	> 10000	> 10000	> 10000
**DI-591DD**	6403 ± 213	4631 ± 455	> 10000	> 10000	> 10000
**46**	21.9±2.3	10.6 ± 0.9	> 10000	> 10000	> 10000

Supplementary Table 2. Crystallography Data Collection and Refinement Statistics.

**Data Collection**	**DCN1-591**
PDBID	**5UFI**
SpaceGroup	$P2_12_12_1$
Unit Cell (Å)	a = 62.142 b= 101.647 c = 173.521
	$\alpha = \beta = \gamma = 90°$
Wavelength (Å)	0.9787
Resolution (Å)[1]	2.58 (2.58-2.62)
Rsym[2]	0.084 (0.616)
<I/σI>[3]	10 (2)
Completeness (%)[4]	99.9 (99.7)
Redundancy	5.3 (5.2)
**Refinement**	
Resolution (Å)	2.58
R-Factor [5]	0.2246
Rfree [6]	0.2606
Protein atoms	6185
Ligands	4
Water Molecules	15
Unique Reflections	35332
R.m.s.d.[7]	
Bonds	0.01
Angles	1.1
MolProbity Score[8]	1.43
Clash Score[8]	2.02

[1]Statistics for highest resolution bin of reflections in parentheses.

[2]$R_{sym} = \Sigma_h\Sigma_j \mid I_{hj} - \langle I_h \rangle \mid / \Sigma_h\Sigma_j I_{hj}$, where I_{hj} is the intensity of observation j of reflection h and $\langle I_h \rangle$ is the mean intensity for multiply recorded reflections.

[3]Intensity signal-to-noise ratio.

[4]Completeness of the unique diffraction data.

[5]R-factor = $\Sigma_h \mid |F_o| - |F_c| \mid / \Sigma_h|F_o|$, where F_o and F_c are the observed and calculated structure factor amplitudes for reflection h.

[6]R_{free} is calculated against a 5% random sampling of the reflections that were removed before structure refinement.

[7]Root mean square deviation of bond lengths and bond angles.

[8]Chen et al. (2010) MolProbity: all-atom structure validation for macromolecular crystallography. Acta Crystallographica D66:12-21.

Supplementary Figure 1:

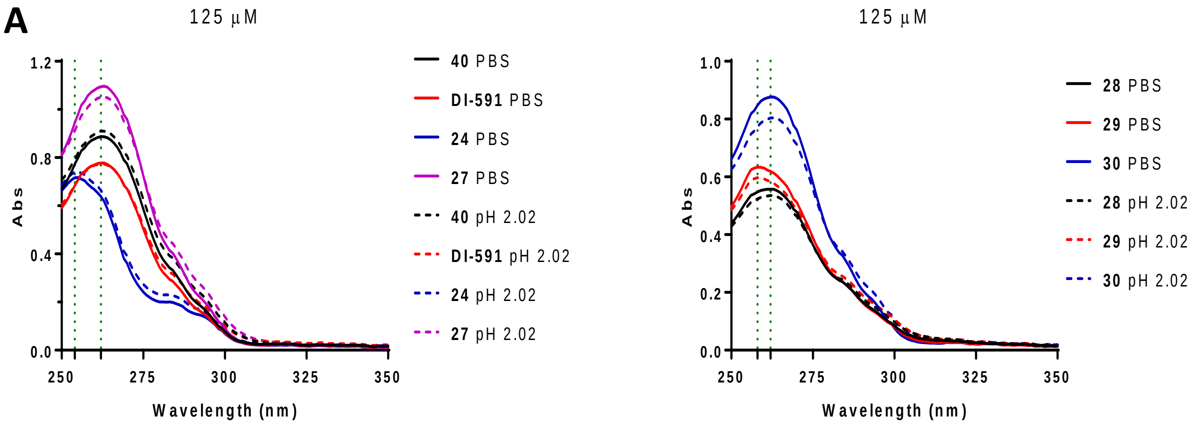

**B**	**24**	**27**	**28**	**29**	**30**	**40**	**DI-591**
**pH 2.02 (μM)**	1476±38	227±10	> 20000	1052±21	> 20000	4754±77	> 20000
**pH 7.4 (μM)**	251±5	31±2	7472±98	1279±15	> 20000	1906±43	> 20000

Supplementary Figure 1. **Solubility of representative compounds.** (**a**) UV-Vis absorption spectra of seven compounds at pH values of 2.0 and 7.4. (**b**) Solubility (μM) of seven compounds at pH values of 2.0 and 7.4.

Supplementary Figure 2:

```
sp|Q92564|DCNL4      MHSDAAAVNFQLNSHLSTLANIHKIYHTLNKLNLTEDIGQDDHQ---TGSLRS-CSSSDC     56
sp|Q9BTE7|DCNL5      ------------------------------------------------------------     0
sp|Q8IWE4|DCNL3      -----------MGQCVTKC------------KNPSSTLGSKNGDREPSNKSHSRRGAGHR     37
sp|Q96GG9|DCNL1      -----------------------------------------------MNKLKS--SQKDK     11
sp|Q6PH85|DCNL2      -----------------------------------------------MHKLKS--SQKDK     11

sp|Q92564|DCNL4      FNKVMPPRKKRRPAS-----GDDLSAKKSRHDSMYRKYDST-----RIKTEEEAFSSKRC     106
sp|Q9BTE7|DCNL5      ----MPVKKKRKSPGVAAAVAEDGGLKKCKISSYCRSQPPA-----RLISGEEHFSSKKC     51
sp|Q8IWE4|DCNL3      EEQVPPCGKPGGDILVNGT------KKAEAATEACQLPTSSGDAGRESKSNAEESSLQRL     91
sp|Q96GG9|DCNL1      VRQFMIFTQSSEKTAVSCLSQNDW-KLDVATDNFFQNPEL---YIRE--SVKGSLDRKKL     65
sp|Q6PH85|DCNL2      VRQFMACTQAGERTAIYCLTQNEW-RLDEATDSFFQNPDS---LHRE--SMRNAVDKKKL     65
                             :                       .  :          .  :     . ::

sp|Q92564|DCNL4      LEWFYEYAGT--DDVVGPEGMEKFCEDIGVEPENVVMLVLAWKLDAQNMGYFTLQEWLKG     164
sp|Q9BTE7|DCNL5      LAWFYEYAGP--DEVVGPEGMEKFCEDIGVEPENIIMLVLAWKLEAESMGFFTKEEWLKG     109
sp|Q8IWE4|DCNL3      EELFRRYKDERE-DAILEEGMERFCNDLCVDPTEFRVLLLAWKFQAATMCKFTRKEFFDG     150
sp|Q96GG9|DCNL1      EQLYNRYKDPQDENKIGIDGIQQFCDDLALDPASISVLIIAWKFRAATQCEFSKQEFMDG     125
sp|Q6PH85|DCNL2      ERLYGRYKDPQDENKIGVDGIQQFCDDLSLDPASISVLVIAWKFRAATQCEFSRKEFLDG     125
                        : .*      : :  :*:::**:*: ::* .. :*::***: * .   *: :*::.*

sp|Q92564|DCNL4      MTSLQCDTTEKLRNTLDYLRSFLNDSTNFKLIYRYAFDFAR--EKDQRSLDINTAKCMLG     222
sp|Q9BTE7|DCNL5      MTSLQCDCTEKLQNKFDFLRSQLNDISSFKNIYRYAFDFAR--DKDQRSLDIDTAKSMLA     167
sp|Q8IWE4|DCNL3      CKAISADSIDGICARFPSLLTEAKQEDKFKDLYRFTFQFGLDSEEGQRSLHREIAIALWK     210
sp|Q96GG9|DCNL1      MTELGCDSIEKLKAQIPKMEQELKEPGRFKDFYQFTFNFAK--NPGQKGLDLEMAIAYWN     183
sp|Q6PH85|DCNL2      MTELGCDSMEKLKALLPRLEQELKDTAKFKDFYQFTFTFAK--NPGQKGLDLEMAVAYWK     183
                      . : .*  : :   :  :    ::   ** :*:::* *.   :  *:.*. : * .

sp|Q92564|DCNL4      LLLGK-IWPLFPVFHQFLEQSK--YKVINKDQWCNVLEFSRTINLDLSNYDEDGAWPVLL     279
sp|Q9BTE7|DCNL5      LLLGR-TWPLFSVFYQYLEQSK--YRVMNKDQWYNVLEFSRTVHADLSNYDEDGAWPVLL     224
sp|Q8IWE4|DCNL3      LVFTQNNPPVLDQWLNFLTENPSGIKGISRDTWNMFLNFTQVIGPDLSNYSEDEAWPSLF     270
sp|Q96GG9|DCNL1      LVLNG-RFKFLDLWNKFLLEHH--KRSIPKDTWNLLLDFSTMIADDMSNYDEEGAWPVLI     240
sp|Q6PH85|DCNL2      LVLSG-RFKFLDLWNTFLMEHH--KRSIPRDTWNLLLDFGNMIADDMSNYDEEGAWPVLI     240
                     *::      .:  :  :* :     : : :* *  .*:*   :  *:***.*: *** *:

sp|Q92564|DCNL4      DEFVEWYKDKQMS--------------------- 292
sp|Q9BTE7|DCNL5      DEFVEWQKVRQTS--------------------- 237
sp|Q8IWE4|DCNL3      DTFVEWEMERRKREGEGRGALSSGPEGLCPEEQT 304
sp|Q96GG9|DCNL1      DDFVEFARPQIA--GTKSTTV------------- 259
sp|Q6PH85|DCNL2      DDFVEYARPVVT--GGKRSLF------------- 259
                     * ***:
```

Percent Identity Matrix created by Clustal2.1					
sp|Q92564|DCNL4	100	70.69	29.28	29.34	31.4
sp|Q9BTE7|DCNL5	70.69	100	29.57	29.74	30.17
sp|Q8IWE4|DCNL3	29.28	29.57	100	37.55	39.92
sp|Q96GG9|DCNL1	29.34	29.74	37.55	100	77.61
sp|Q6PH85|DCNL2	31.4	30.17	39.92	77.61	100

Supplementary Figure 2. Sequence alignment of DCN protein family. Multiple sequence alignment of DCN1-5 (also known as DCNL1-5) by ClustalW. Rectangular boxes indicate the residues forming the binding site for DI-591. Uniprot accession numbers for each protein are listed in the sequence alignment.

Supplementary Figure 3:

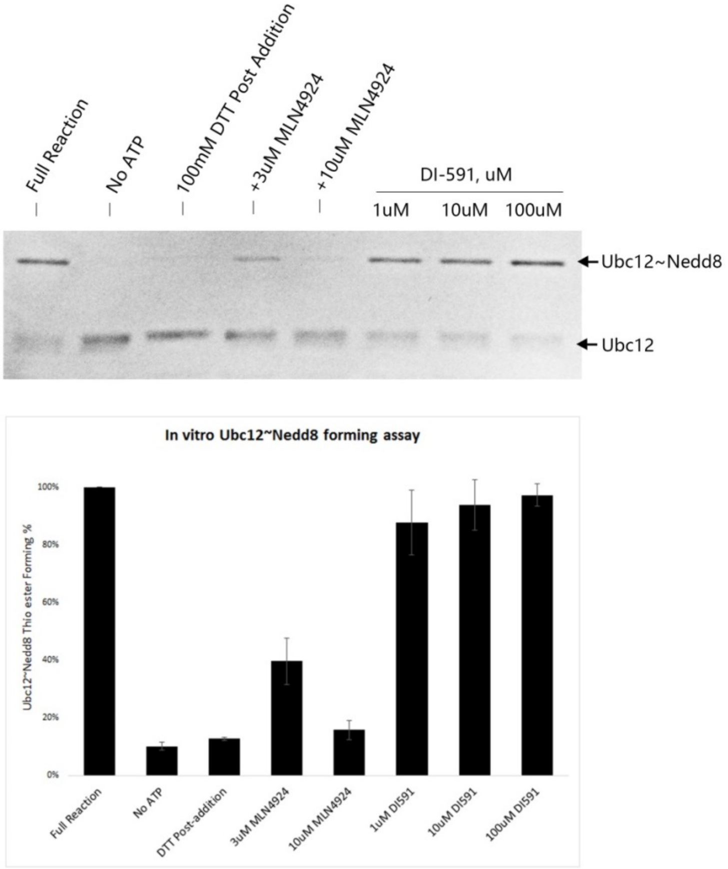

Supplementary Figure 3. In vitro UBC12~NEDD8 thio ester forming assay. NEDD8, NEDD8 E1 (APP-BP1/Uba3) and UBC12 were purchased from Boston Biochem. 10μM NEDD8, 25nM NEDD8 E1, and 2μM UBC12 were incubated in 50mM Tris-HCl (pH 7.4), 5mM $MgCl_2$, 0.5mM DTT, and 0.1mg/ml BSA, with indicated compounds at 25℃ for 10min. Reactions were initiated by addition of 2mM ATP (or dH_2O) and were run for 5min at 25℃. Reaction mixtures were then added with 1M DTT (or dH_2O) and kept on ice for 1min followed by addition of SDS Loading buffer (without DTT). The products were separated by SDS-PAGE, visualized by Coomassie stain and quantified using an ImageLab imager (Bio-Rad).

Supplementary Figure 4:

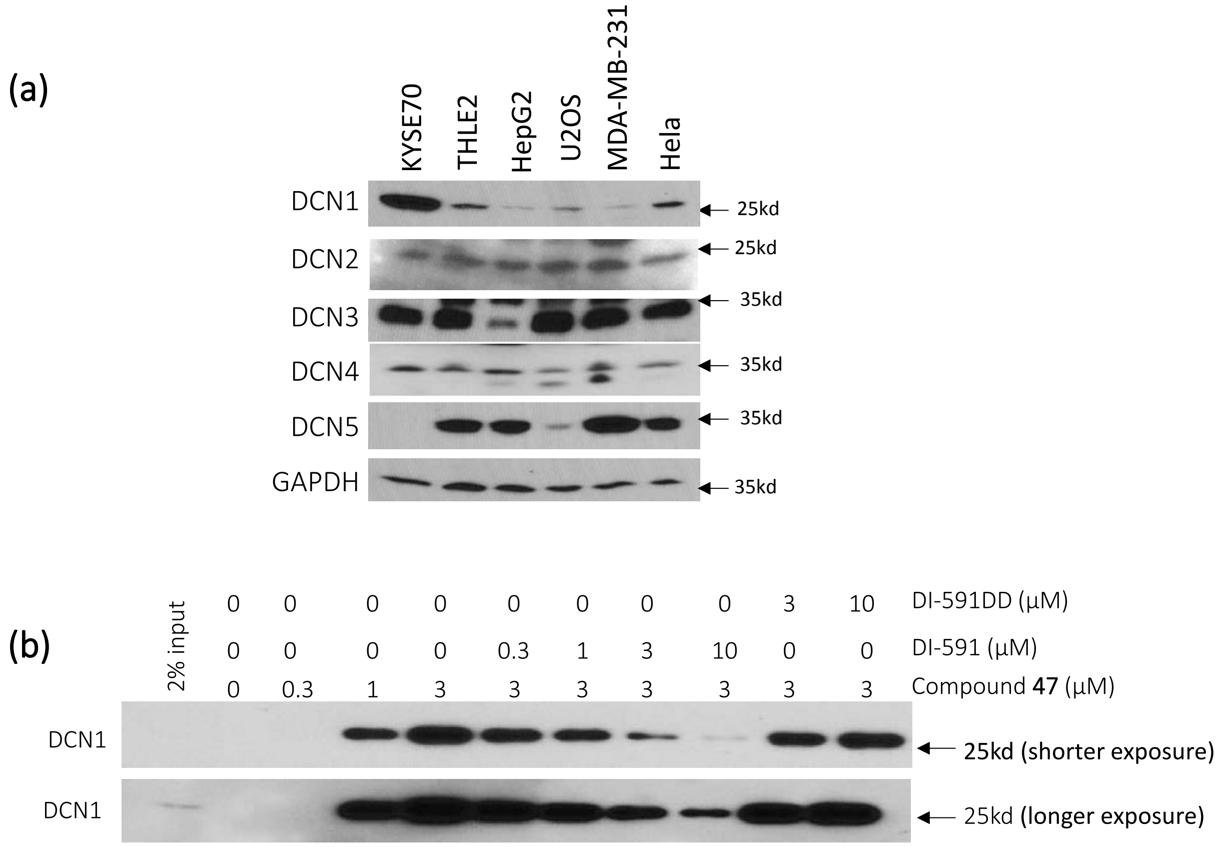

Supplementary Figure 4. (a) Esophageal cancer KYSE70, immortalized liver THLE2, hepatoma HepG2, osteosarcoma U2OS, breast cancer MDA-MB-231 and ovarian cancer Hela cells growing in exponential phase were harvested and lysed. The cell lysates were analyzed by western blotting for the expression of DCN1 (#GWB-E3D700, GenWay Biotech), DCN2 (#ARP68256_P050, Aviva Systems, San Diego, CA), DCN3 (#GWB-MS982A, GenWay Biotech) DCN4 (#PA5-28562, Thermo Fisher Scientific) and DCN5 (#PA5-62257, Thermo Fisher Scientific) proteins. GAPDH was used as a loading control. **(b)** Biotinylated compound **47** pulls down DCN1 protein from KYSE140 cell lysates in a dose-dependent manner. DI-591 but not its enantiomeric control, DI-591DD, effectively competes off the binding of **47** to DCN1 protein.

Supplementary Figure 5:

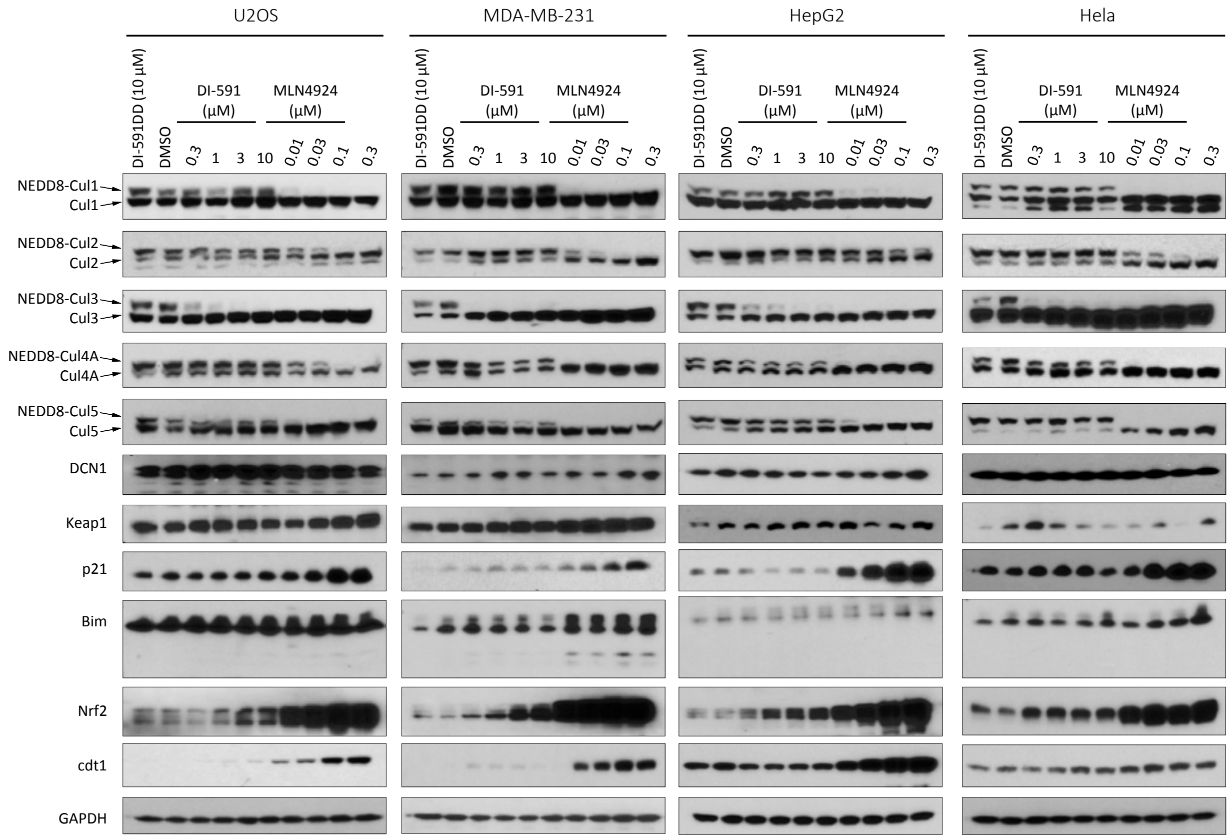

Supplementary Figure 5. U2OS, MDA-MB-231, HepG2 and Hela cell lines were treated as indicated for 24 h, the expression level and neddylation state of the cullin family of proteins and the abundance of their CRL substrates was examined by western blotting. GAPDH was used as a loading control.

Supplementary Figure 6:

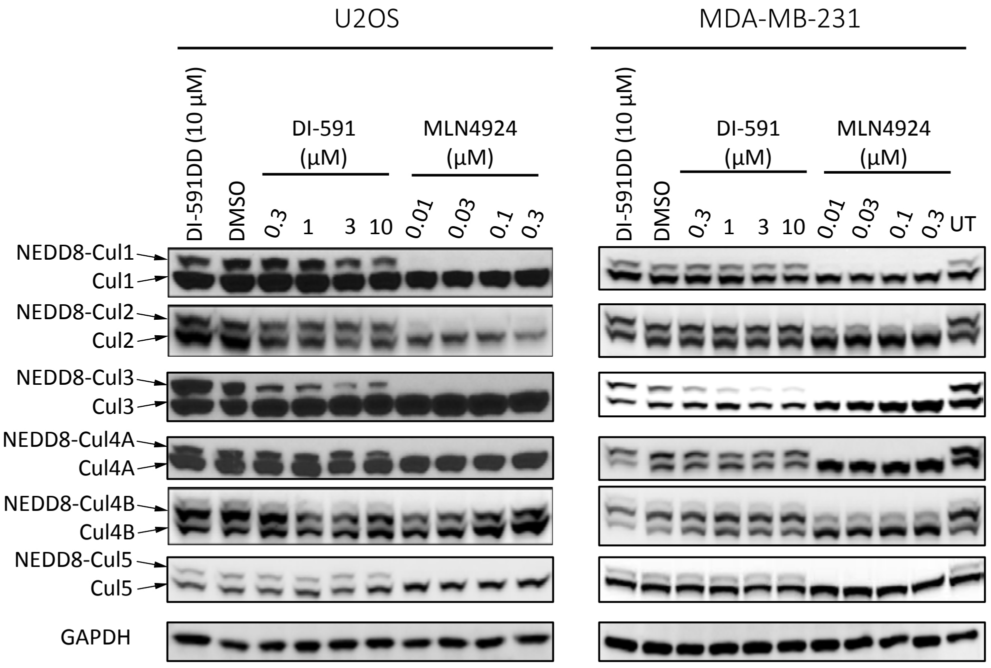

Supplementary Figure 6. The expression level and neddylation state of the cullin family of proteins were examined by western blotting in U2OS and MDA-MB-231 cells treated as indicated for 24 h. GAPDH was used as a loading control. These experiments were performed by a second scientist independently.

Supplementary Figure 7:

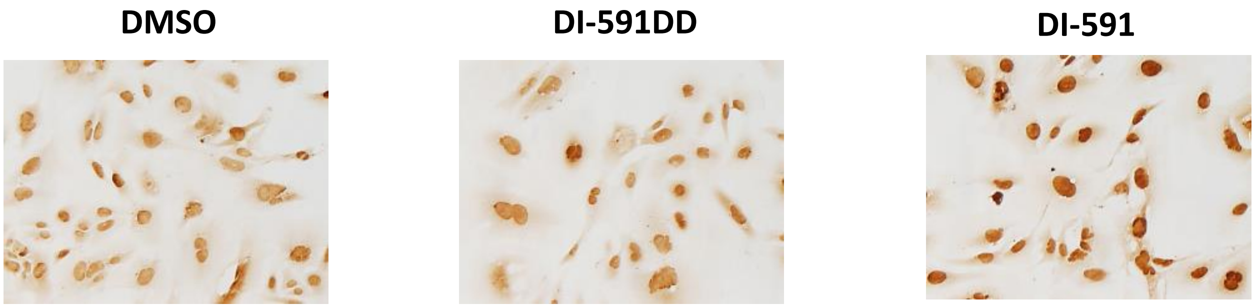

Supplementary Figure 7. Detection of NRF2 protein by IHC (immunohistochemical staining) demonstrates that DI-591 increases the levels of NRF2 in liver cells. THLE2 liver cells were grown in chamber slides and treated with DI-591 at 10 µM or DI-591DD at 10 µM for 16 h, DMSO was used as a treatment control.

Supplementary Figure 8:

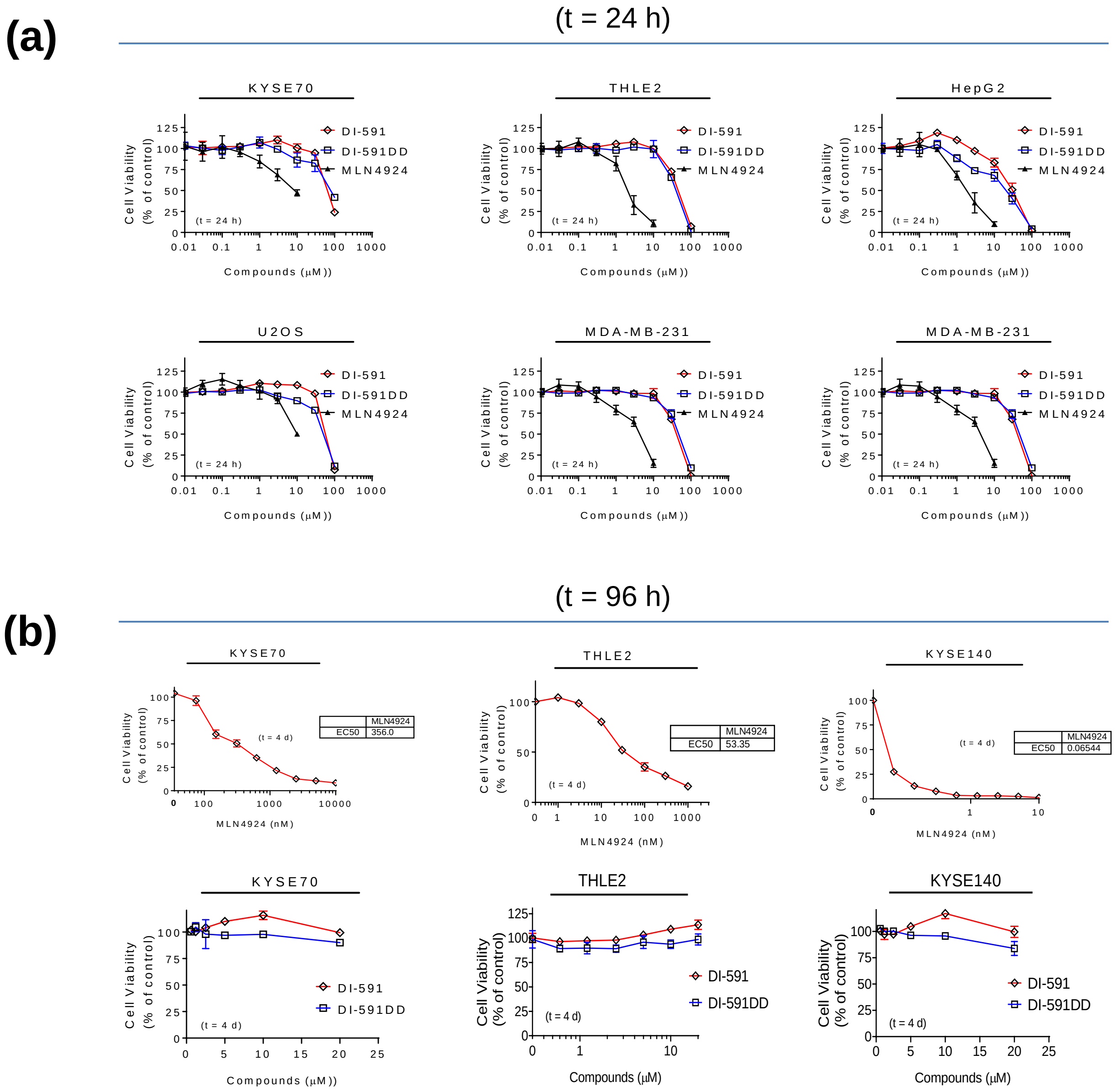

Supplementary Figure 8. **Cell growth inhibitory activity of DI-591, DI-591DD, and MLN49224 in a panel of 6 cell lines.** (a) The indicated cell lines were treated with MLN4924, DI-591 or DI-591DD for 24 h. Cell viability was determined with a WST assay. (b) The indicated cell lines were treated with MLN4924, DI-591 or DI-591DD for 4 days. Cell viability was determined with a WST assay.

Supplementary Figure 9:

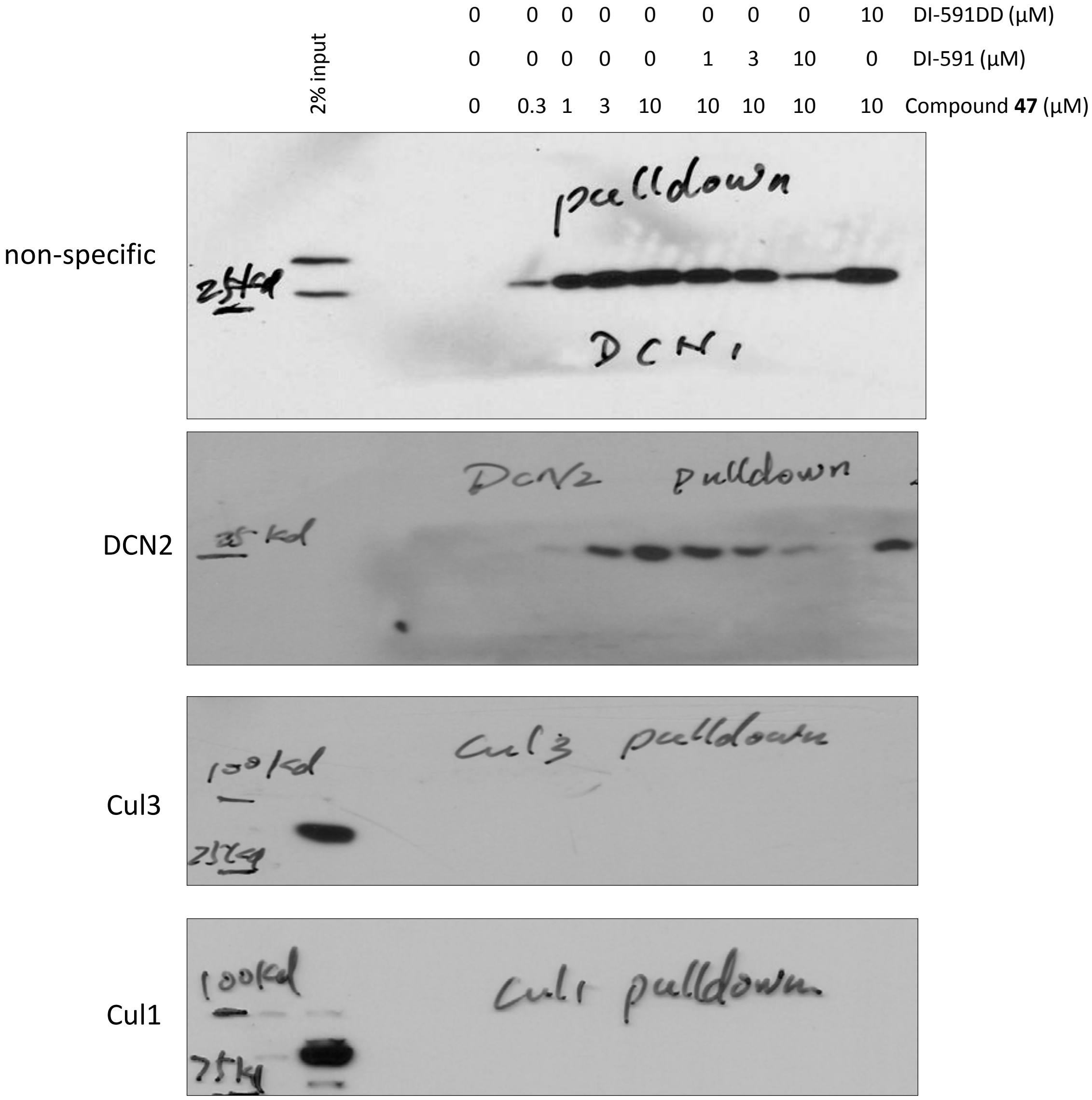

Supplementary Figure 9. Uncropped western blot images for Figure 4b.

Supplementary Figure 10:

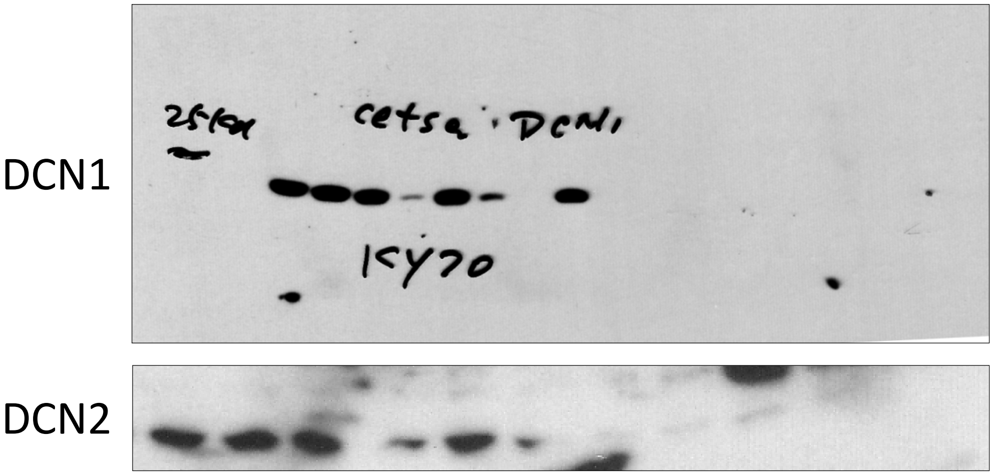

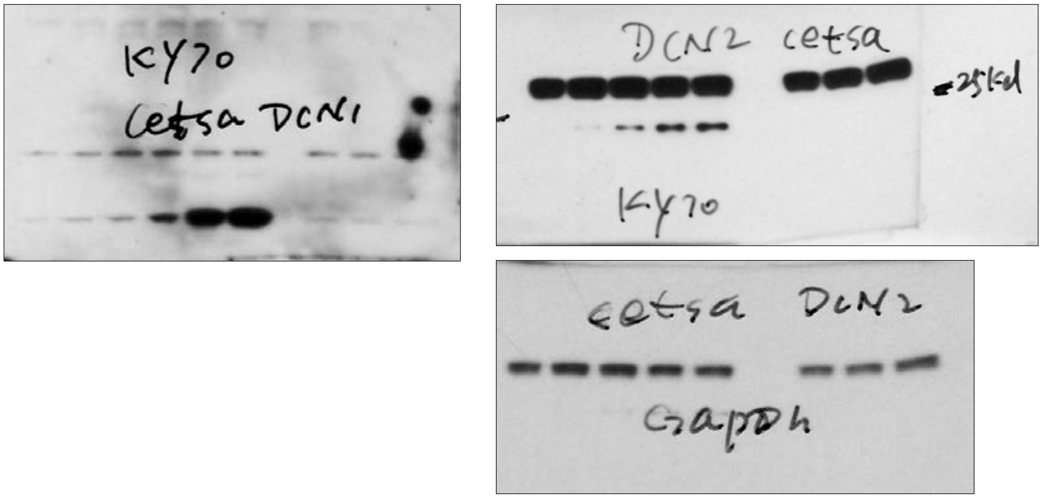

Supplementary Figure 10. Uncropped western blot images for Figures 4c, 4d and 4e,

Supplementary Figure 11:

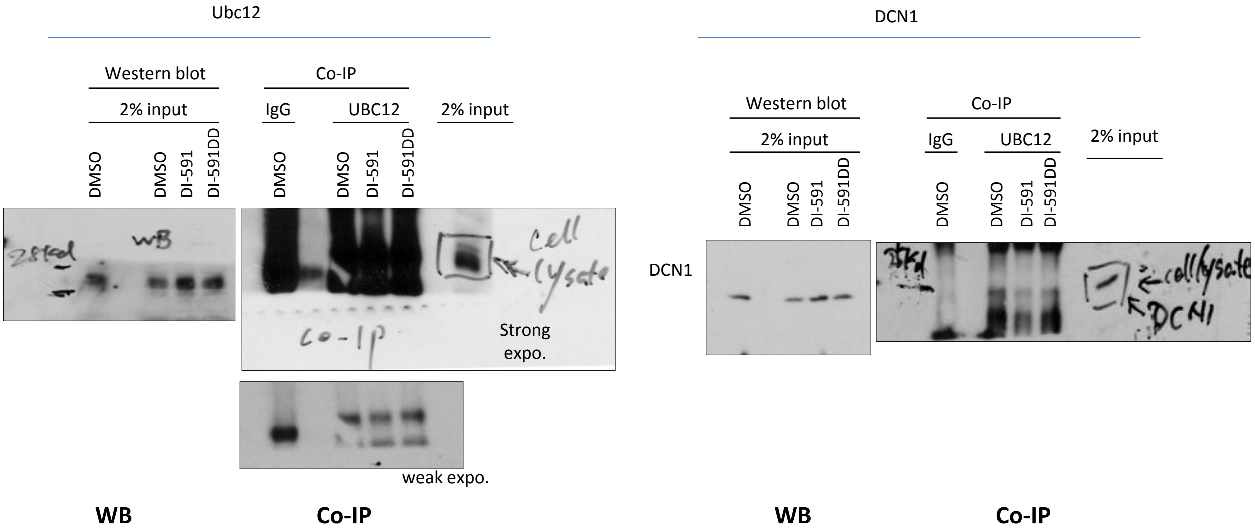

Supplementary Figure 11. Uncropped Western blot images for Figure 4f.

Supplementary Figure 12:

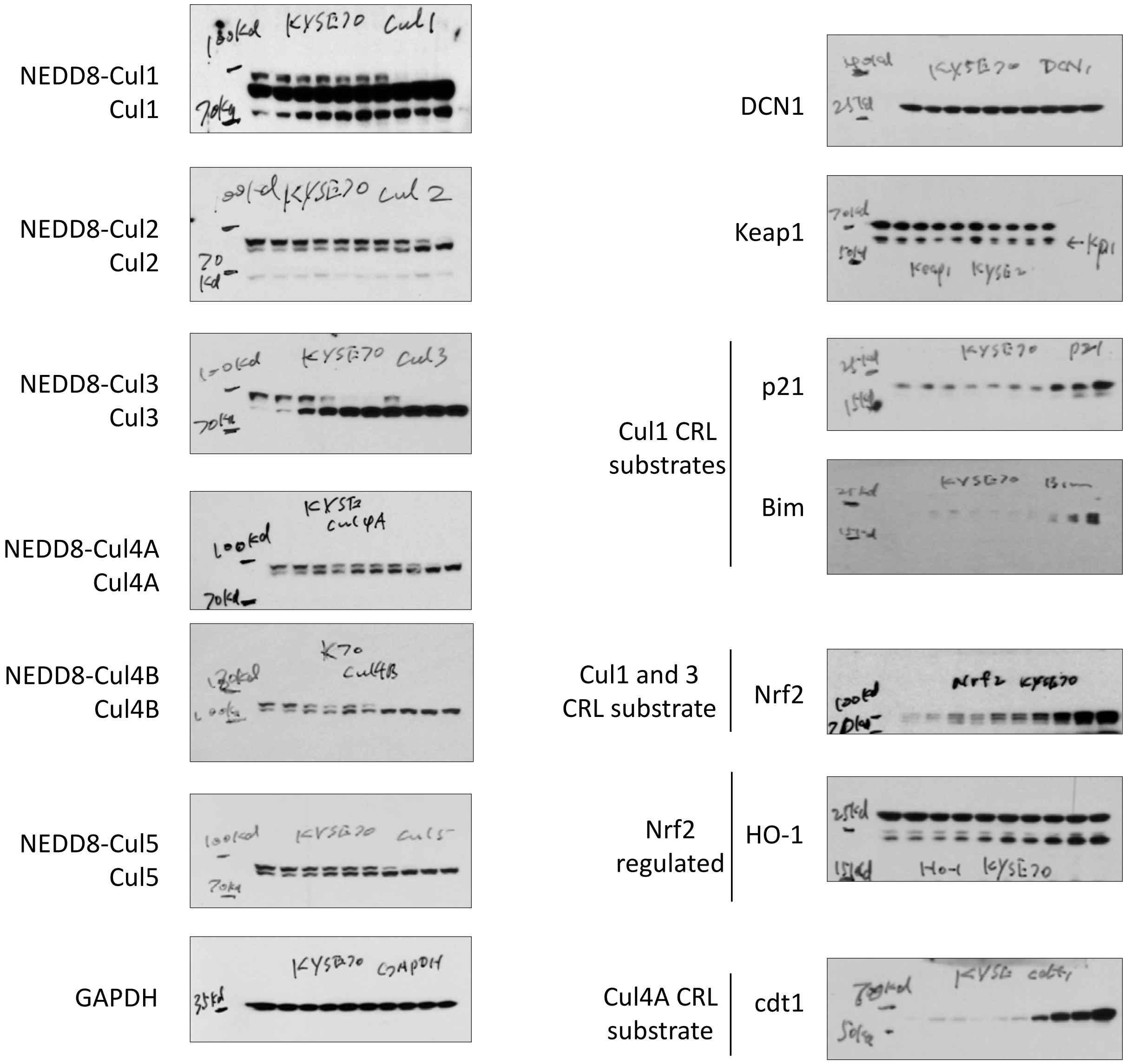

Supplementary Figure 12. Uncropped western blot images for Figure 5a (KYSE70 cell line).

Supplementary Figure 13:

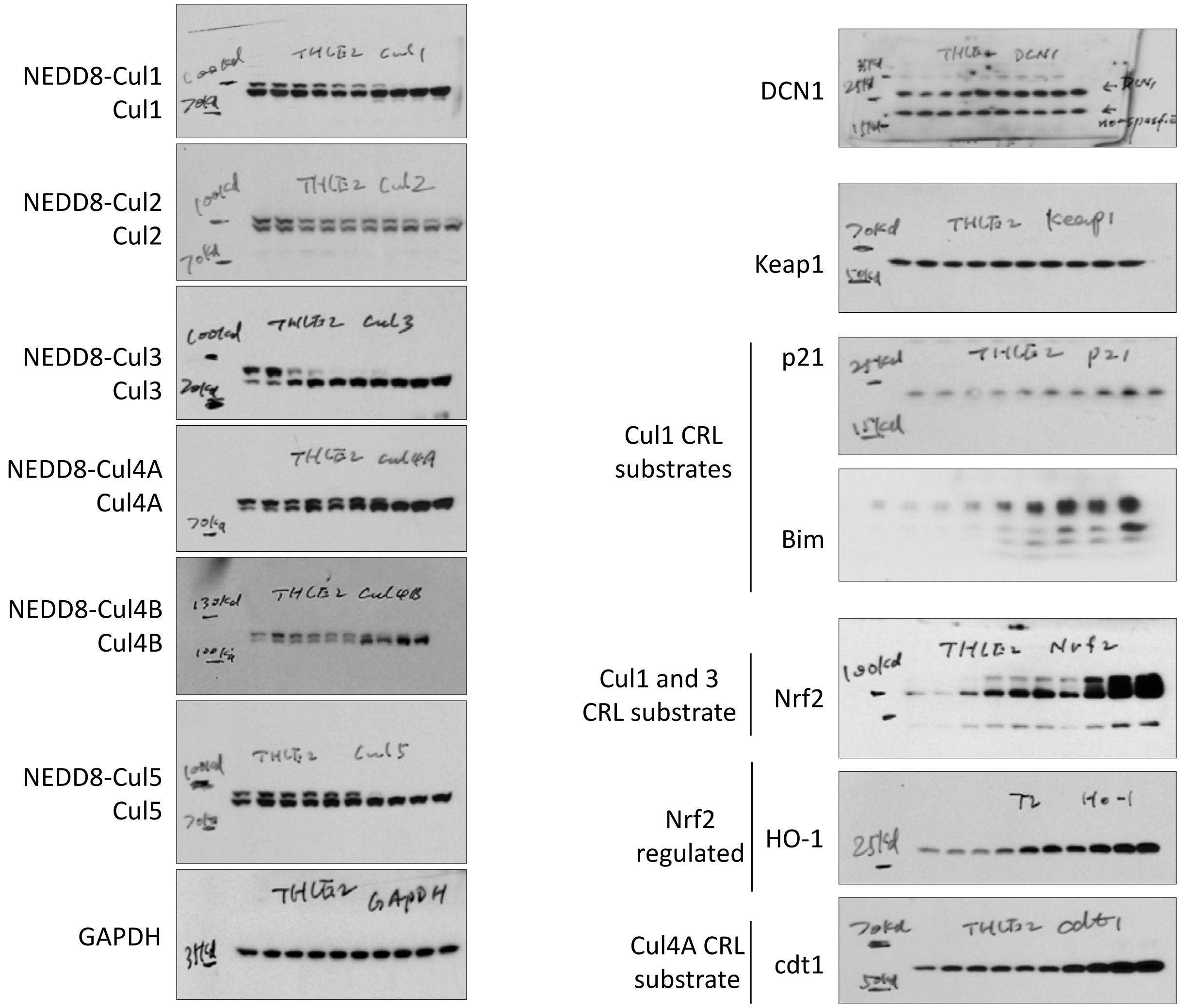

Supplementary Figure 13. Uncropped western blot images for Figure 5a (THLE2 cell line).

Supplementary Figure 14:

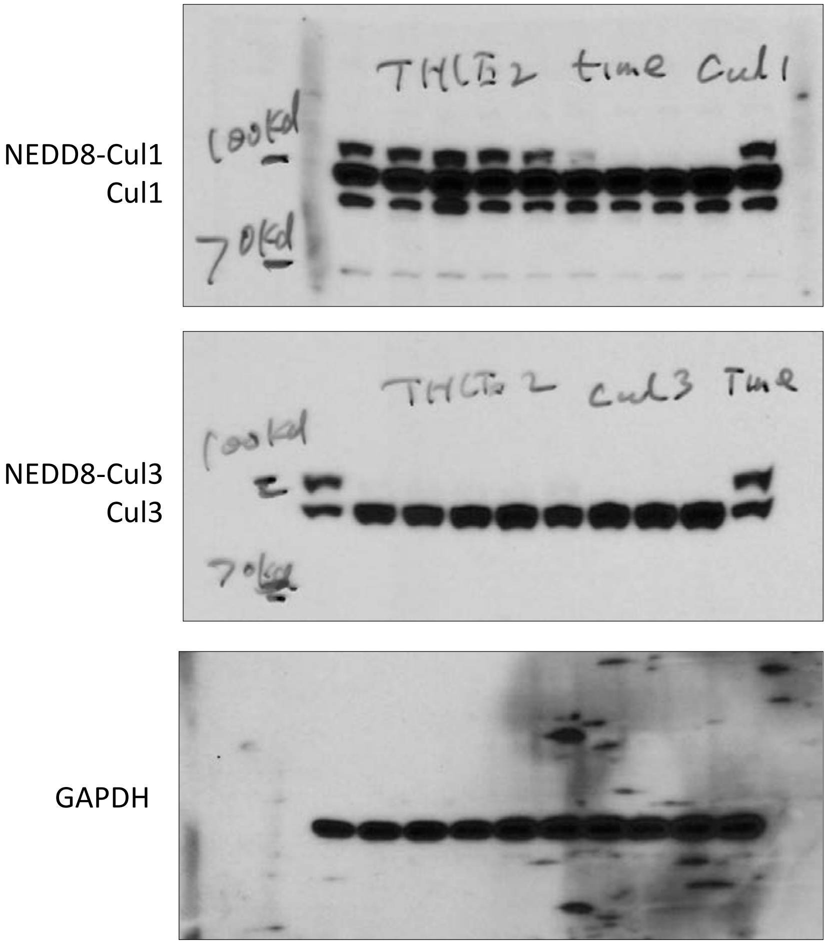

Supplementary Figure 14. Uncropped western blot images for repeat experiment for Figure 5b.

Supplementary Figure 15:

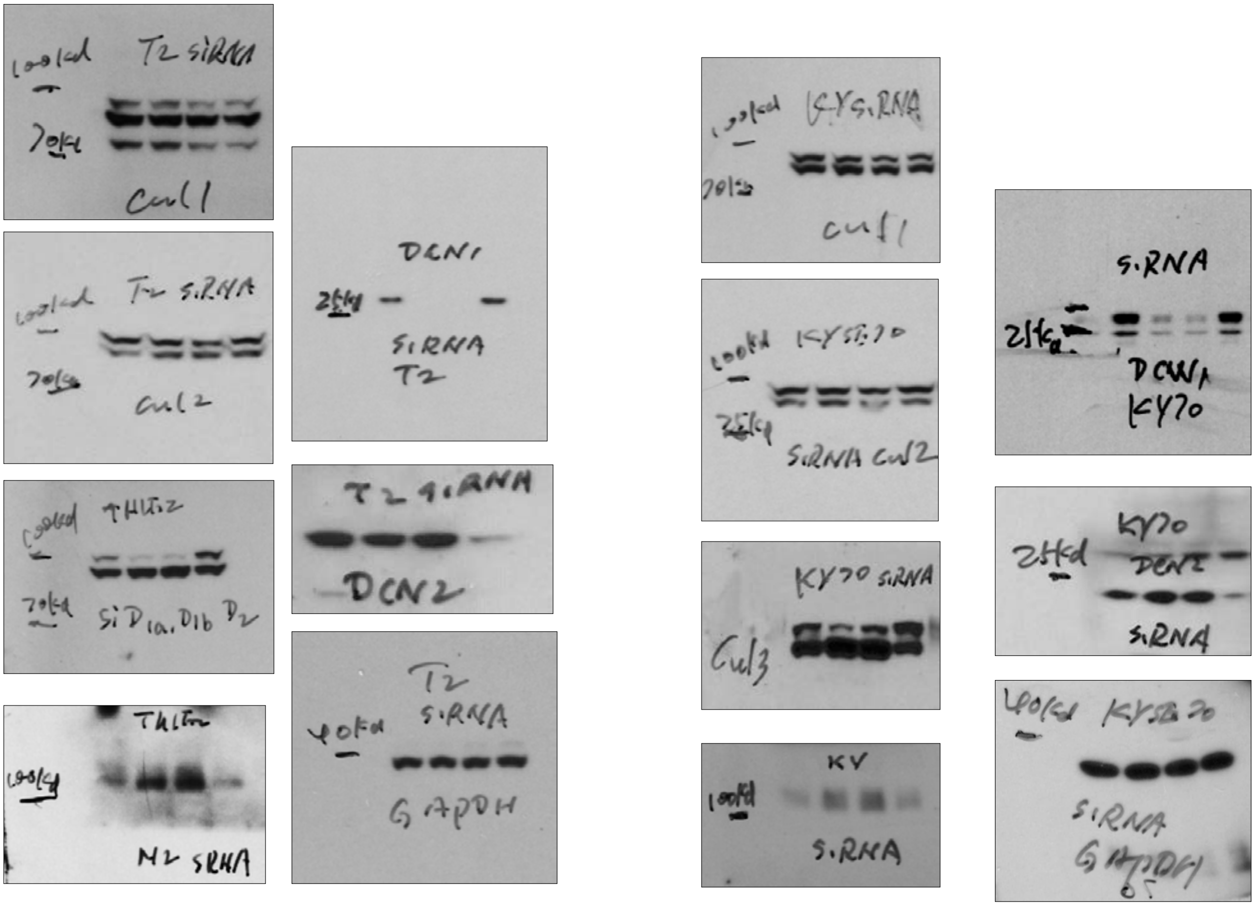

Supplementary Figure 15. Uncropped western blot images Figure 5d.

Supplementary Figure 16:

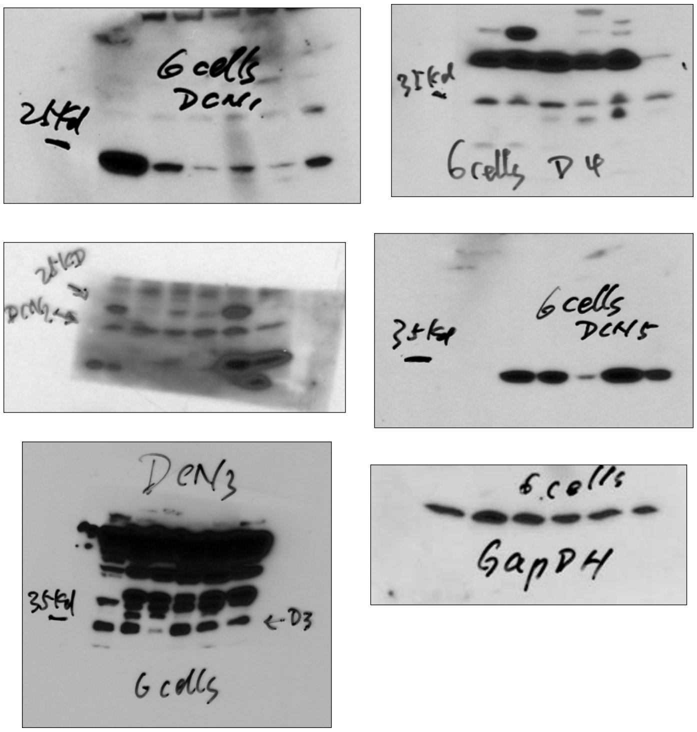

Supplementary Figure 16. Uncropped western blot images for Supplementary Figure 4a.

Supplementary Figure 17:

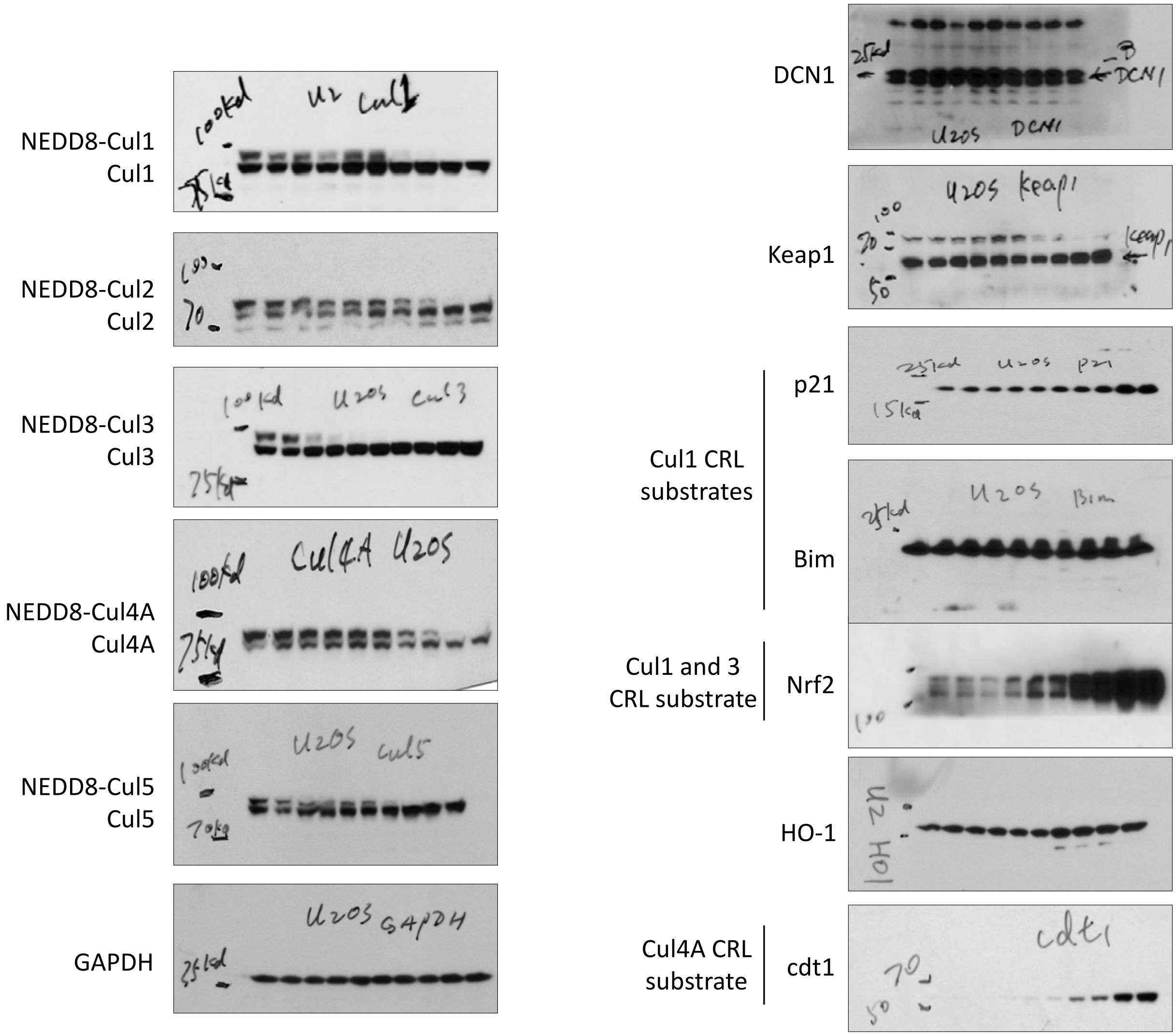

Supplementary Figure 17. Uncropped western blot images for Supplementary Figure 5 (U2OS cell line).

Supplementary Figure 18:

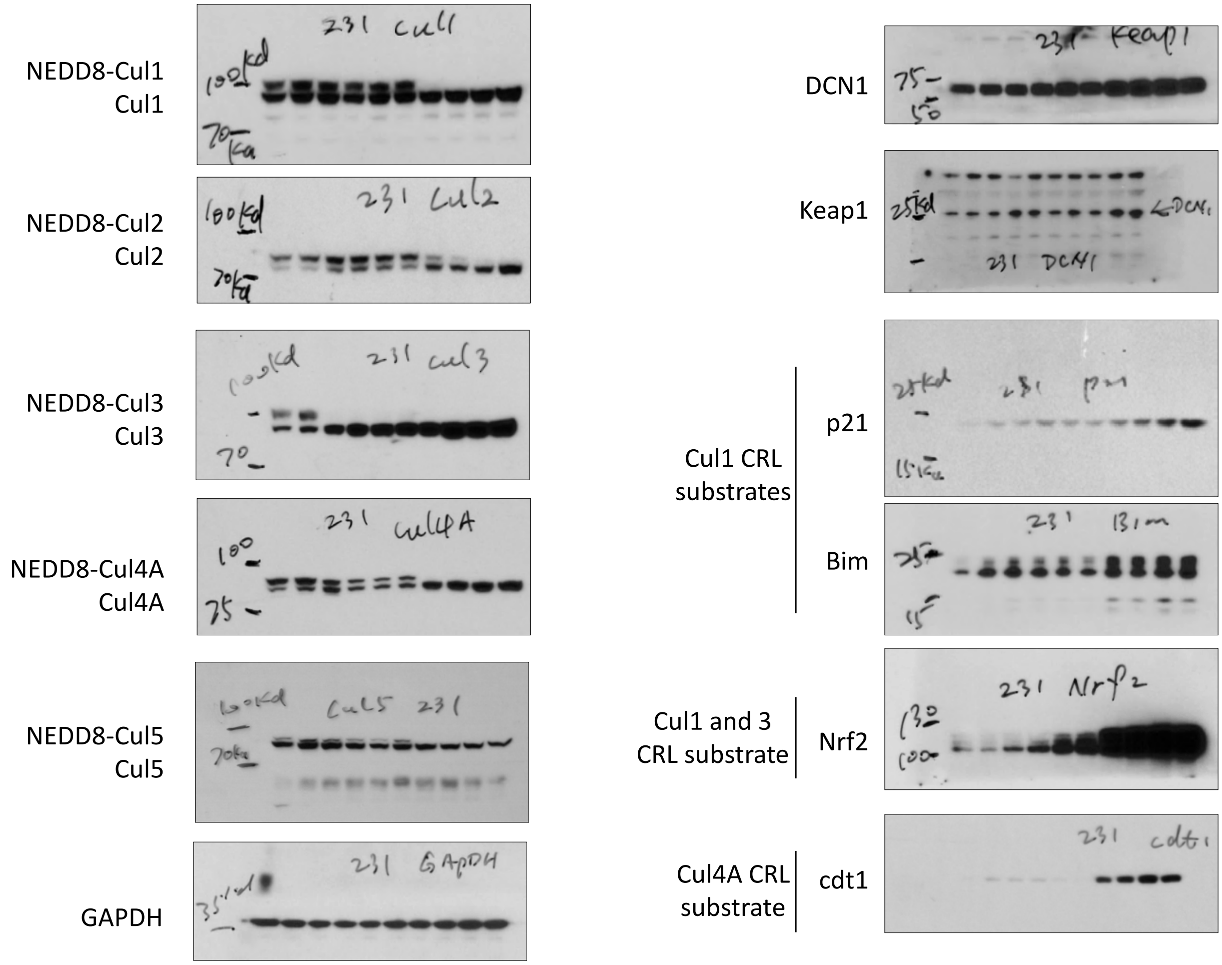

Supplementary Figure 18. Uncropped western blot images for Supplementary Figure 5 (MDA-MB-231 cell line).

Supplementary Figure 19:

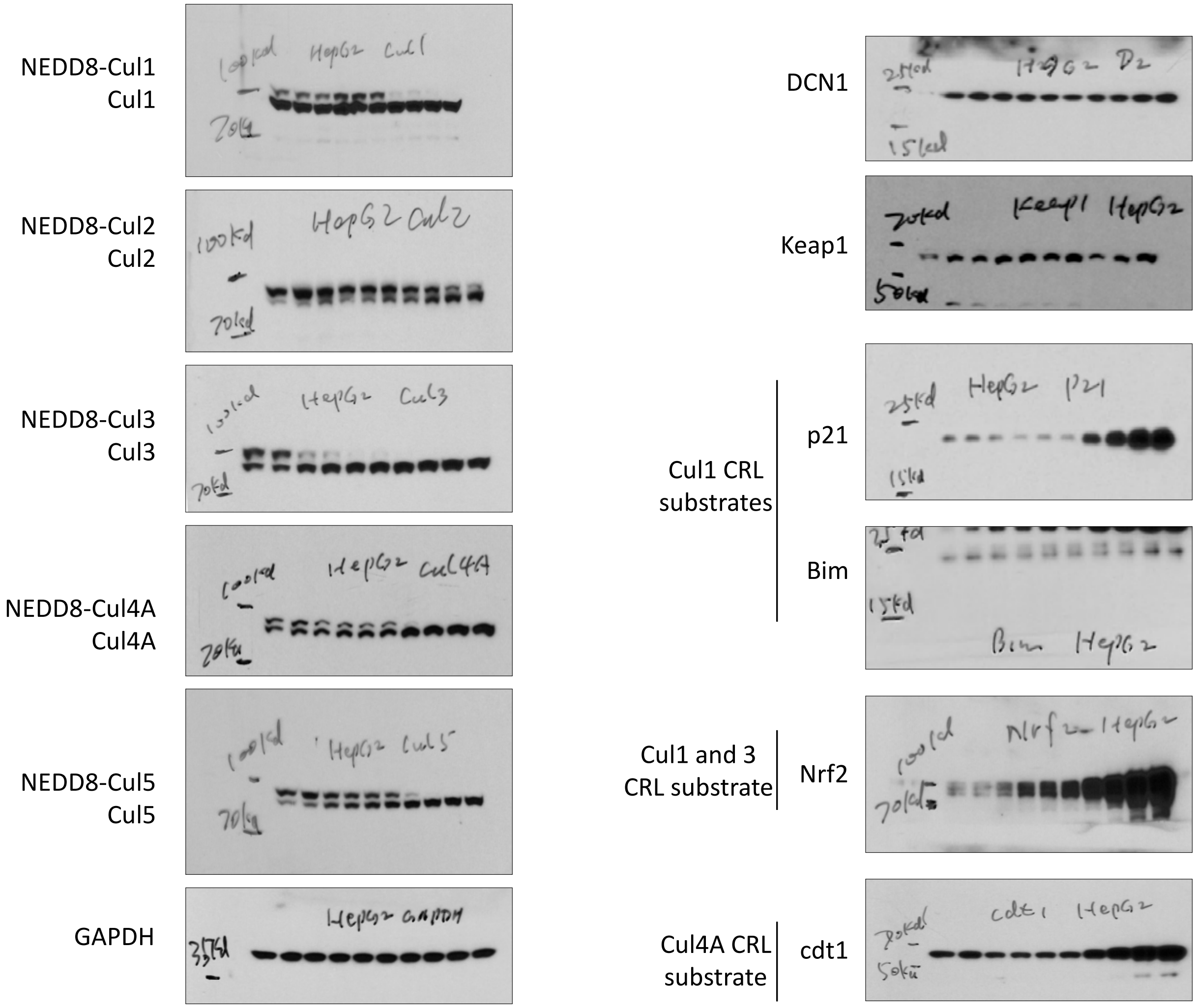

Supplementary Figure 19. Uncropped western blot images for Supplementary Figure 5 (HepG2 cell line).

Supplementary Figure 20:

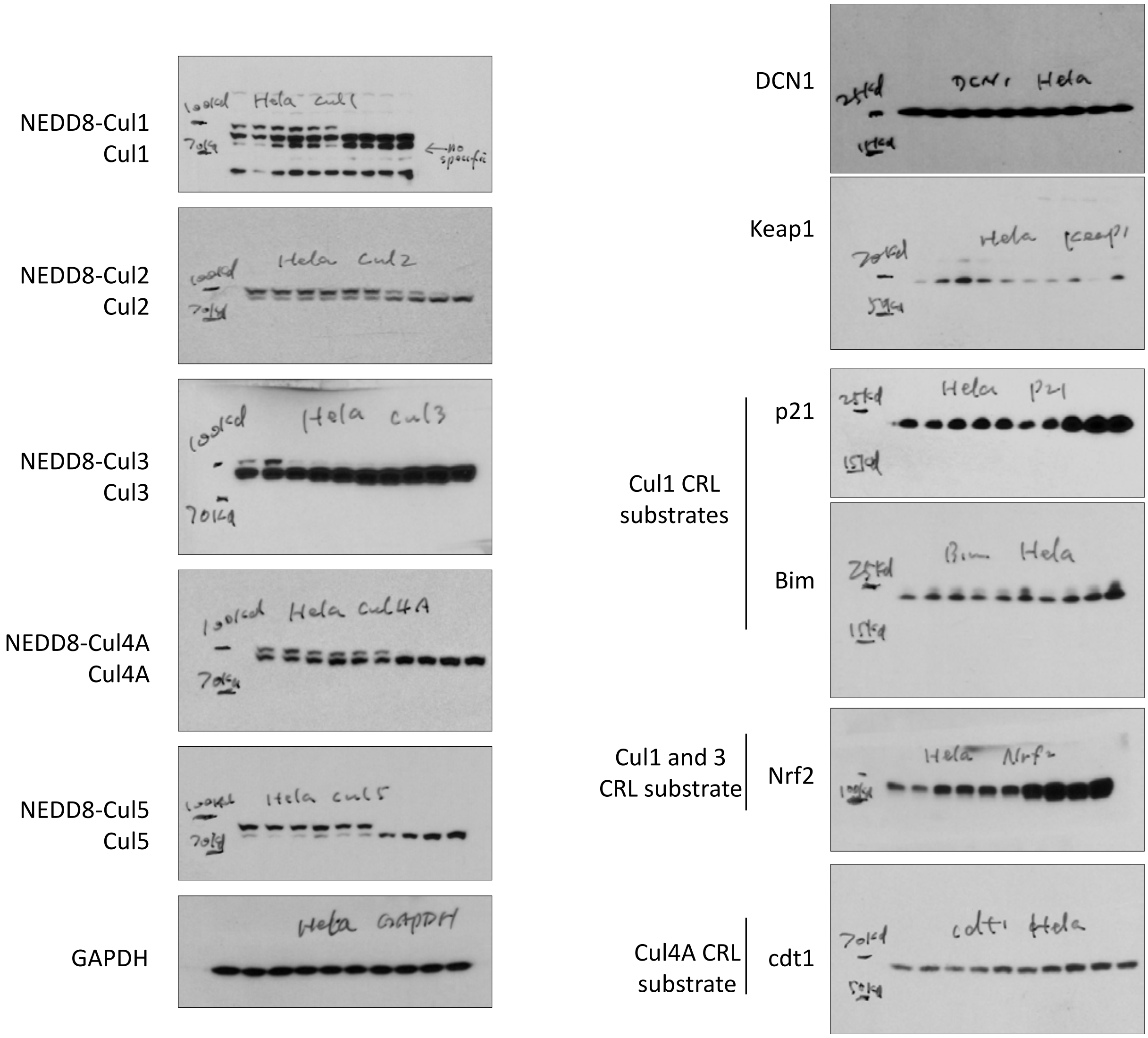

Supplementary Figure 20. Uncropped western blot images for Supplementary Figure 5 (Hela cell line).

Supplementary Figure 21:

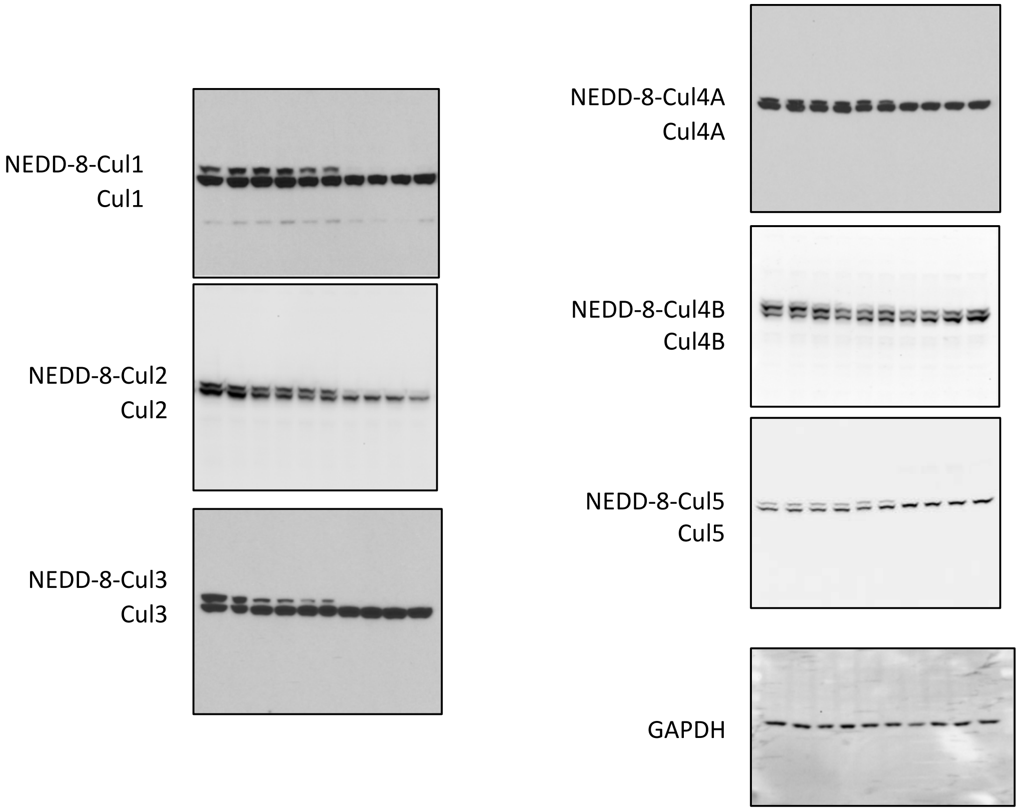

Supplementary Figure 21. Uncropped western blot images for Supplementary Figure 6 (U2OS cell line).

Supplementary Figure 22:

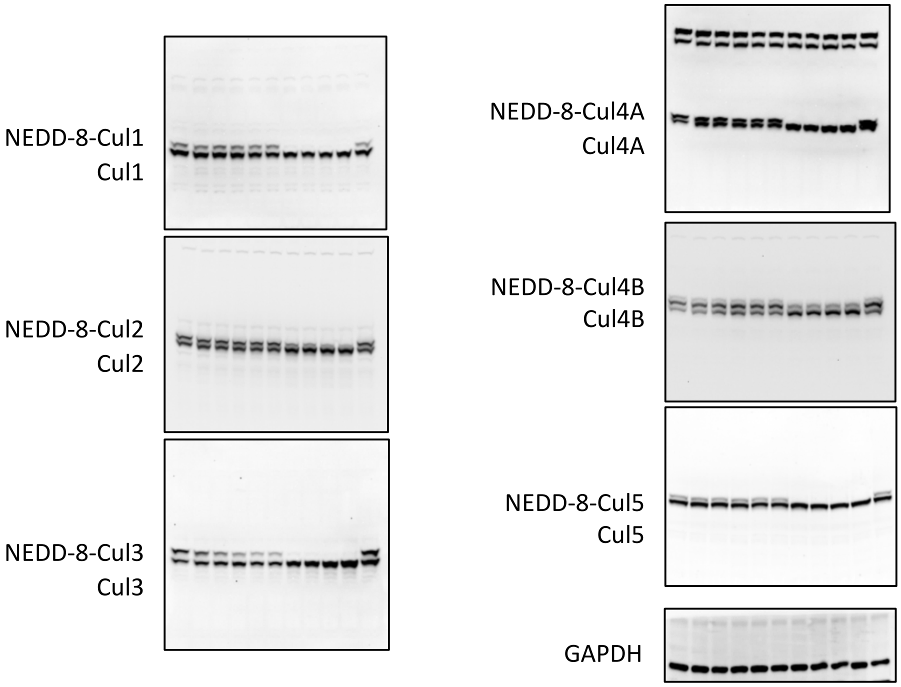

Supplementary Figure 22. Uncropped western blot images for Supplementary Figure 6 (MDA-MB-231 cell line).

Supplementary Figure 23. ^{1}H NMR and ^{13}C NMR for Compound **24**.

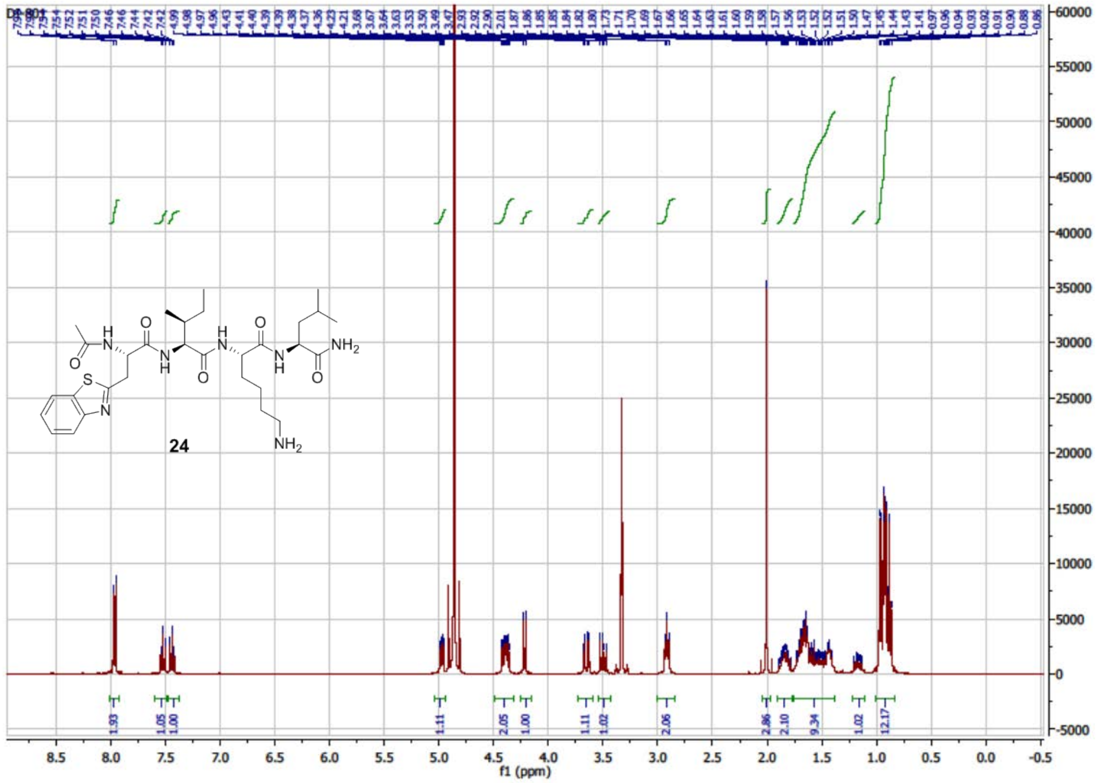

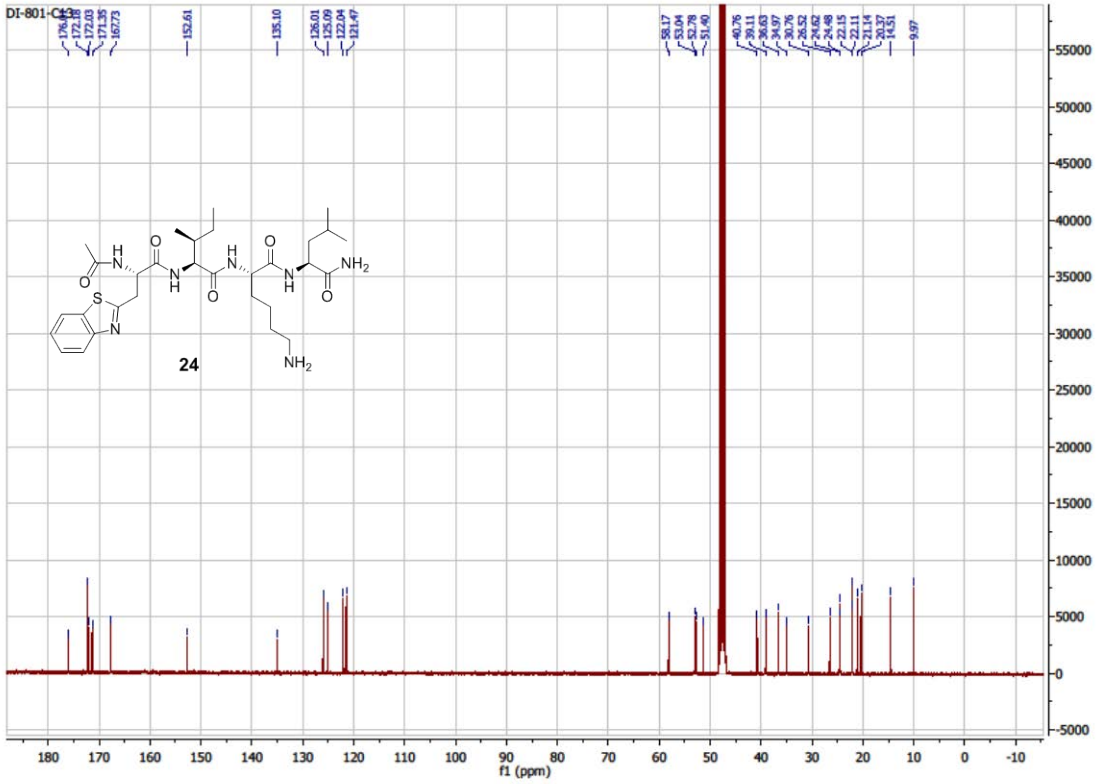

Supplementary Figure 24. ^{1}H NMR and ^{13}C NMR for Compound **25**.

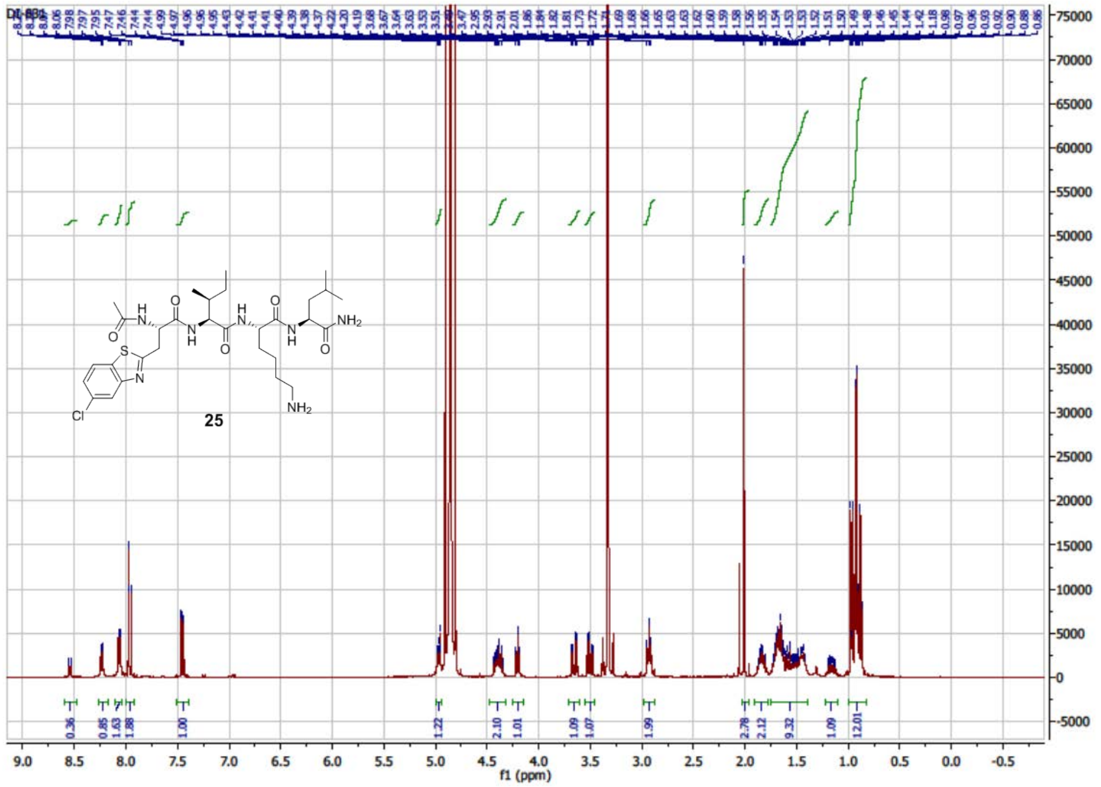

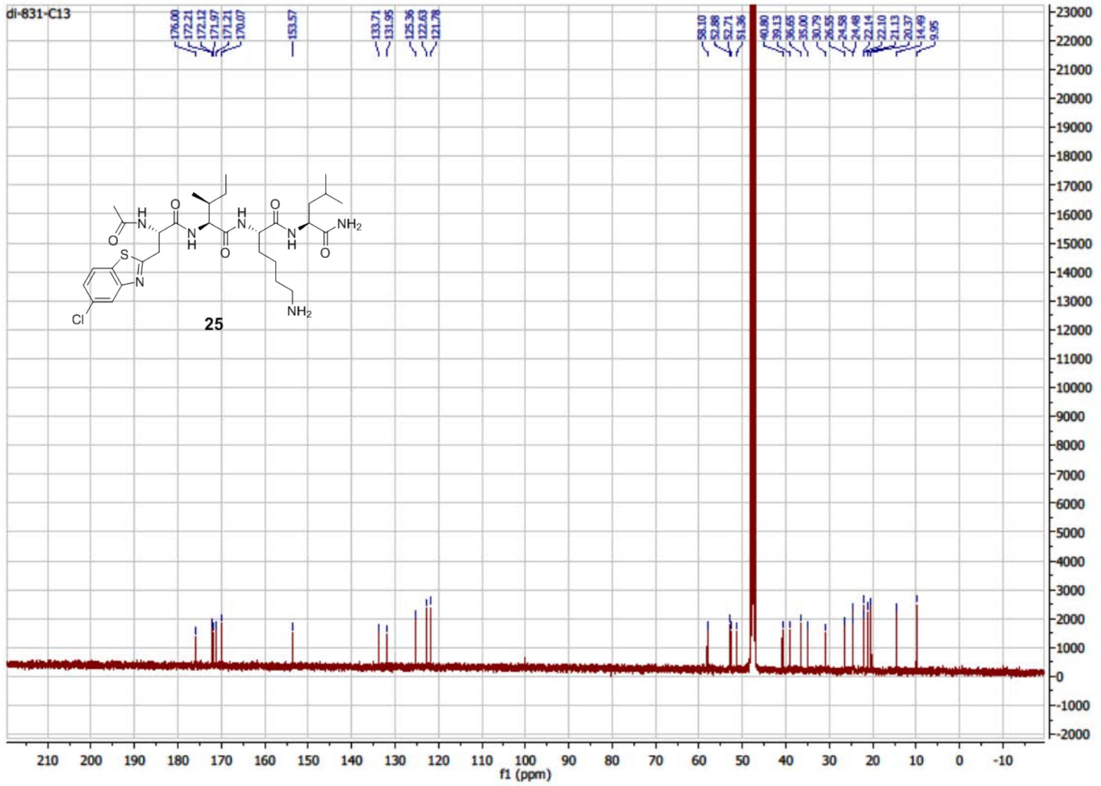

Supplementary Figure 25. ^{1}H NMR and ^{13}C NMR for Compound **26**.

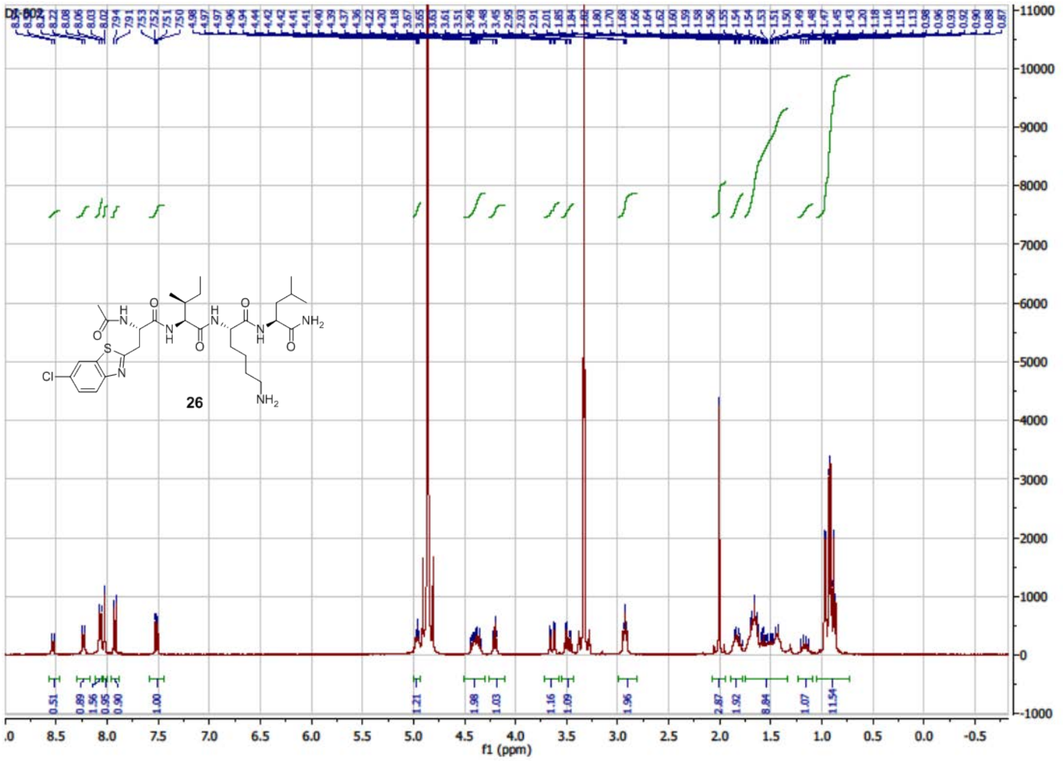

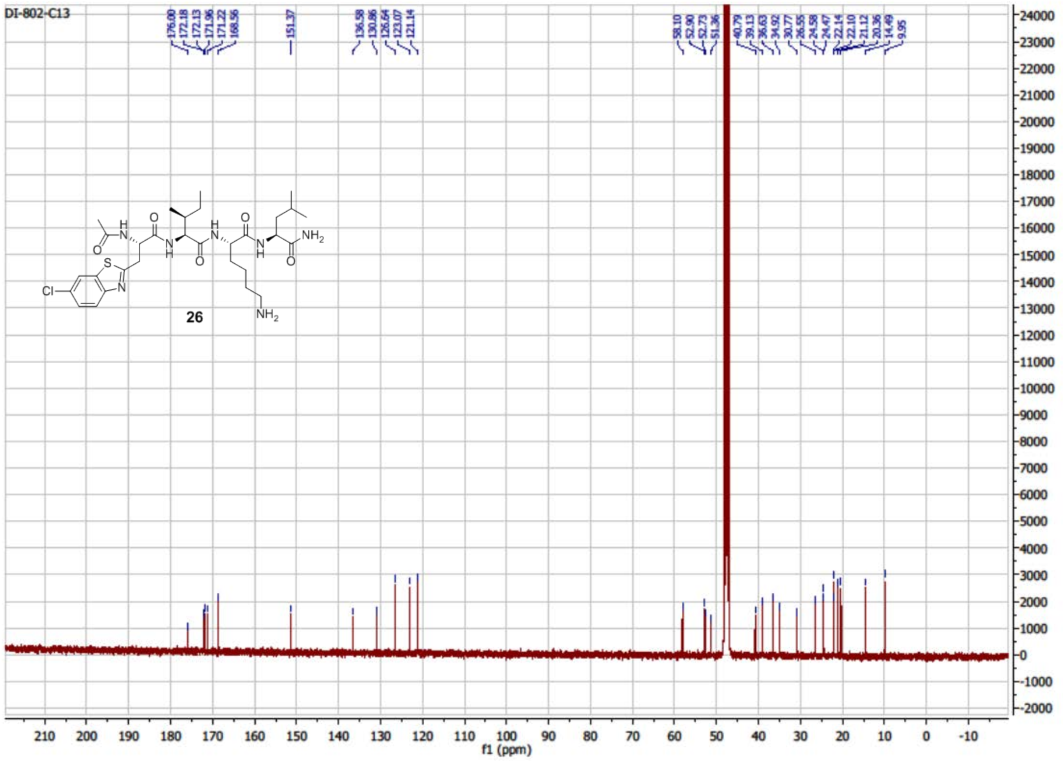

Supplementary Figure 26. ^{1}H NMR and ^{13}C NMR for Compound **27**.

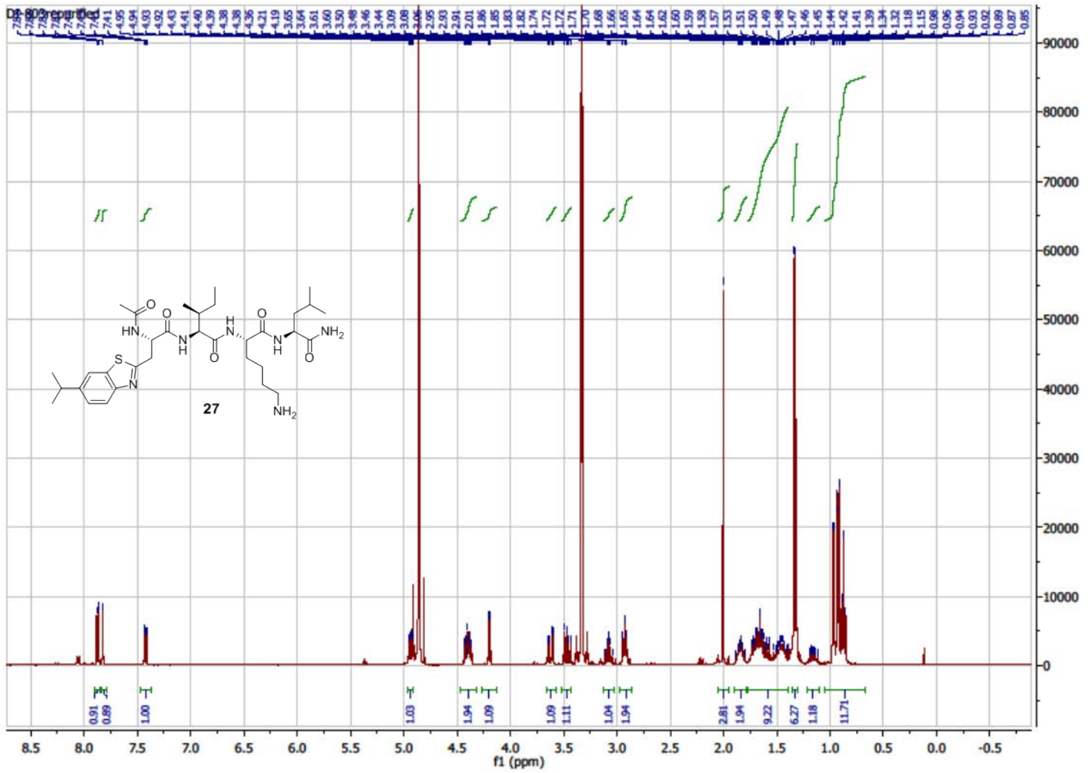

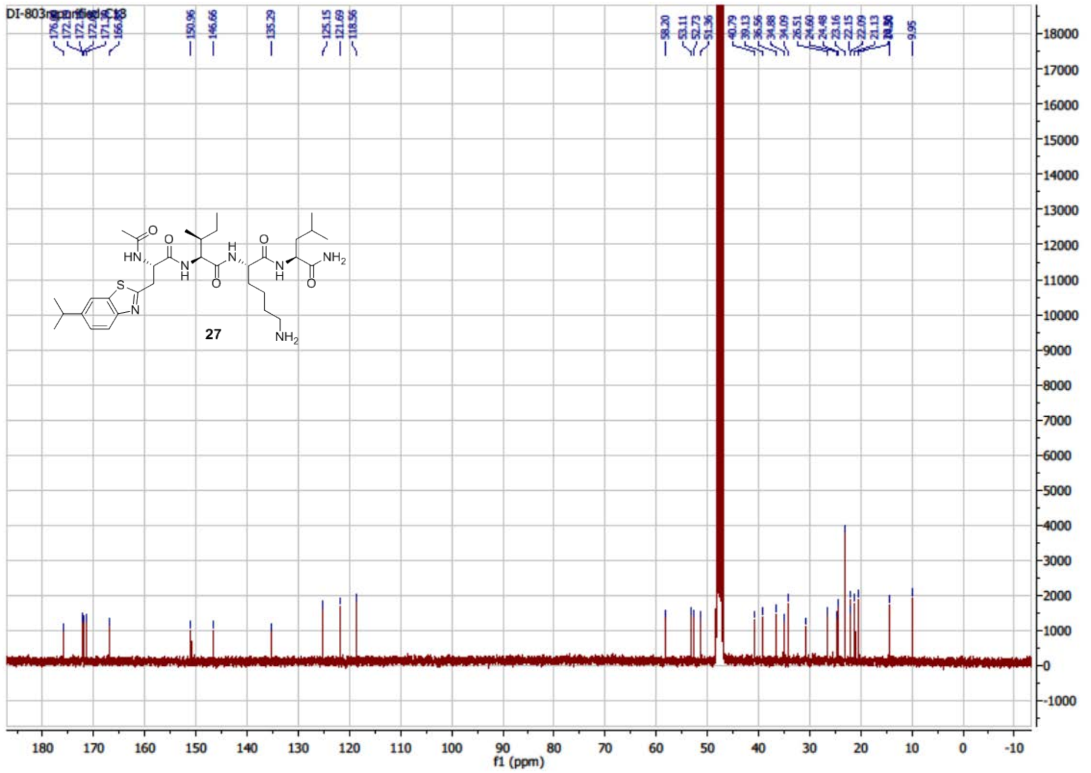

Supplementary Figure 27. ^{1}H NMR and ^{13}C NMR for Compound **28**.

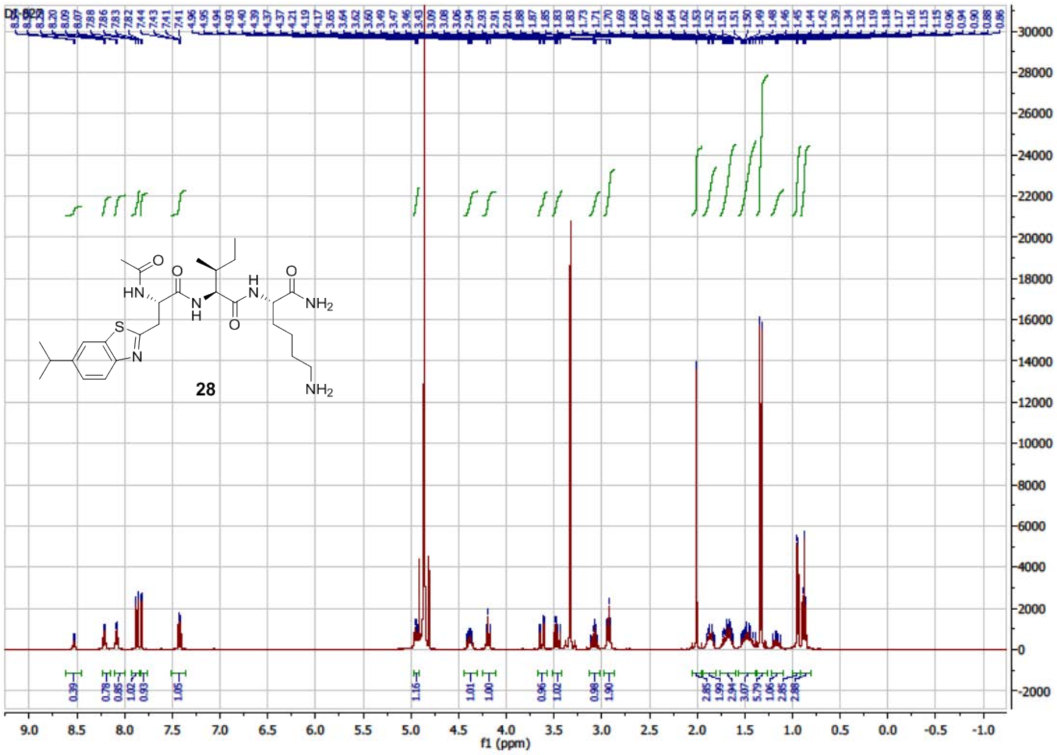

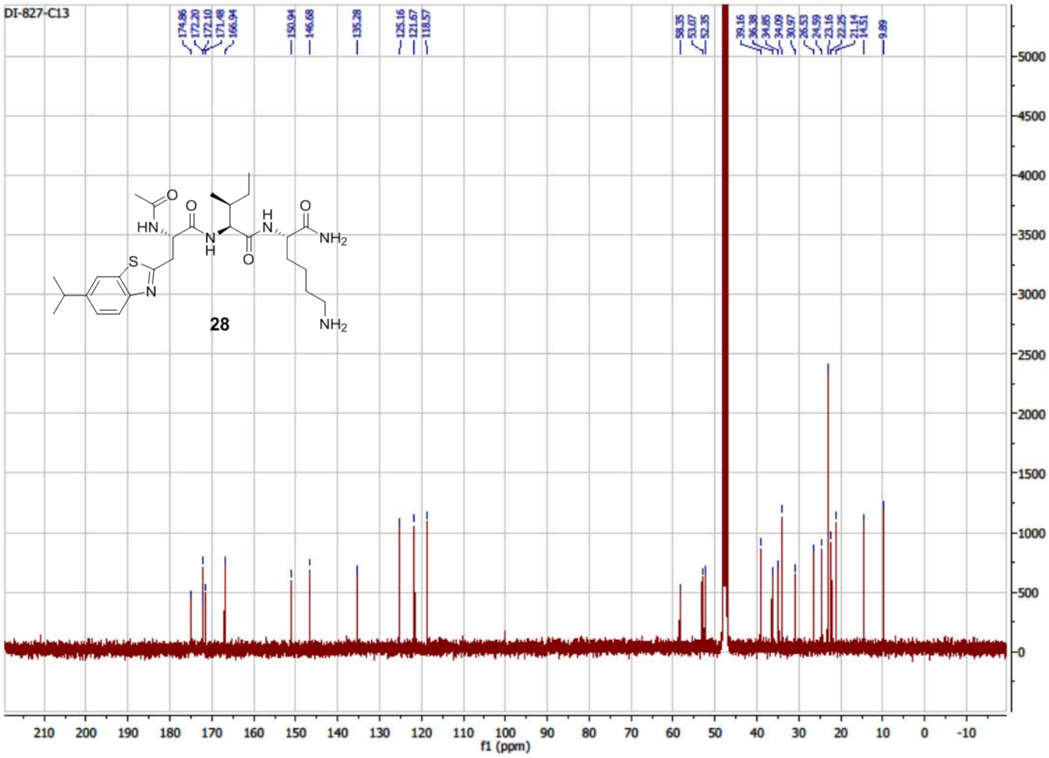

Supplementary Figure 28. ^{1}H NMR and ^{13}C NMR for Compound **29**.

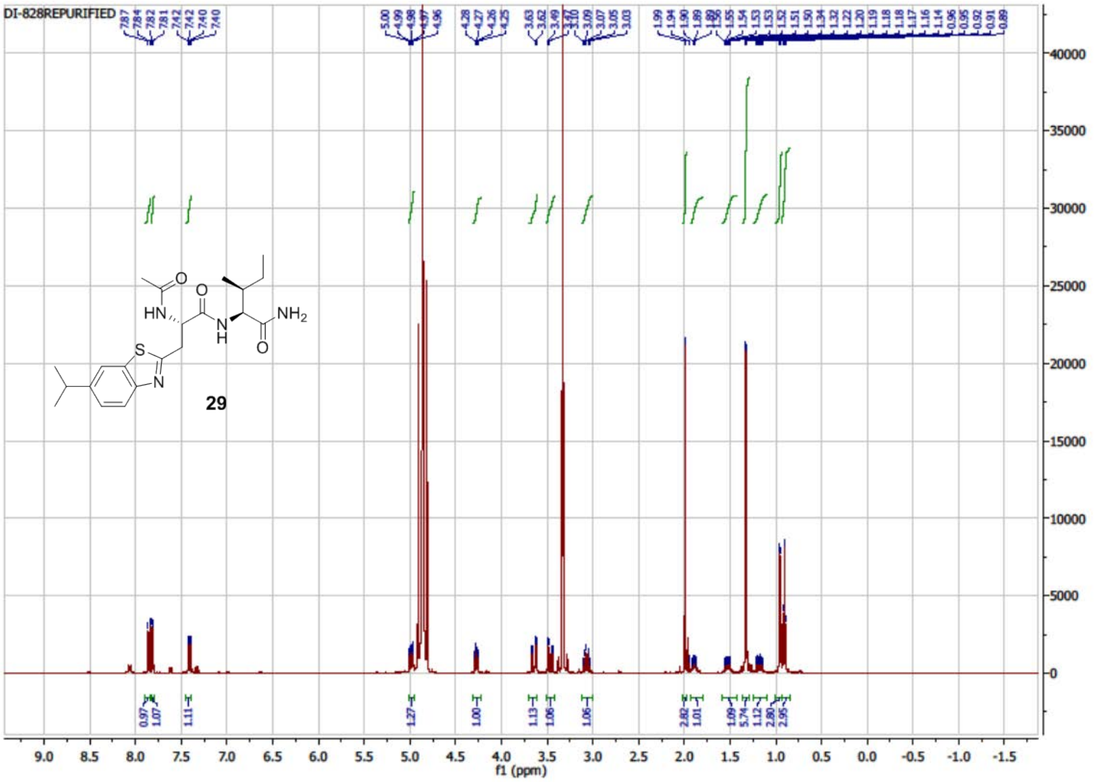

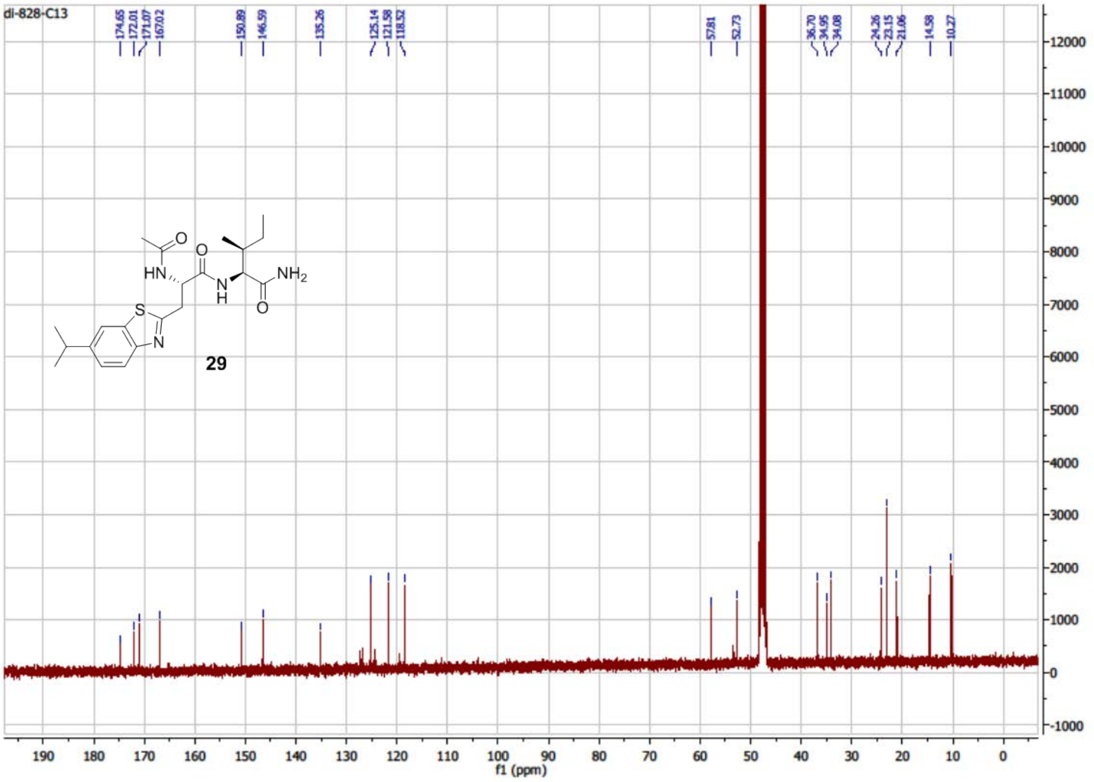

Supplementary Figure 29. ^{1}H NMR and ^{13}C NMR for Compound **30**.

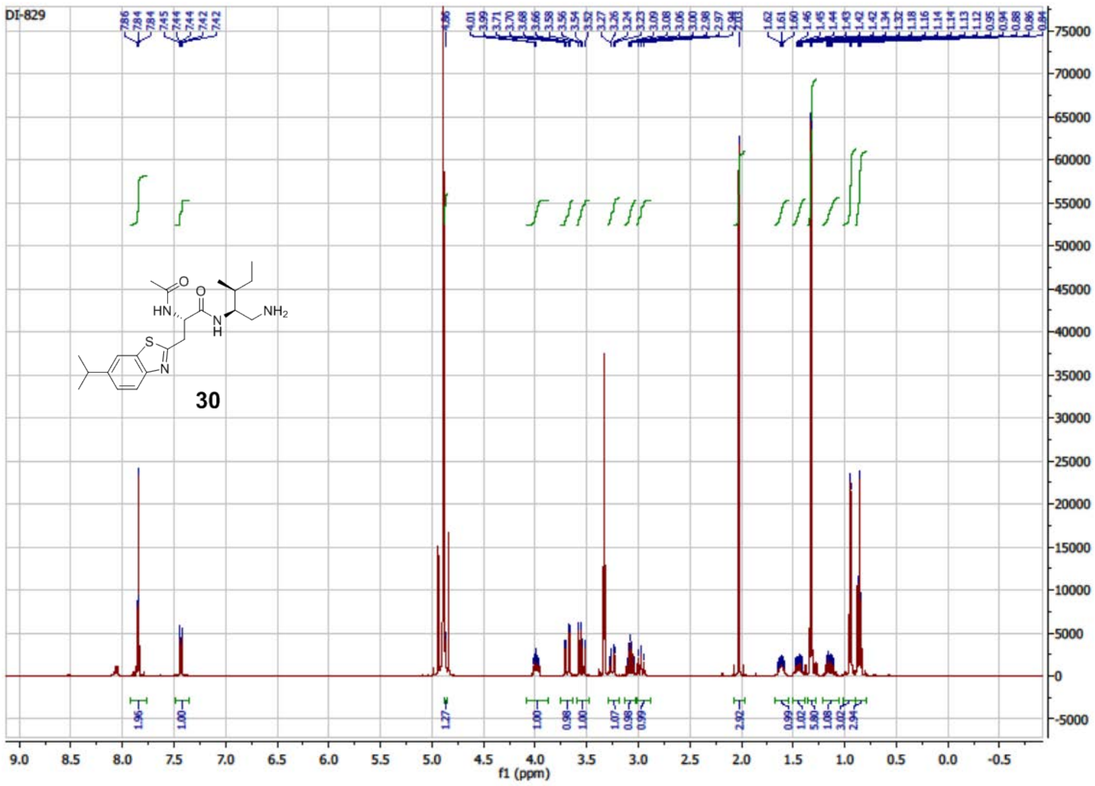

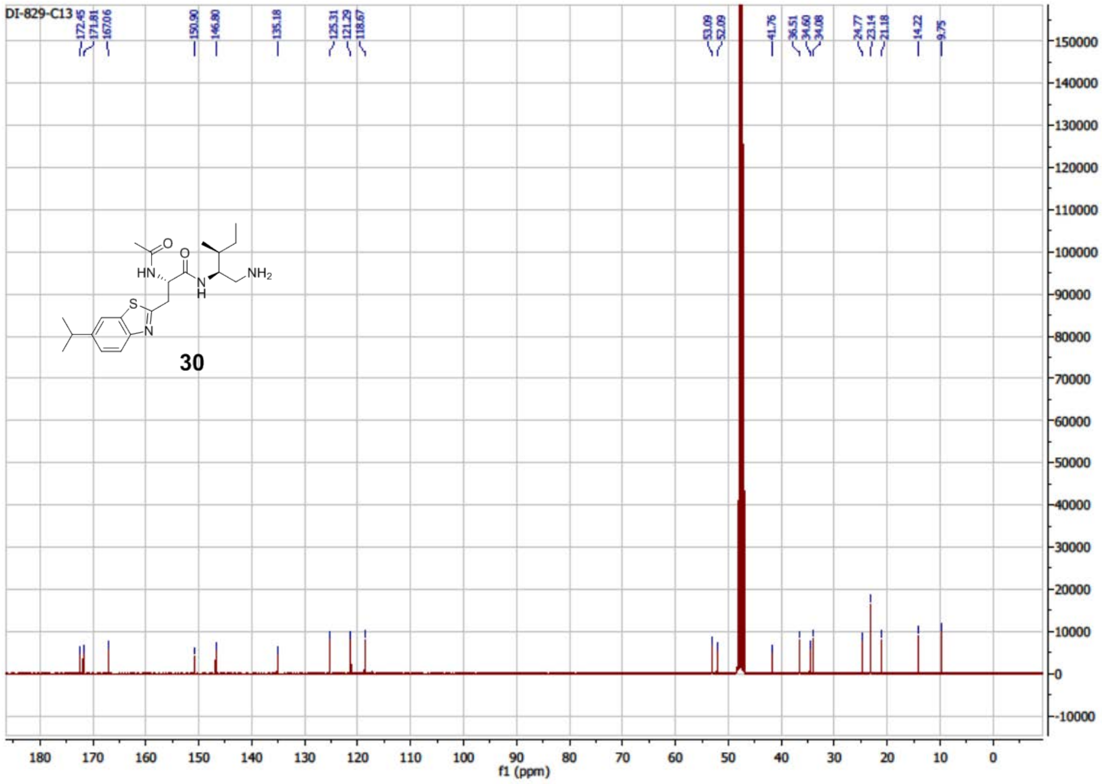

Supplementary Figure 30. ^{1}H NMR and ^{13}C NMR for Compound **31**.

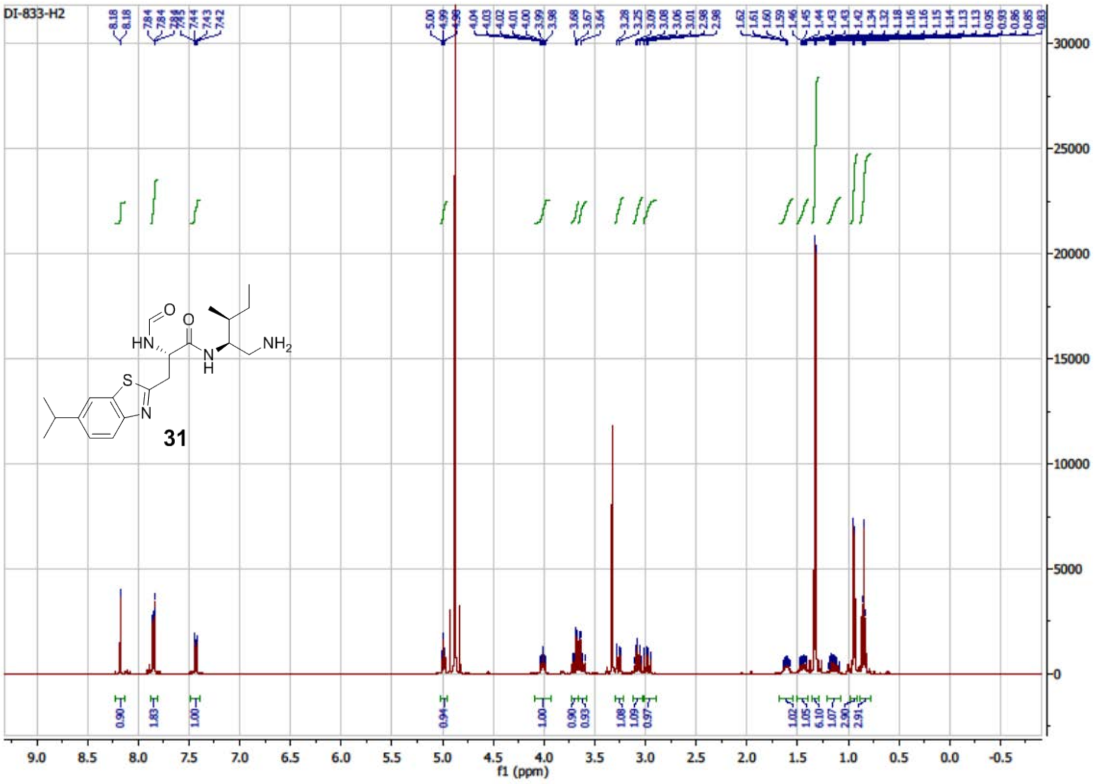

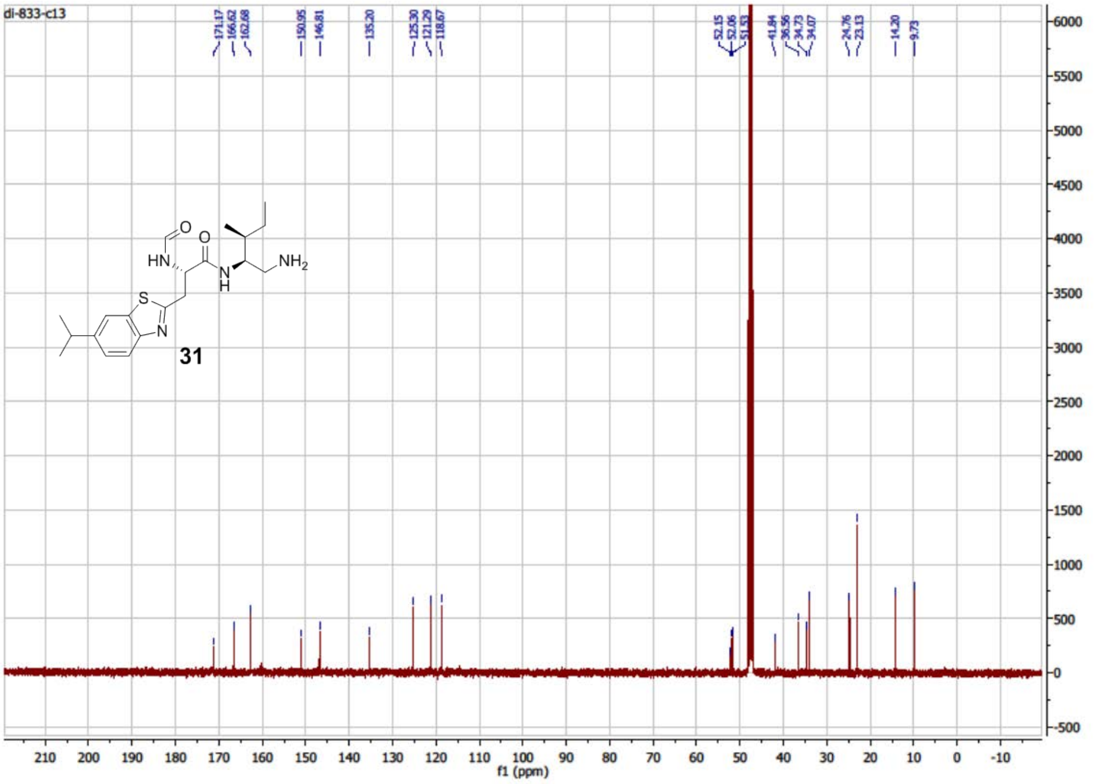

Supplementary Figure 31. ^{1}H NMR and ^{13}C NMR for Compound **32**.

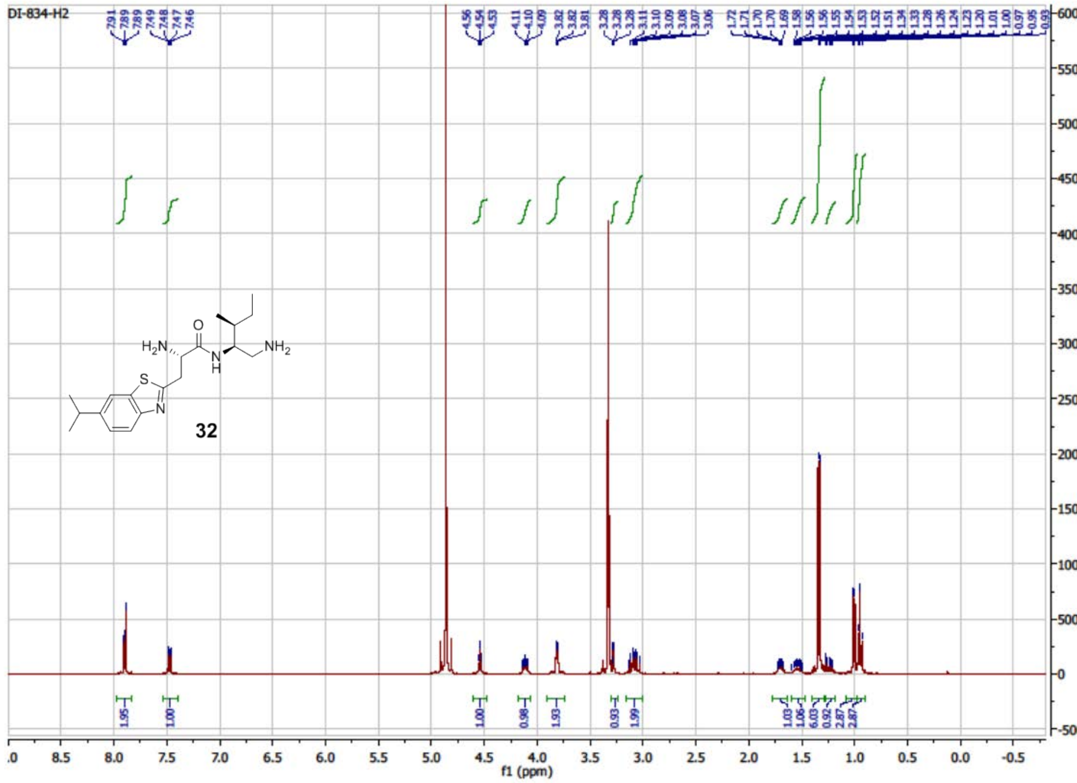

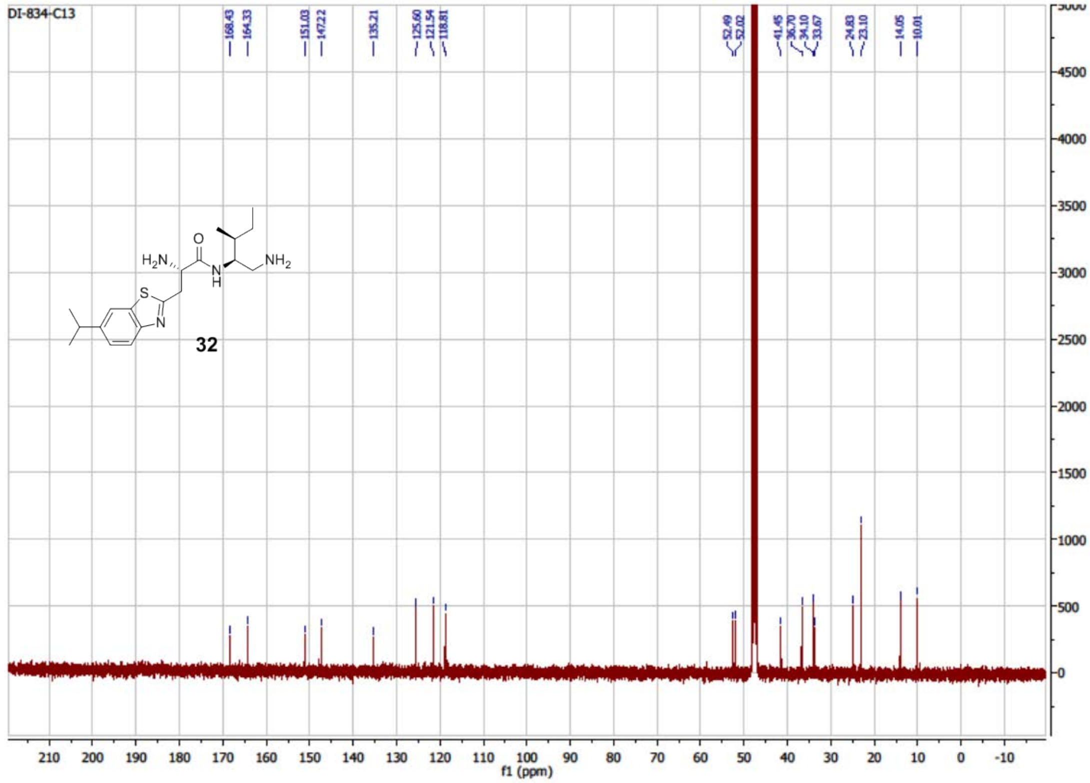

Supplementary Figure 32. ^{1}H NMR and ^{13}C NMR for Compound **33**.

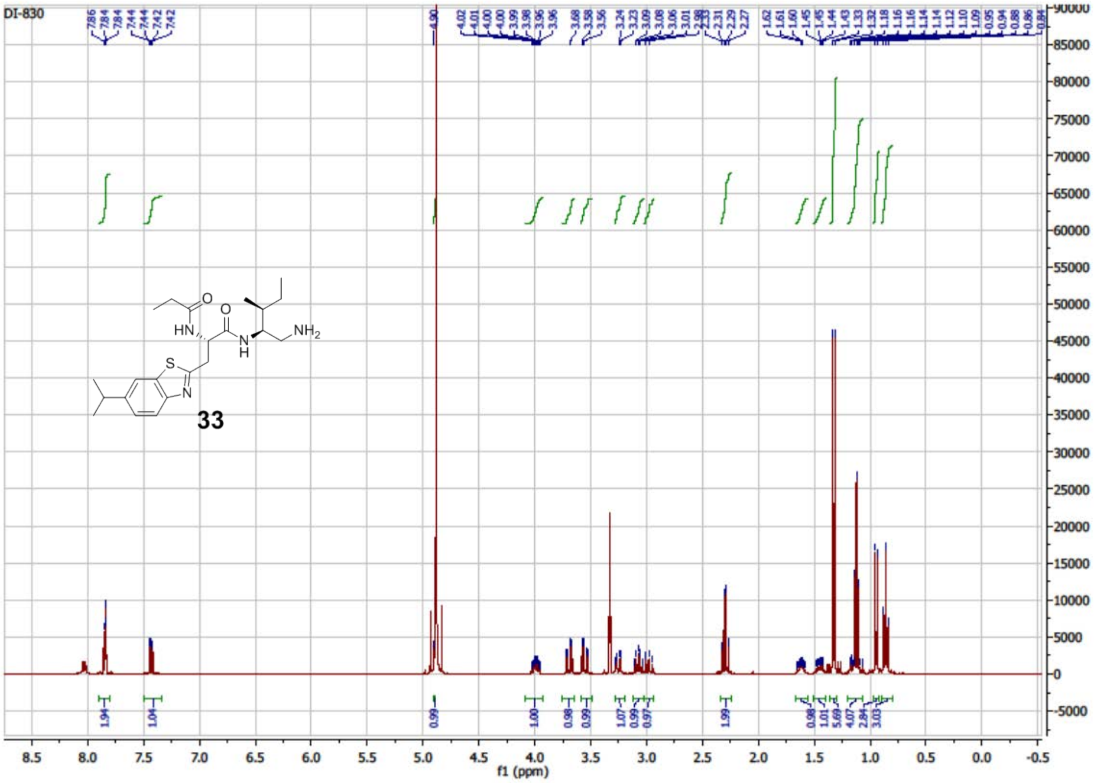

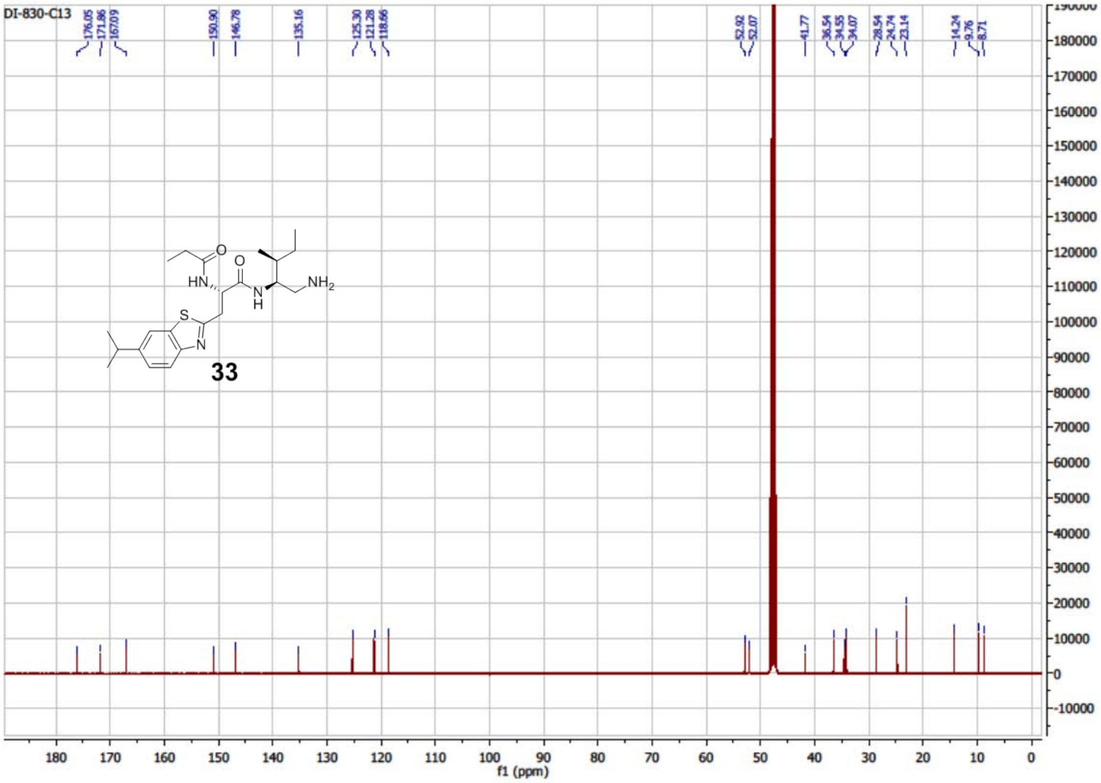

1 **Supplementary Figure 33**. ^{1}H NMR and ^{13}C NMR for Compound **34**.

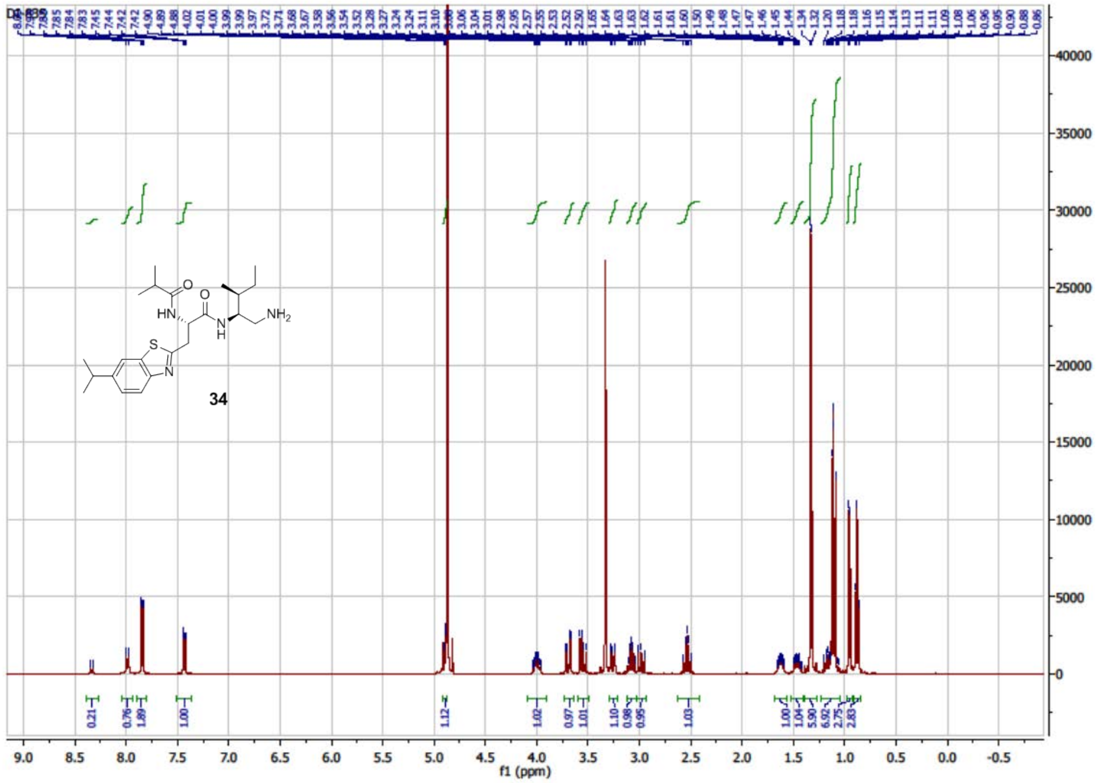

2

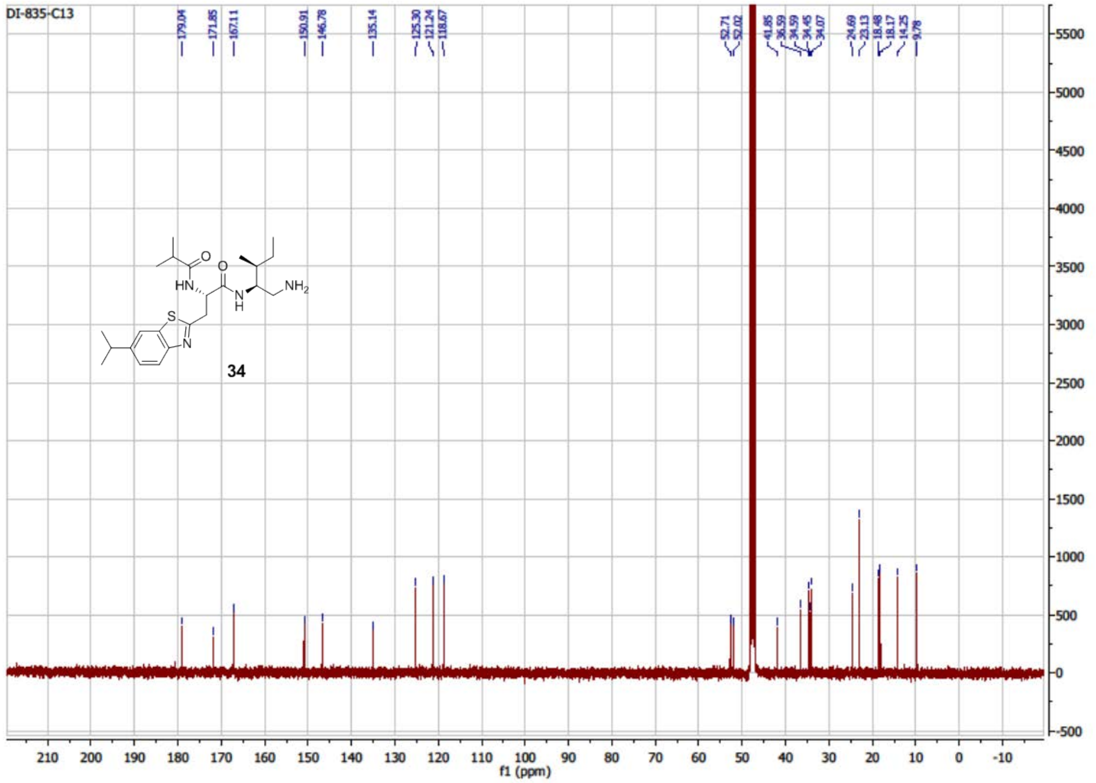

3

Supplementary Figure 34. ^{1}H NMR and ^{13}C NMR for Compound **35**.

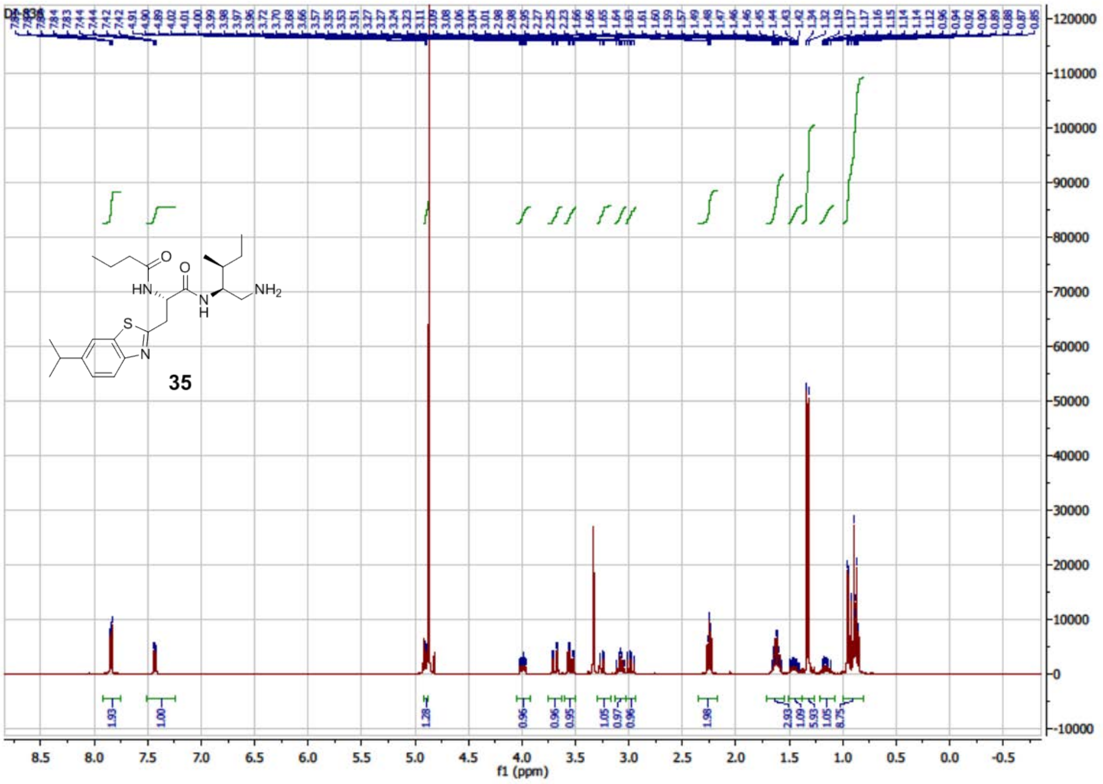

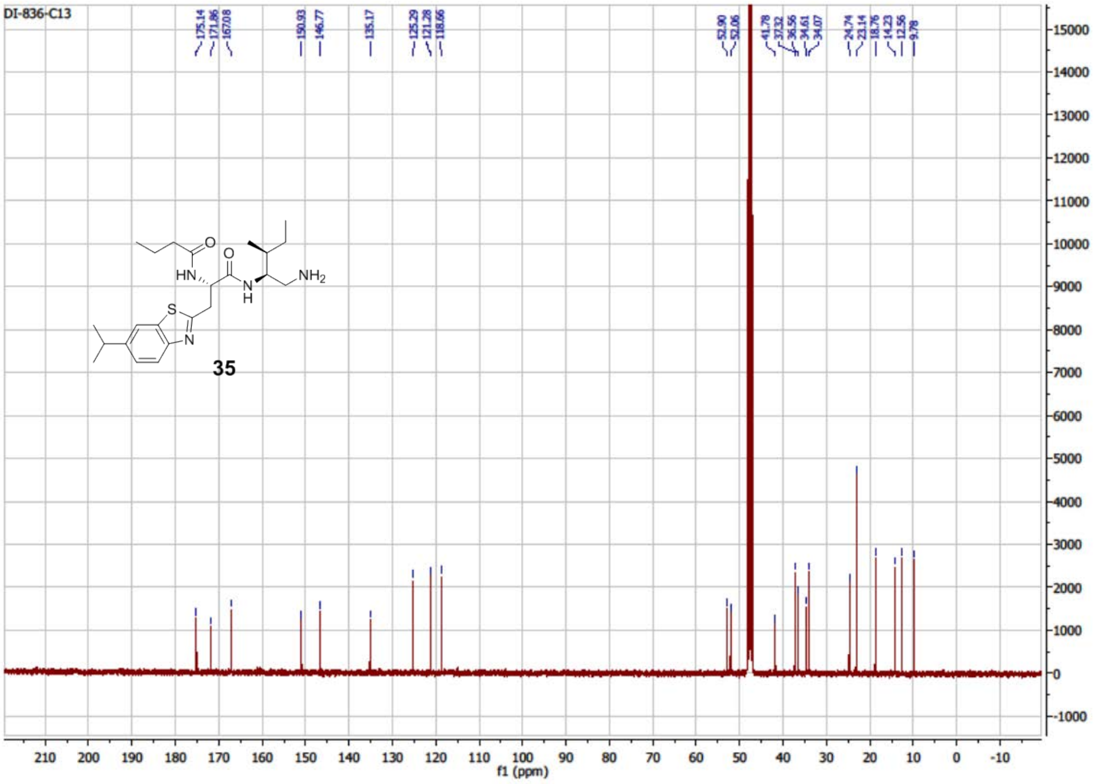

Supplementary Figure 35. ^{1}H NMR and ^{13}C NMR for Compound **36**.

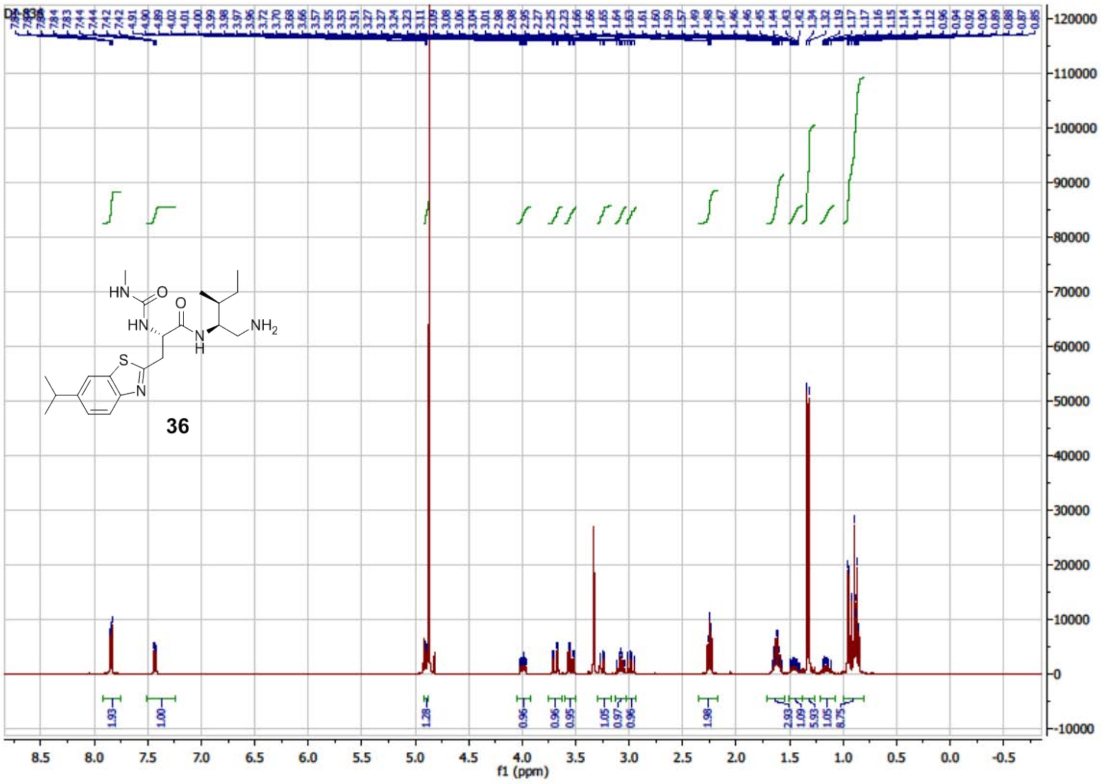

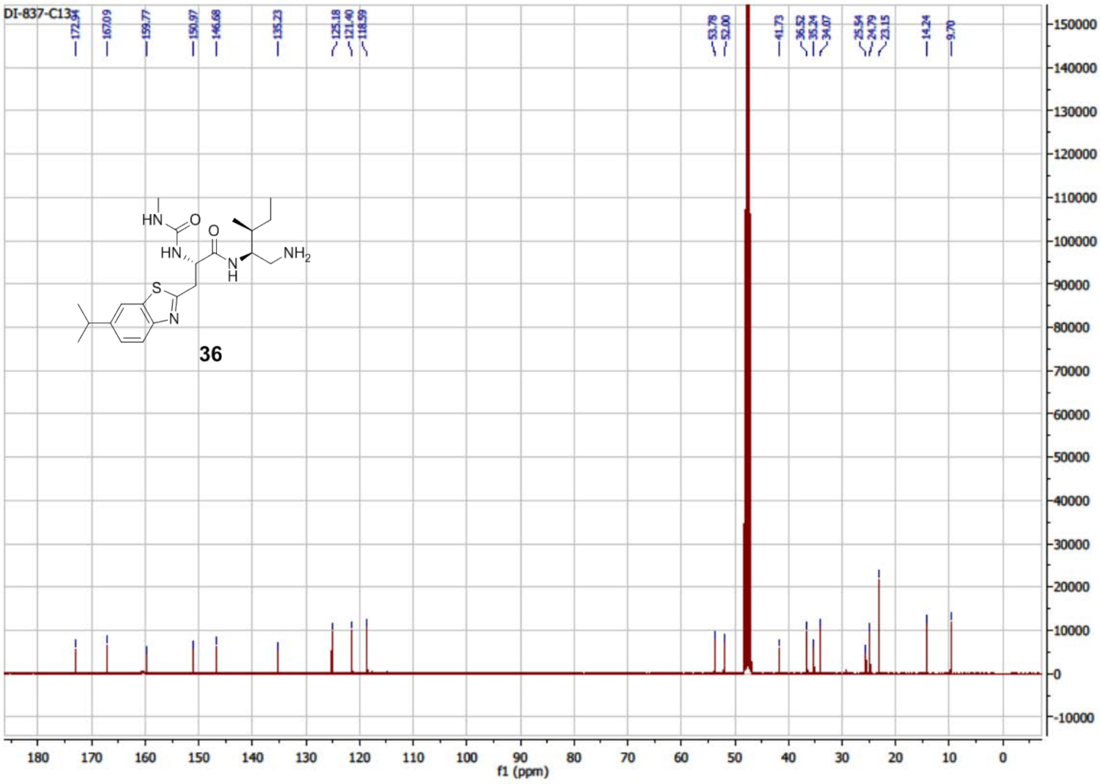

Supplementary Figure 36. ^{1}H NMR and ^{13}C NMR for Compound **37**.

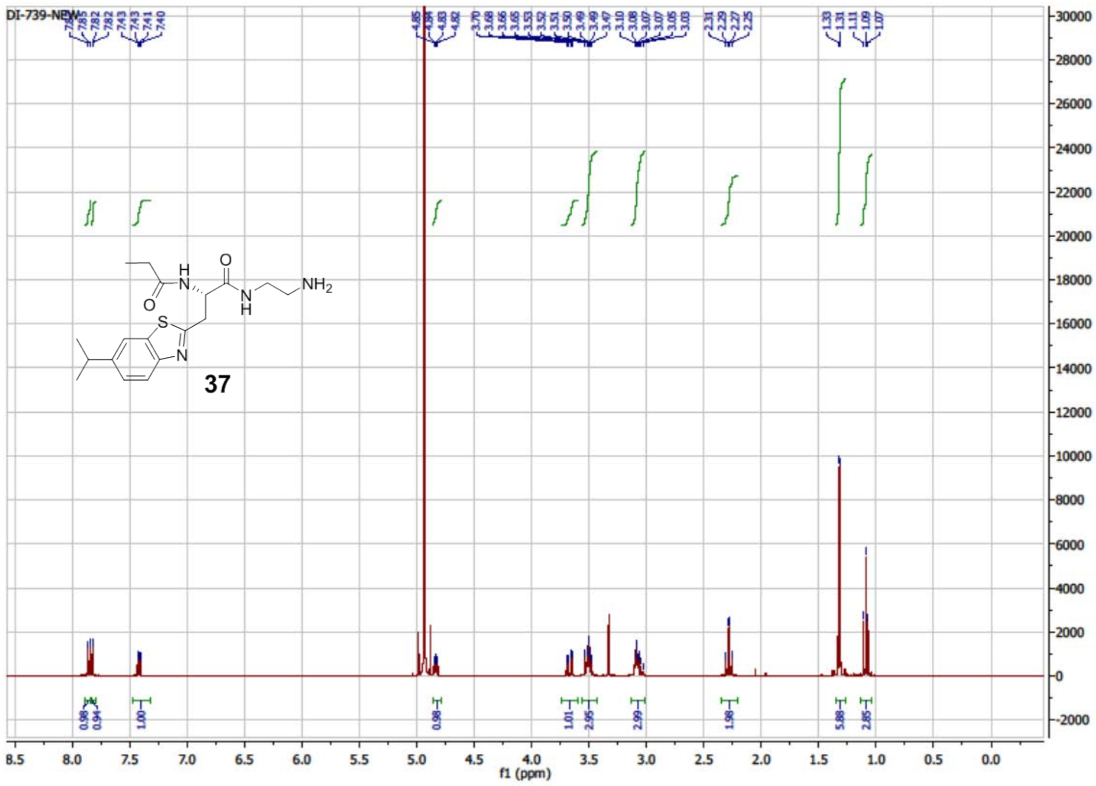

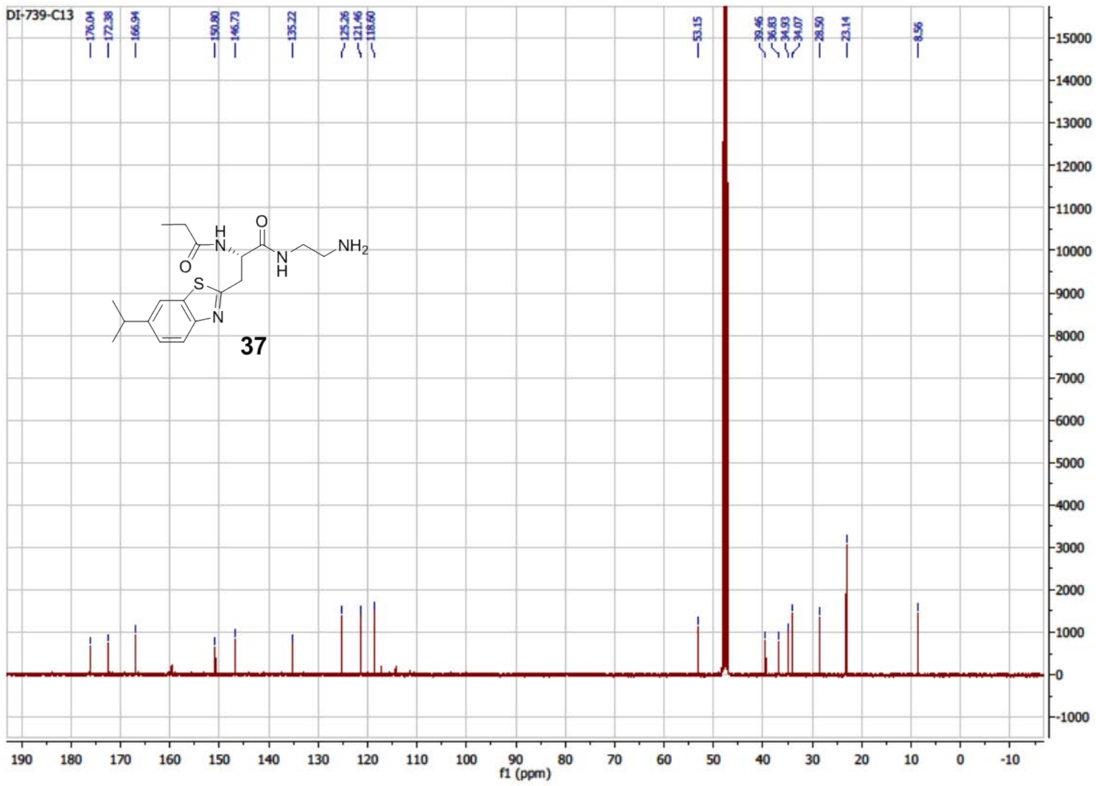

Supplementary Figure 37. ^{1}H NMR and ^{13}C NMR for Compound **38**.

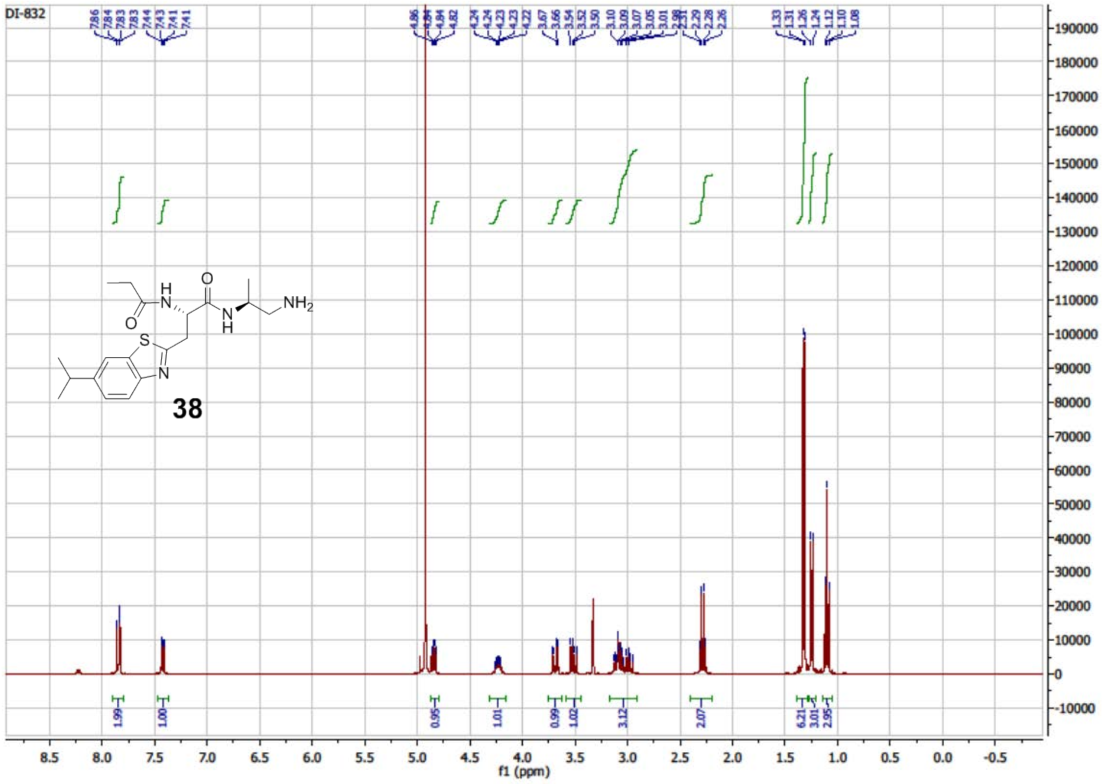

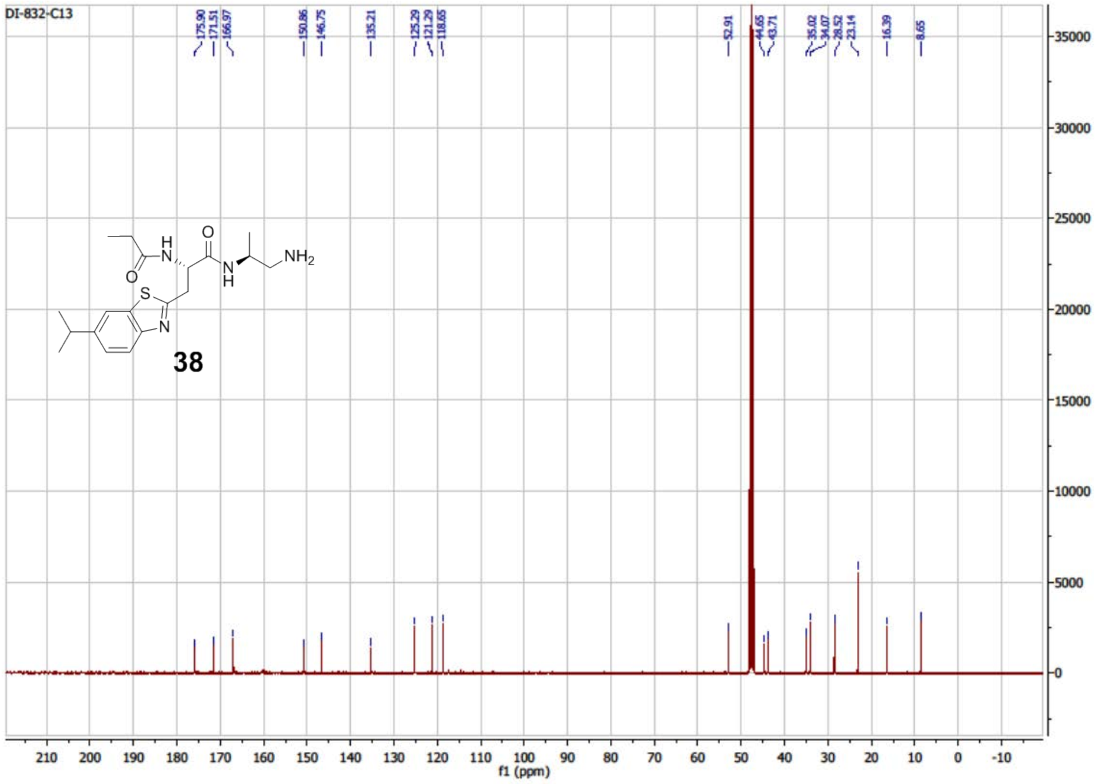

Supplementary Figure 38. ^{1}H NMR and ^{13}C NMR for Compound **39**.

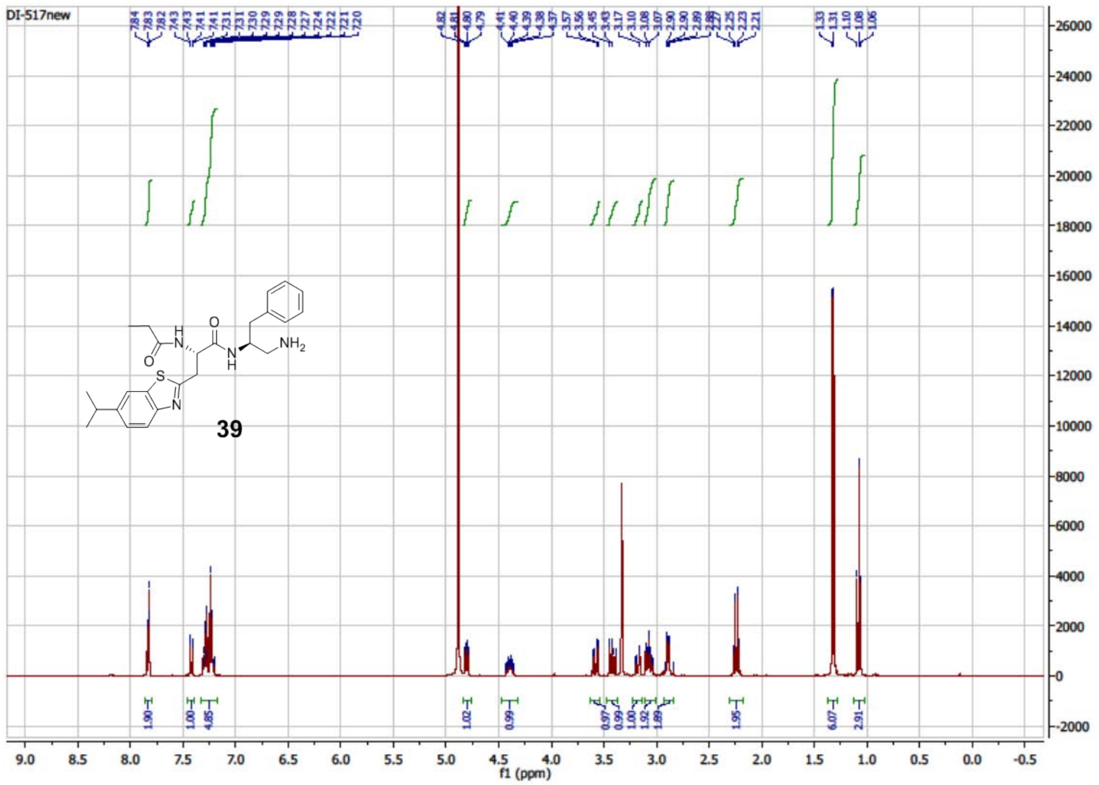

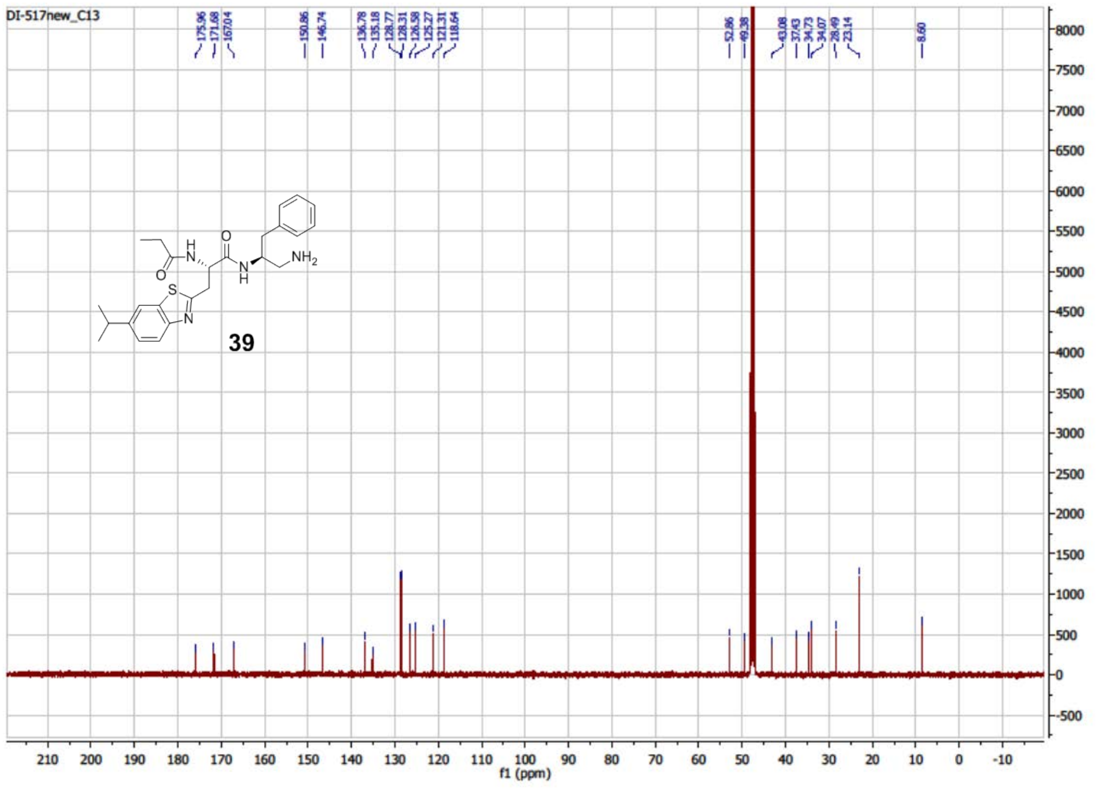

Supplementary Figure 39. ^{1}H NMR and ^{13}C NMR for Compound **40**.

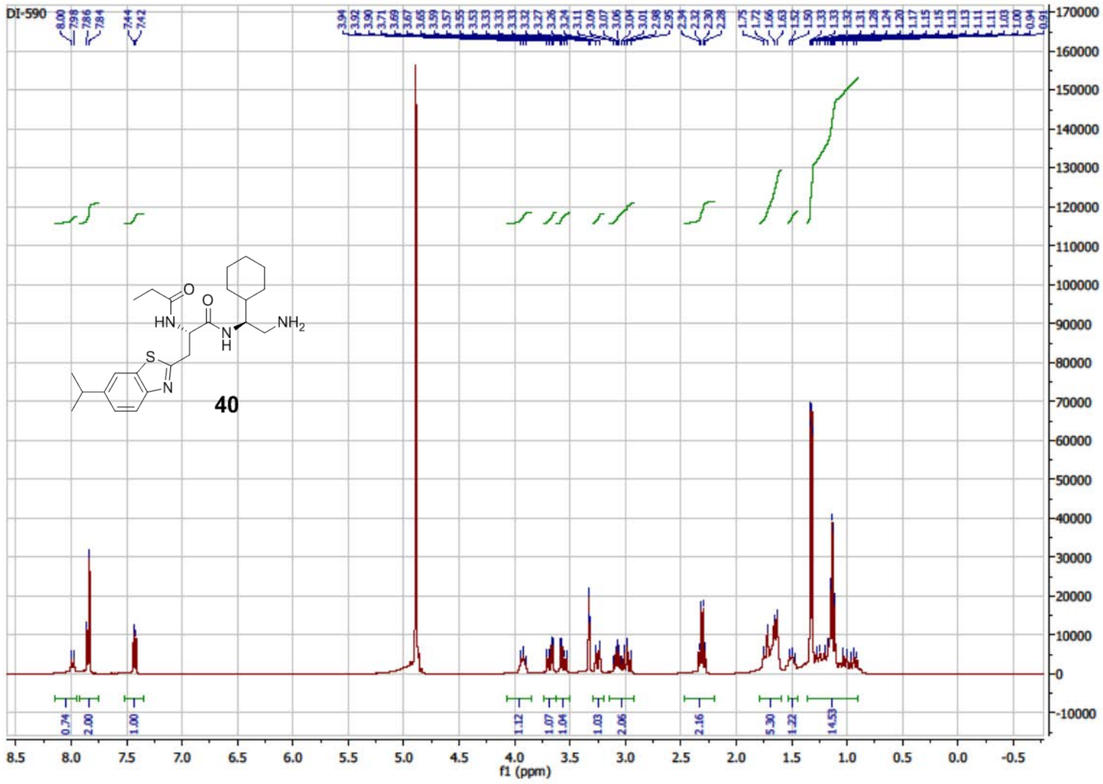

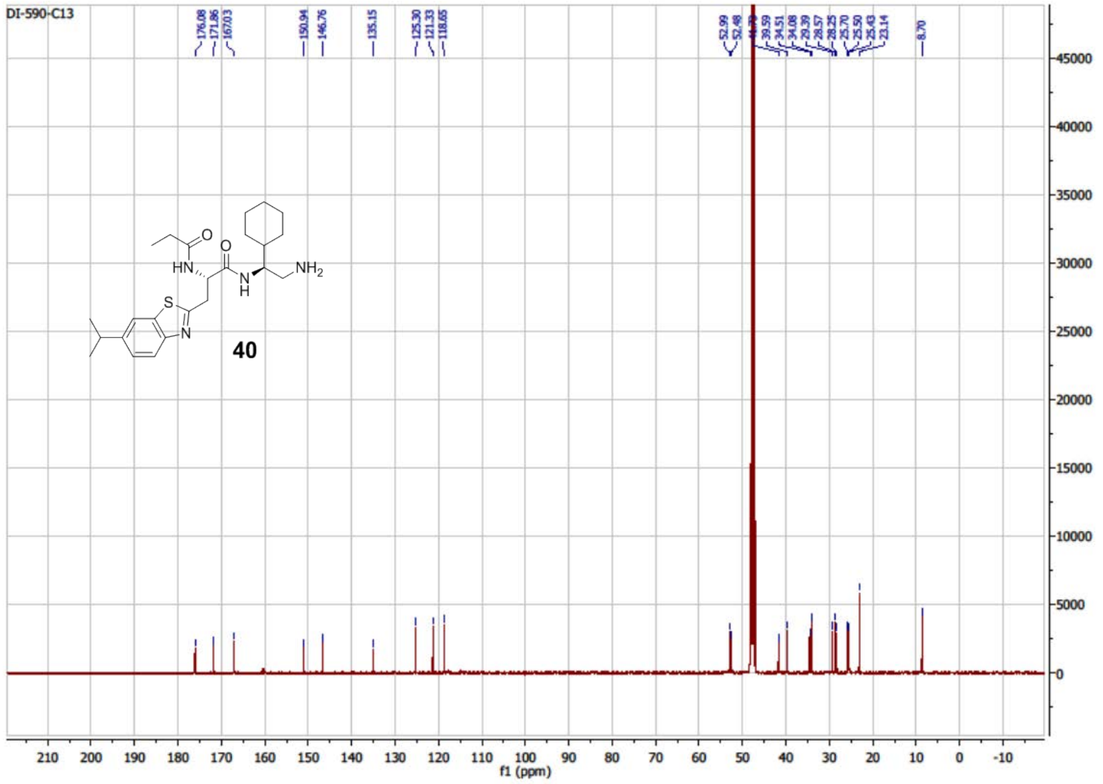

Supplementary Figure 40. ^{1}H NMR and ^{13}C NMR for Compound **41**.

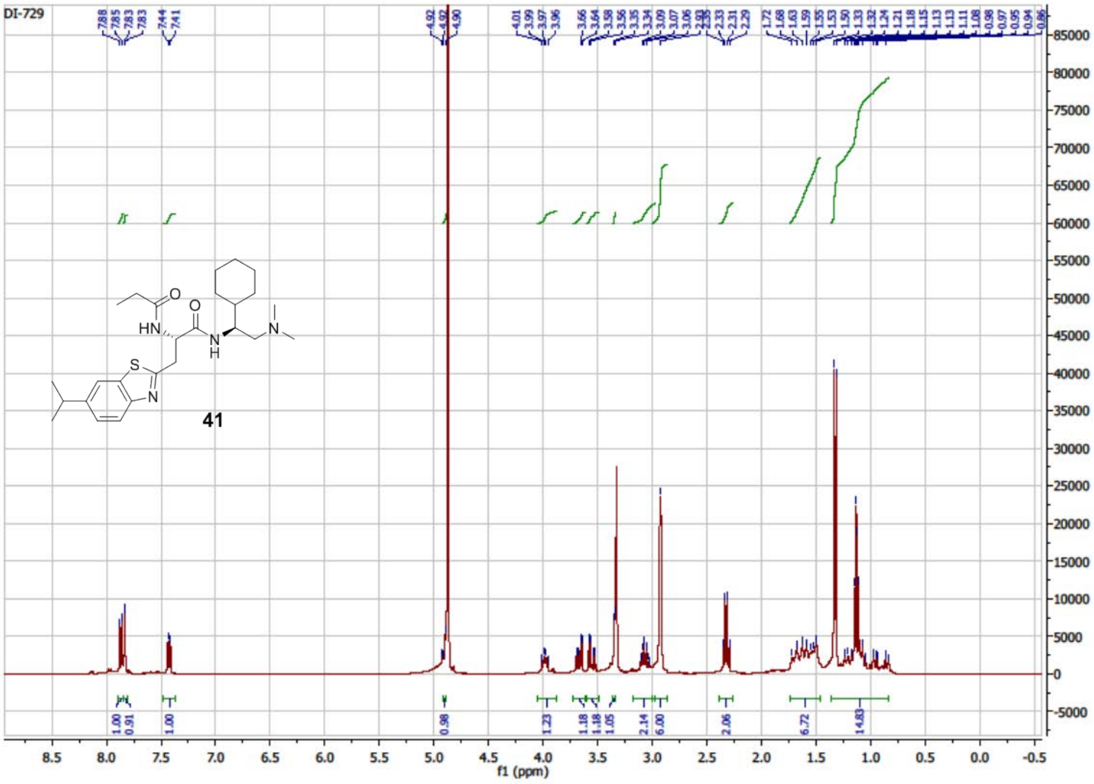

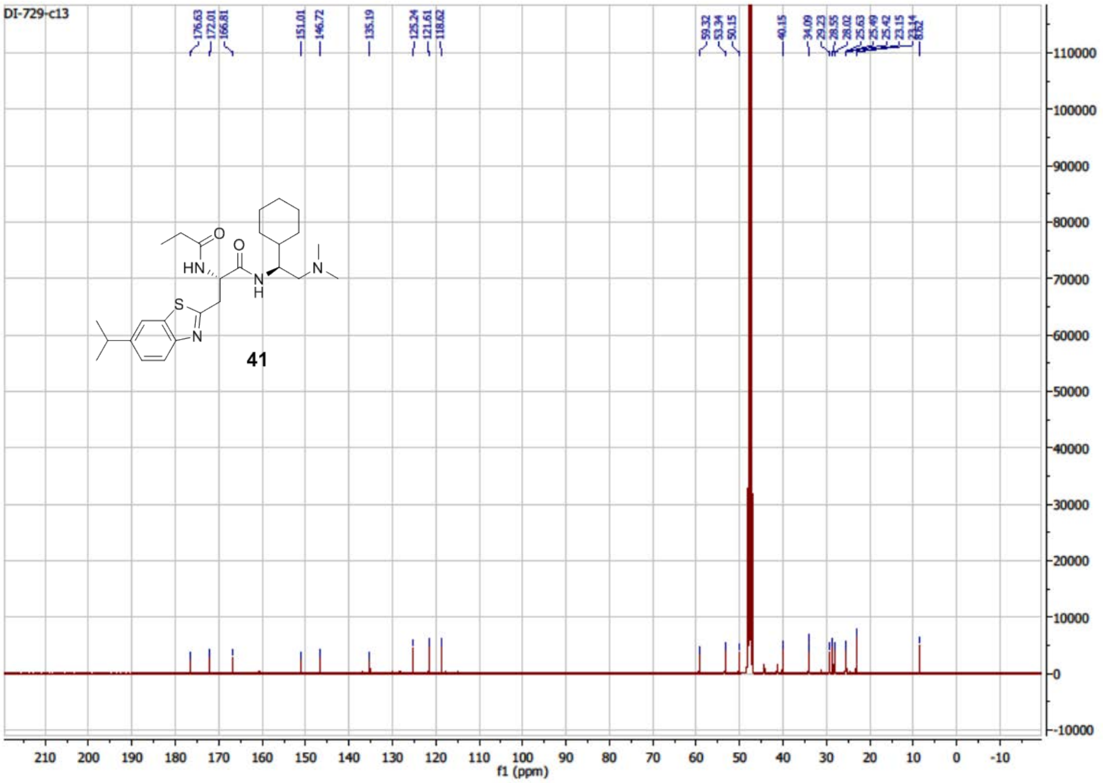

Supplementary Figure 41. ^{1}H NMR and ^{13}C NMR for Compound **42**.

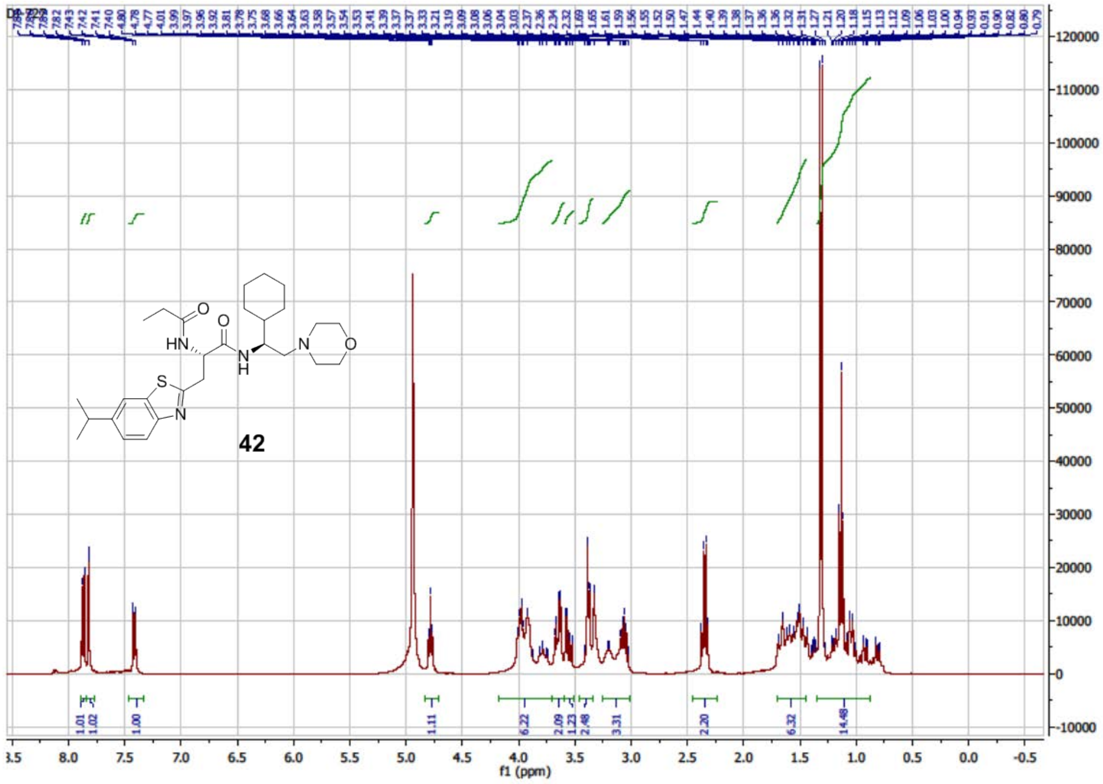

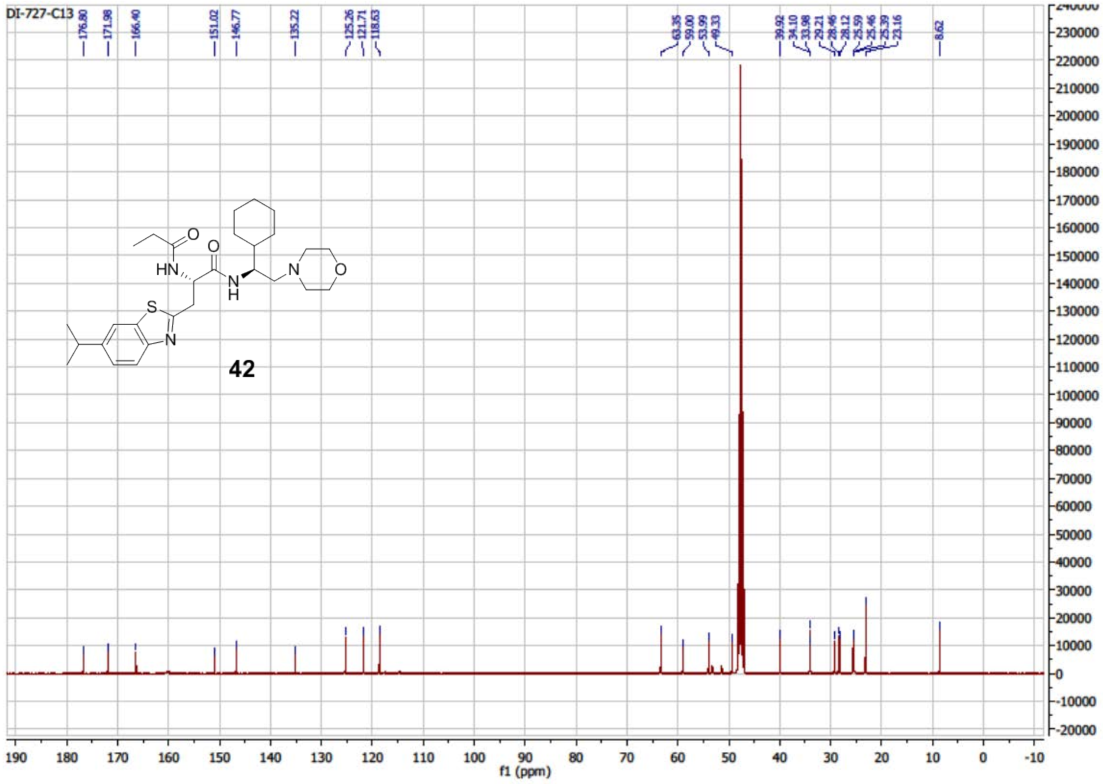

Supplementary Figure 42. ^{1}H NMR and ^{13}C NMR for Compound **43**.

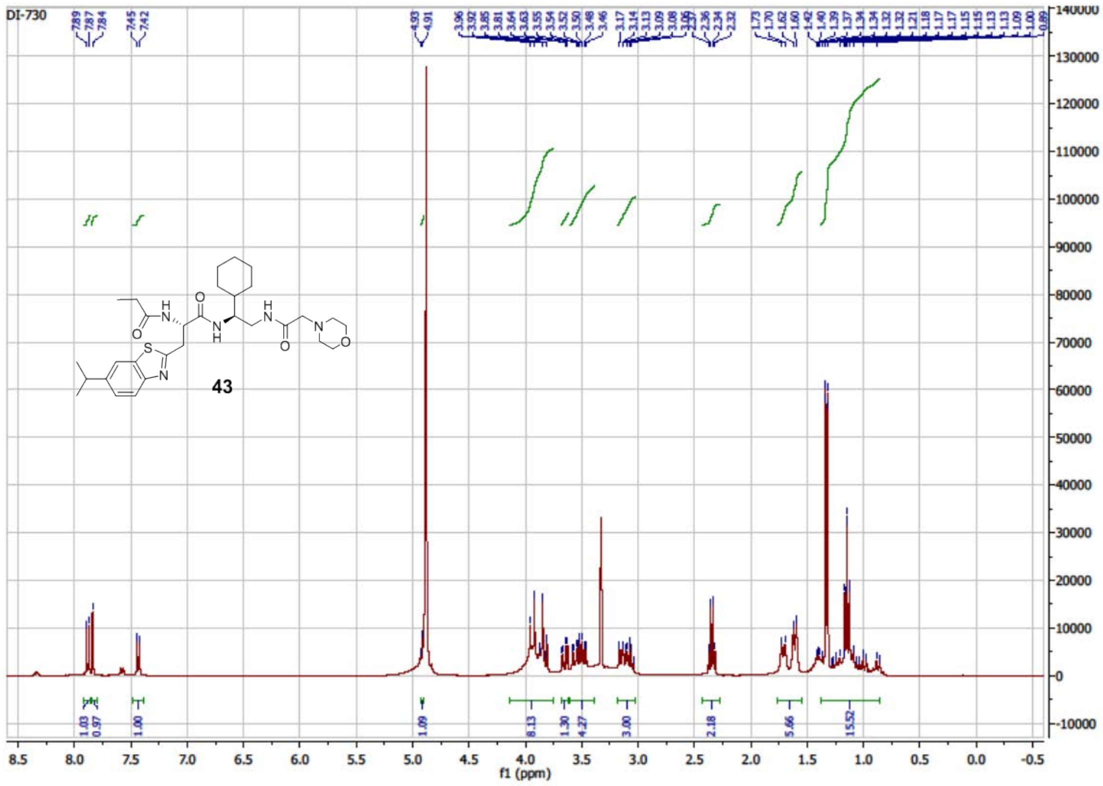

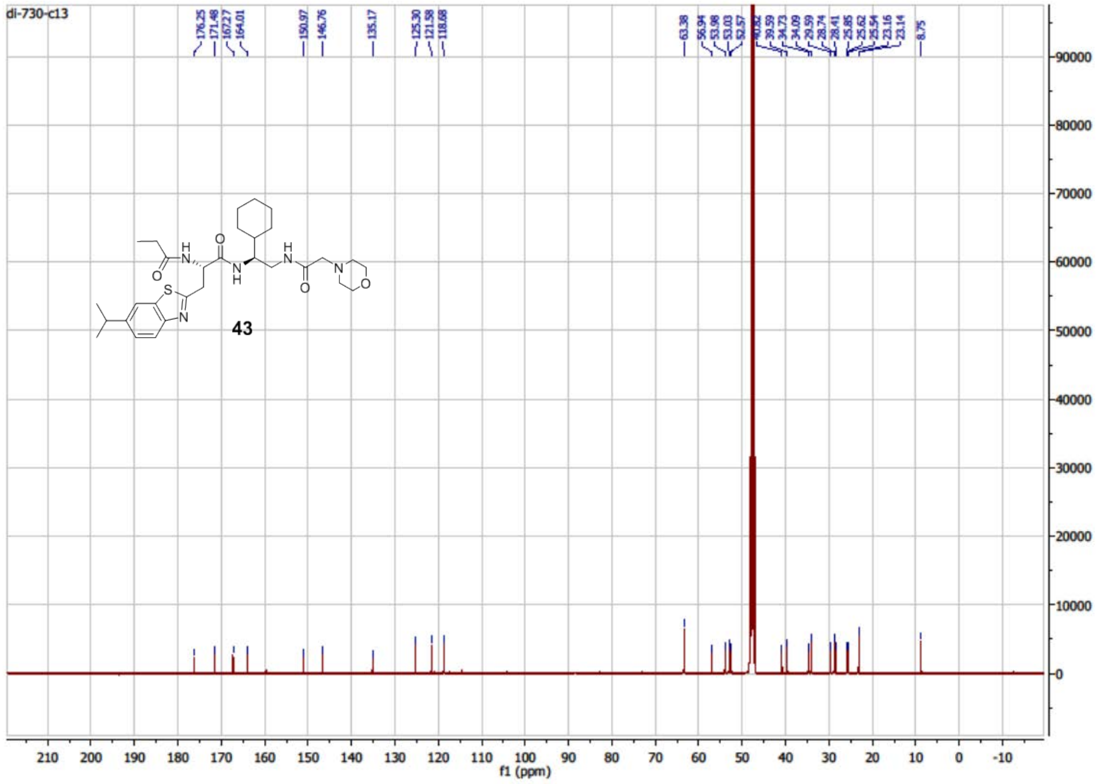

Supplementary Figure 43. ^{1}H NMR and ^{13}C NMR for DI-591 (Compound **44**).

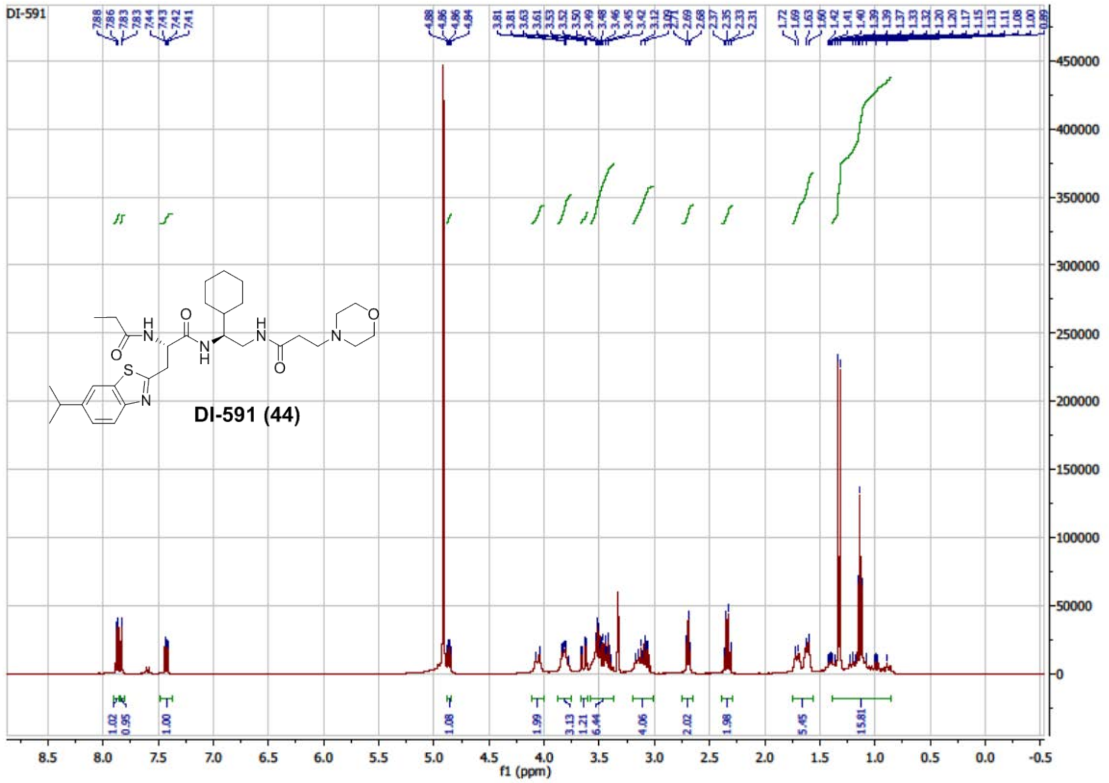

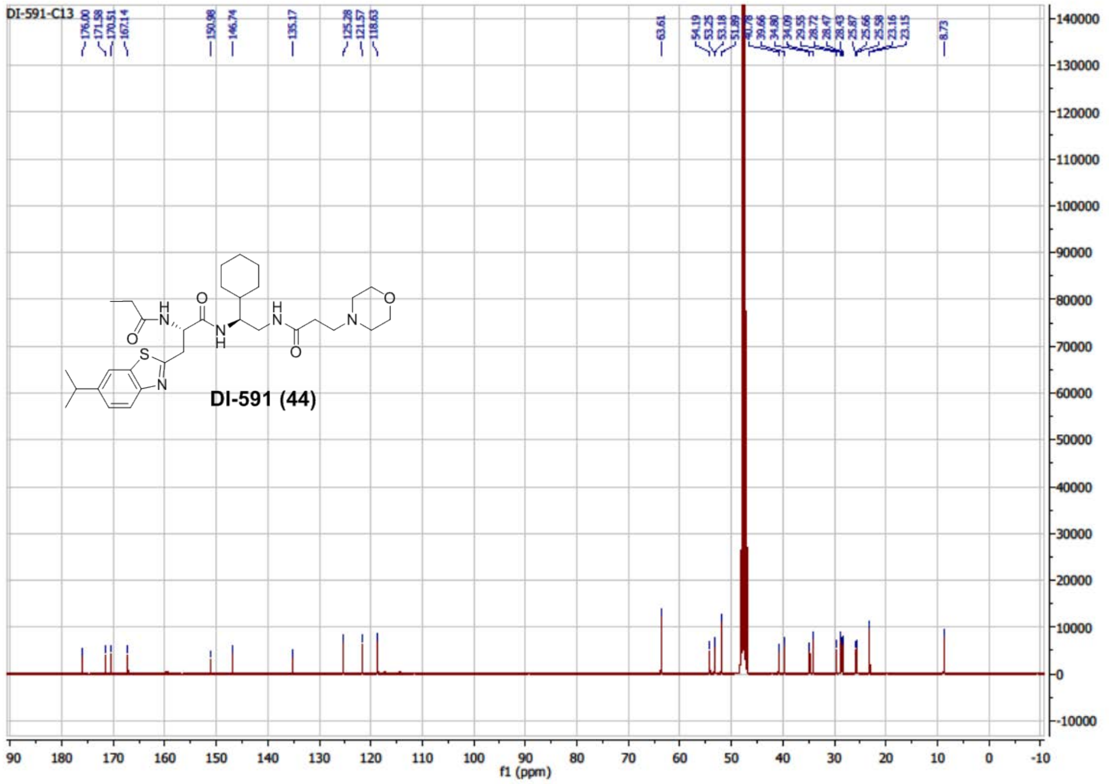

Supplementary Figure 44. ^{1}H NMR and ^{13}C NMR for DI-591DD (Compound **45**).

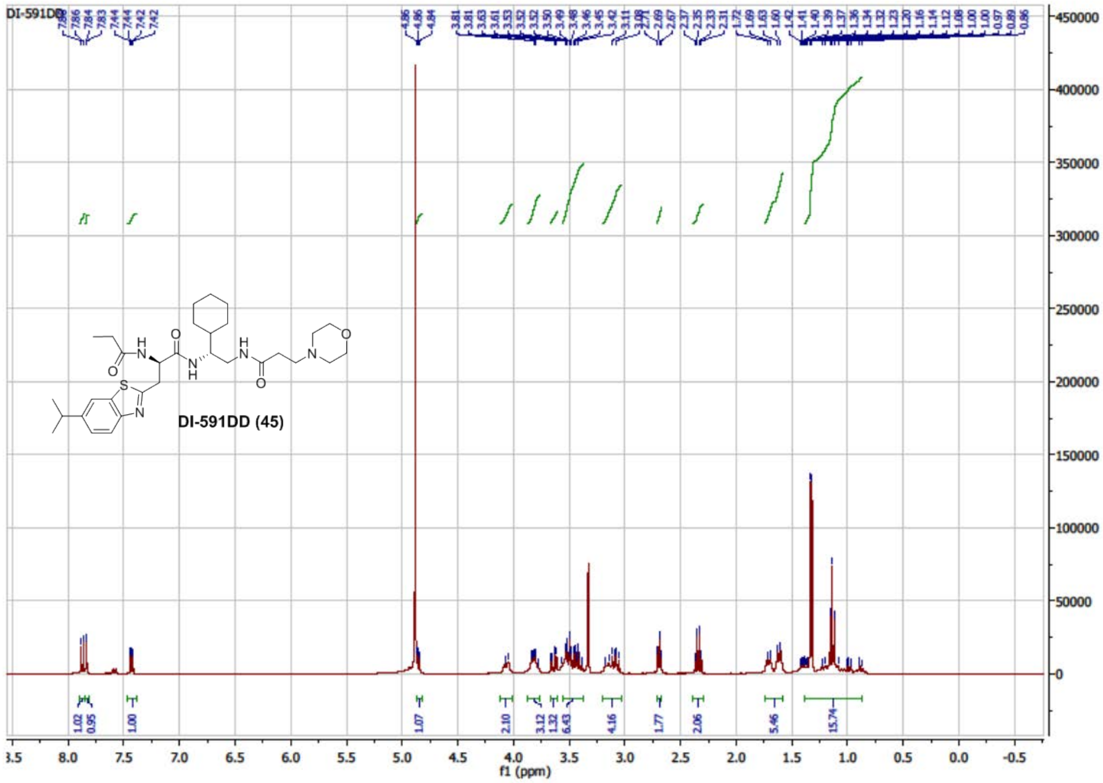

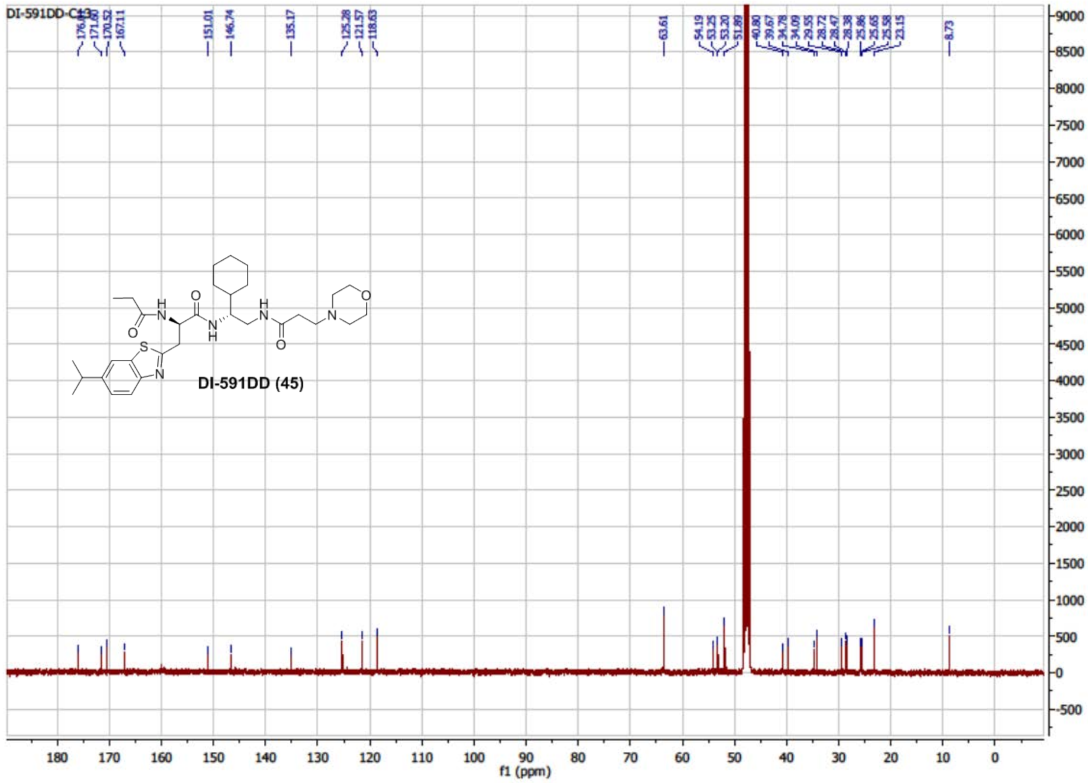

Supplementary Figure 45. ^{1}H NMR for Compound **46**.

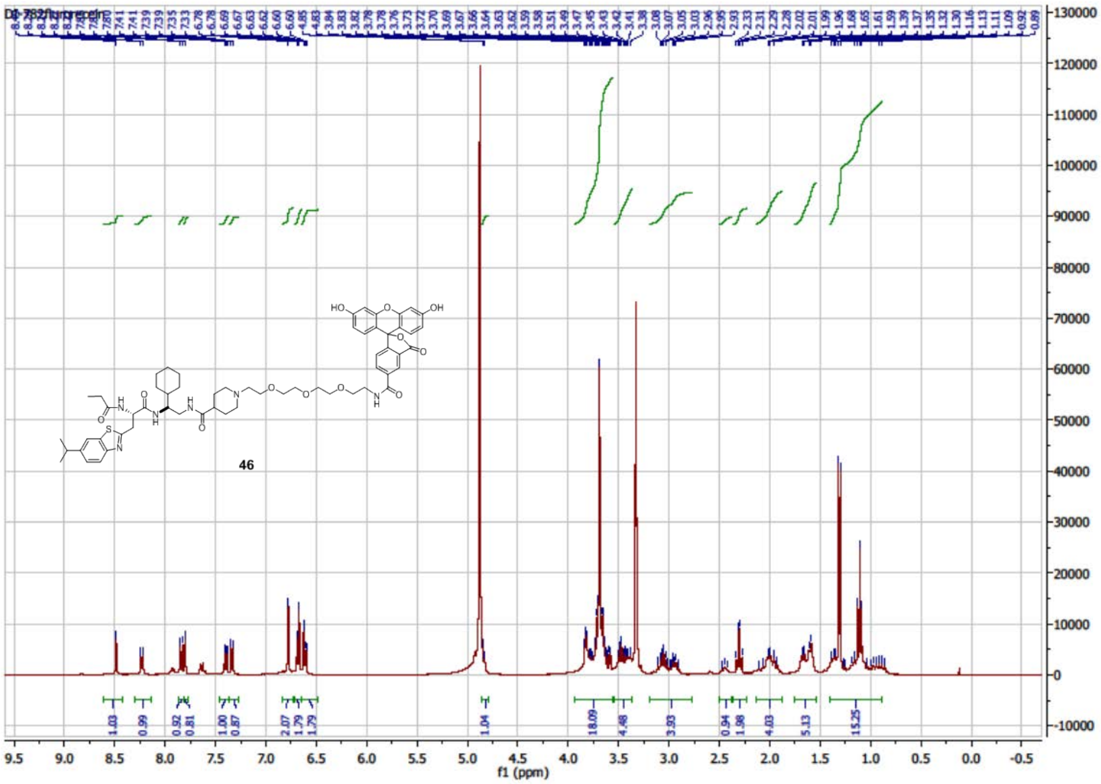

Supplementary Figure 46. ^{1}H NMR for Compound **47**.

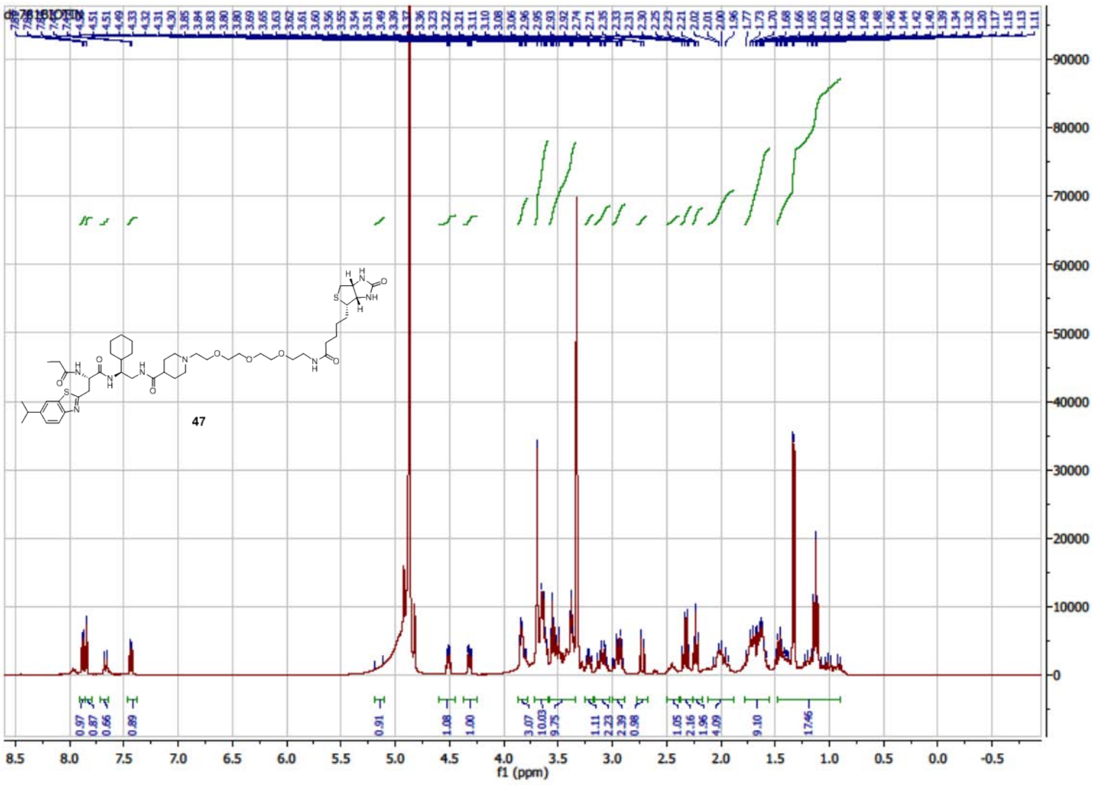

Supplementary Figure 47. ^{1}H NMR and ^{13}C NMR for Compound **49**.

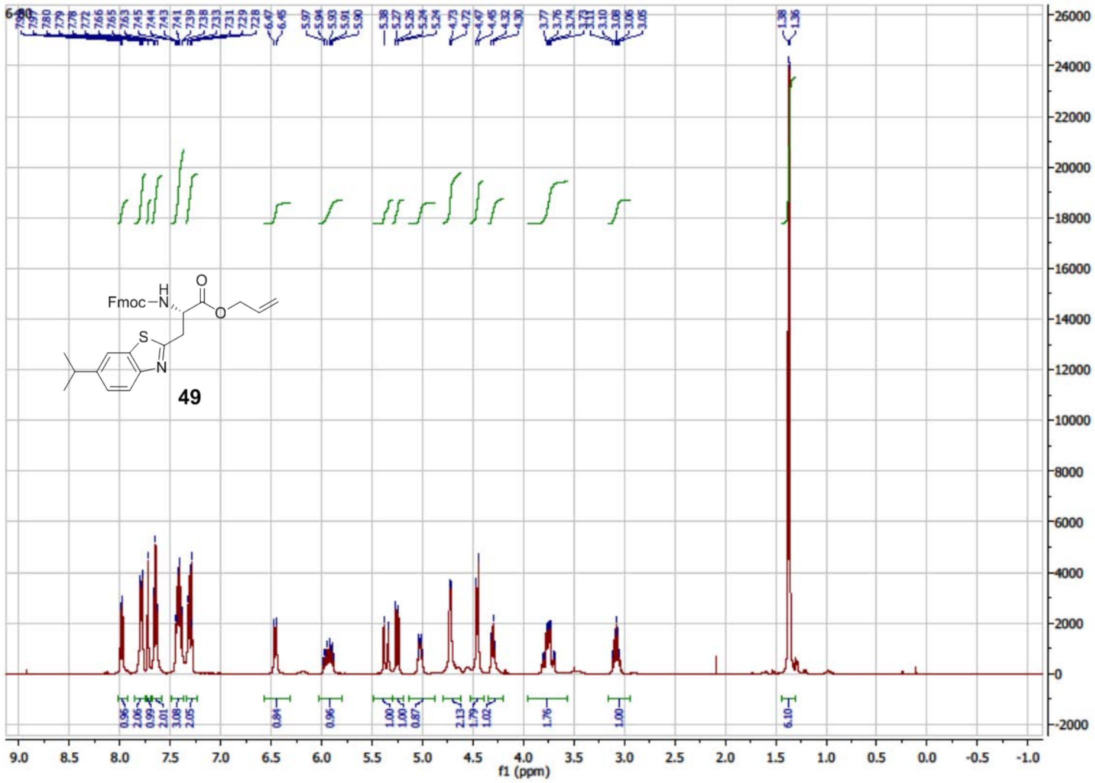

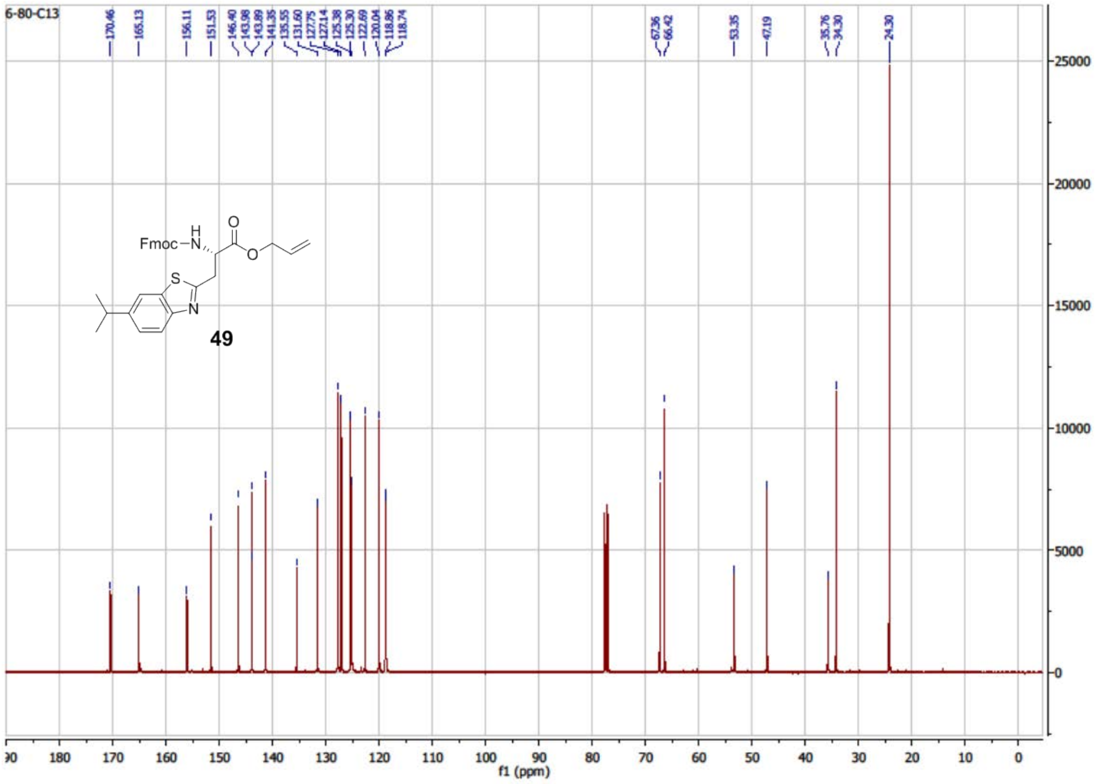

Supplementary Figure 48. ^{1}H NMR and ^{13}C NMR for Compound **50**.

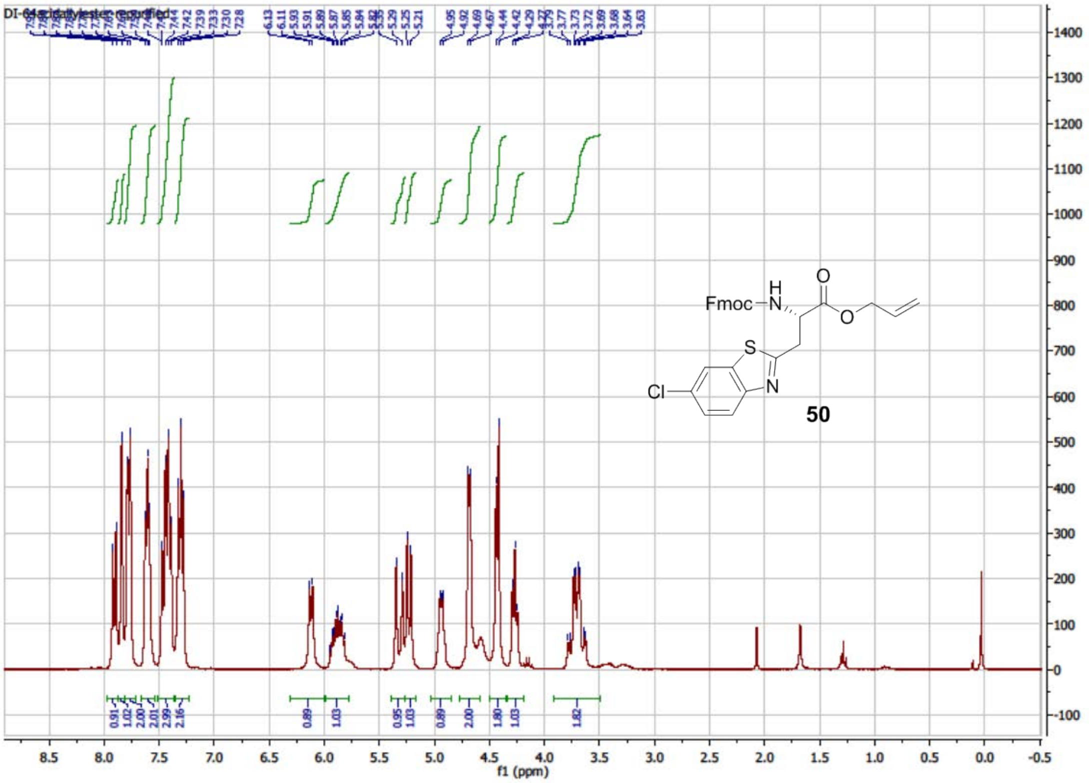

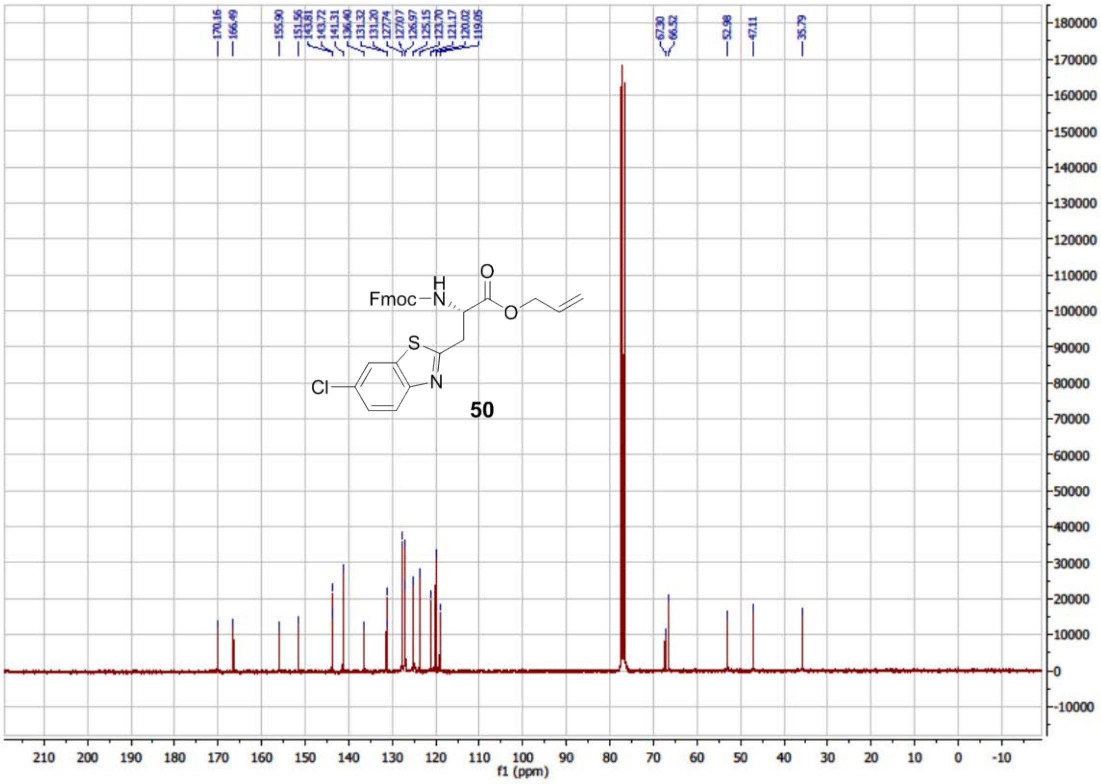

Supplementary Figure 49. ^{1}H NMR and ^{13}C NMR for Compound **51**.

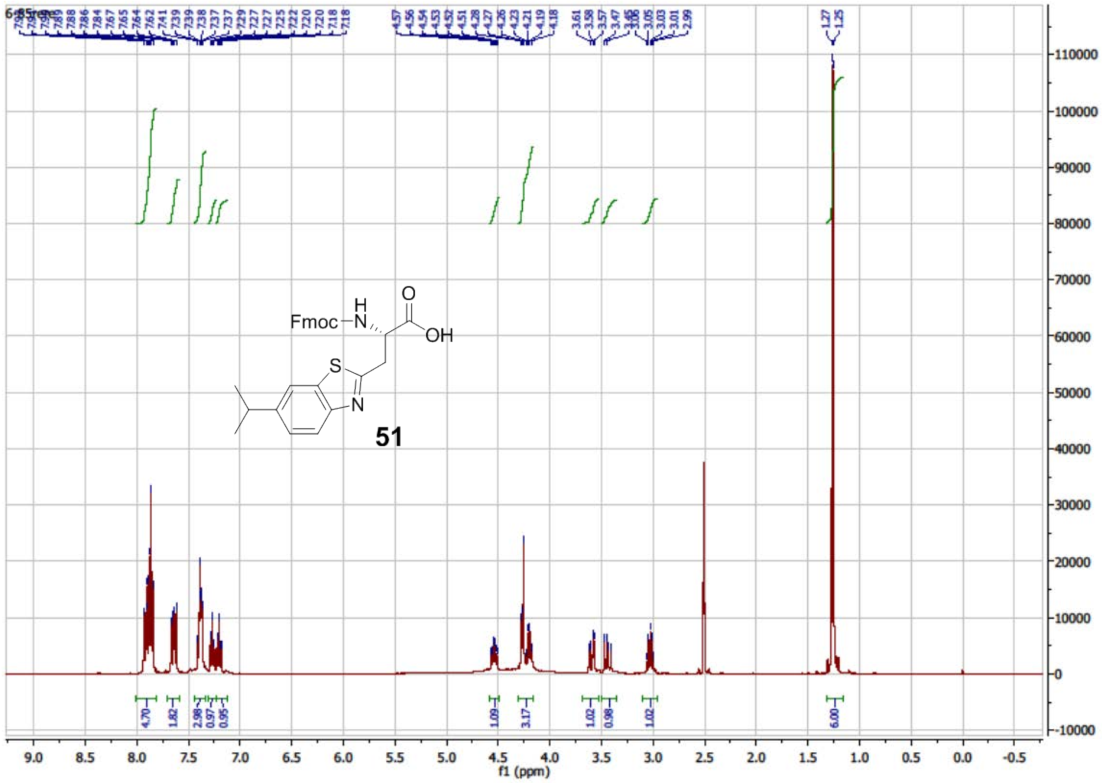

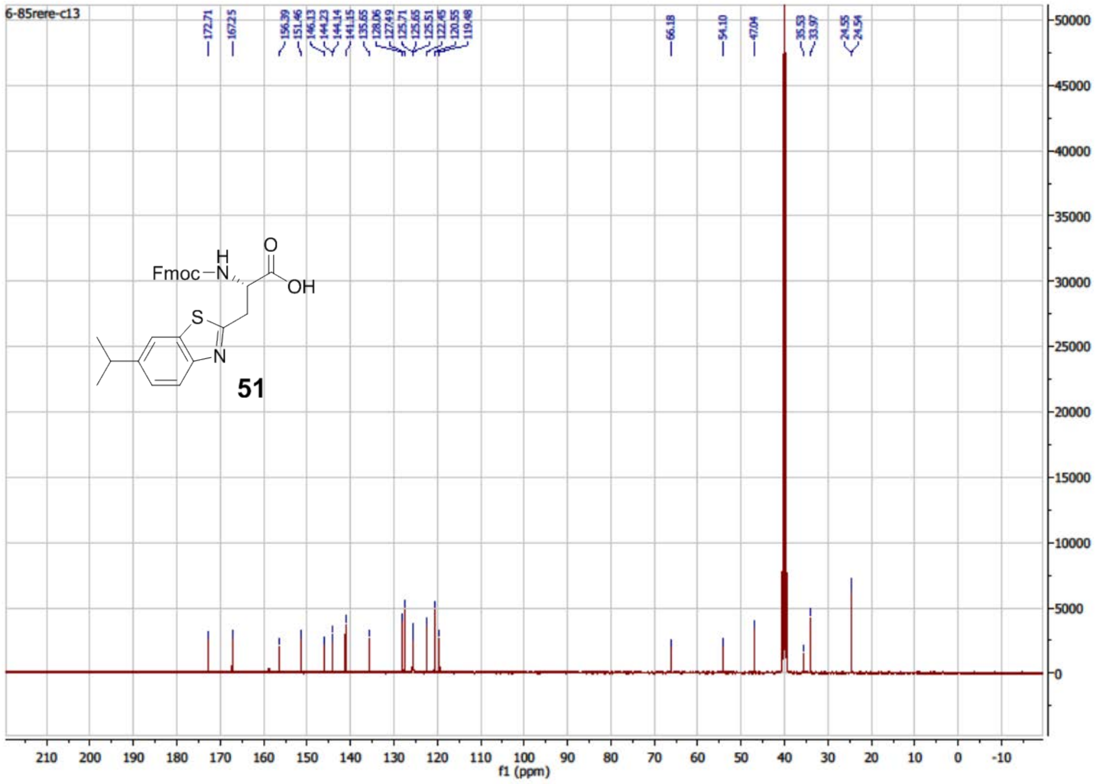

Supplementary Figure 50. ^{1}H NMR and ^{13}C NMR for Compound **52**.

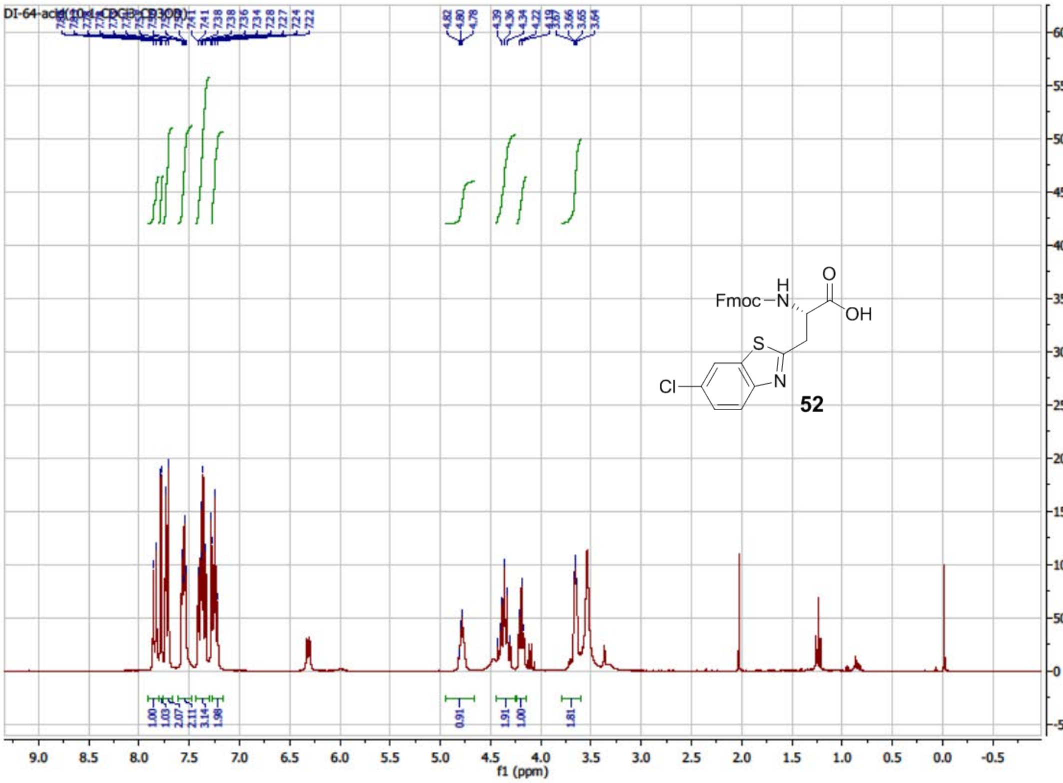

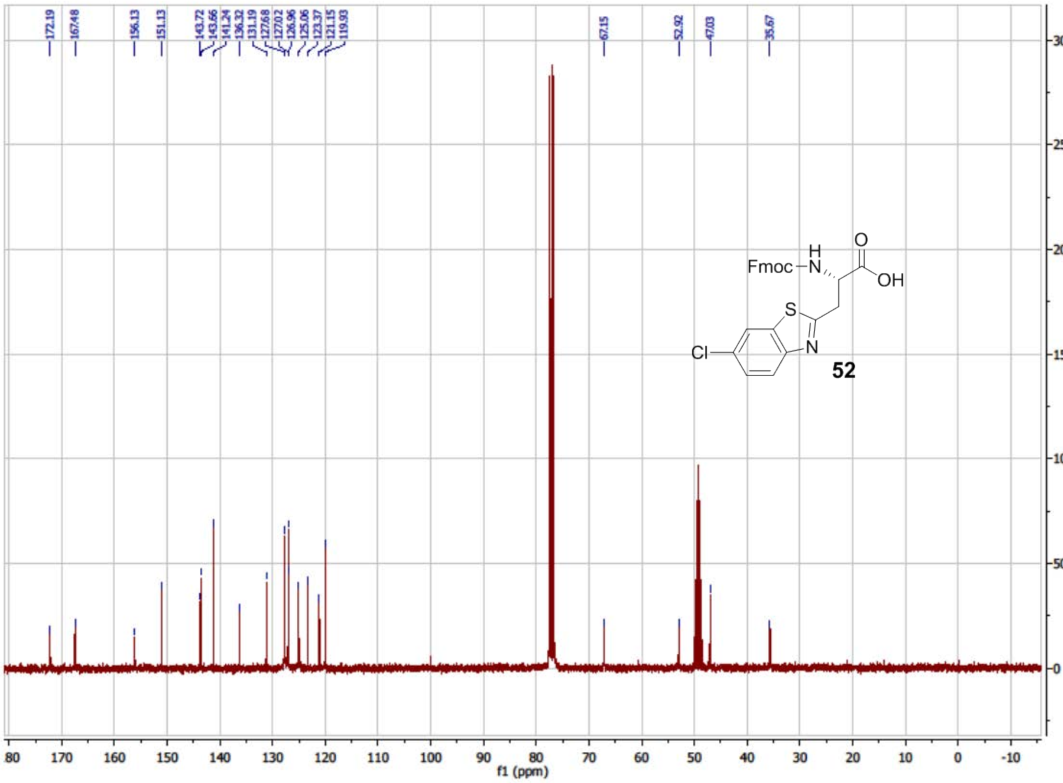

Supplementary Figure 51. ^{1}H NMR and ^{13}C NMR for Compound **53**.

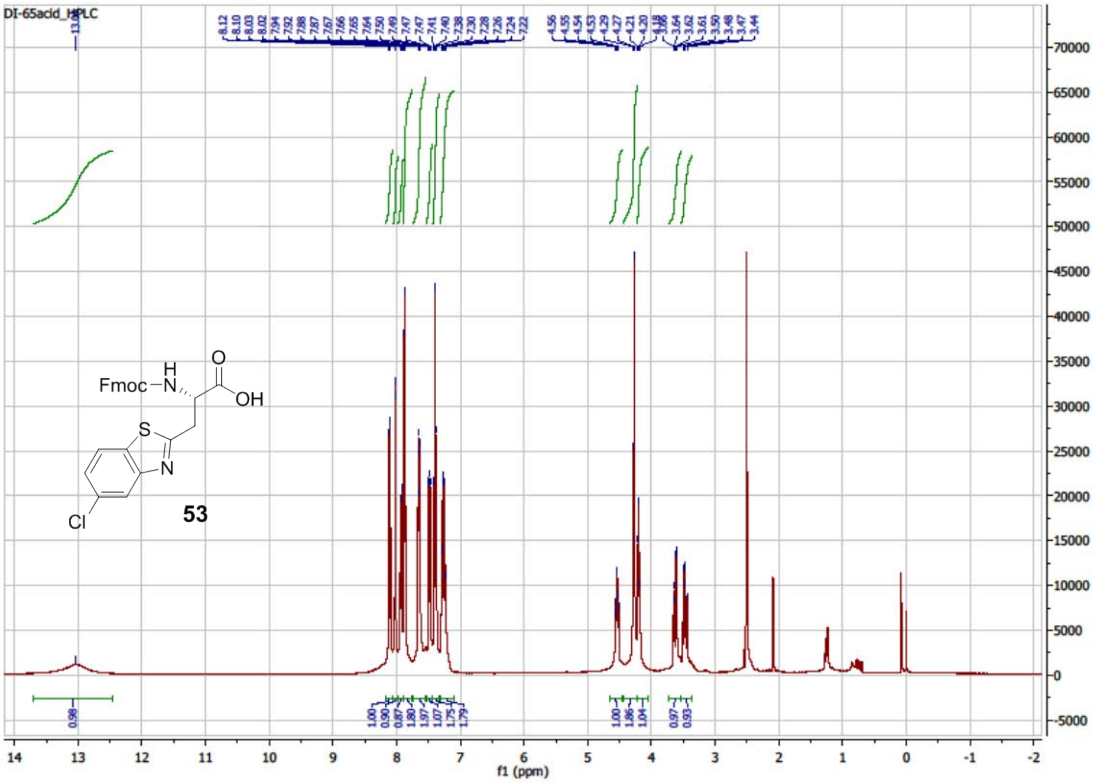

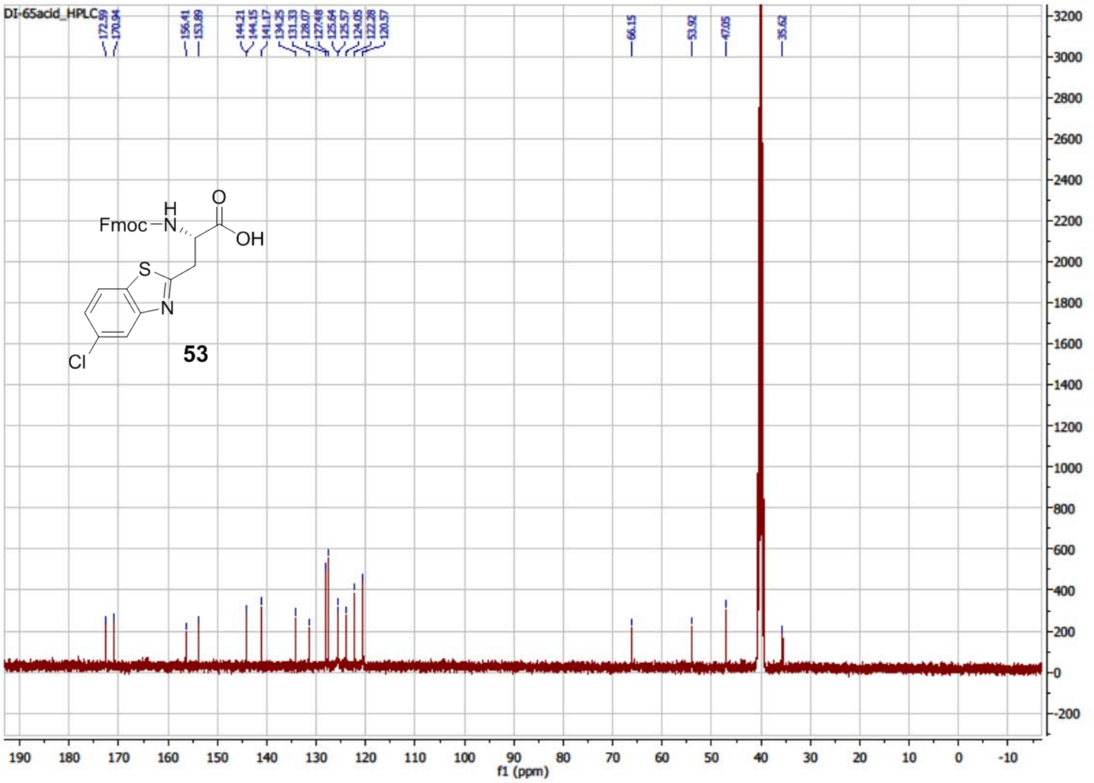

Supplementary Figure 52. ^{1}H NMR and ^{13}C NMR for Compound **54**.

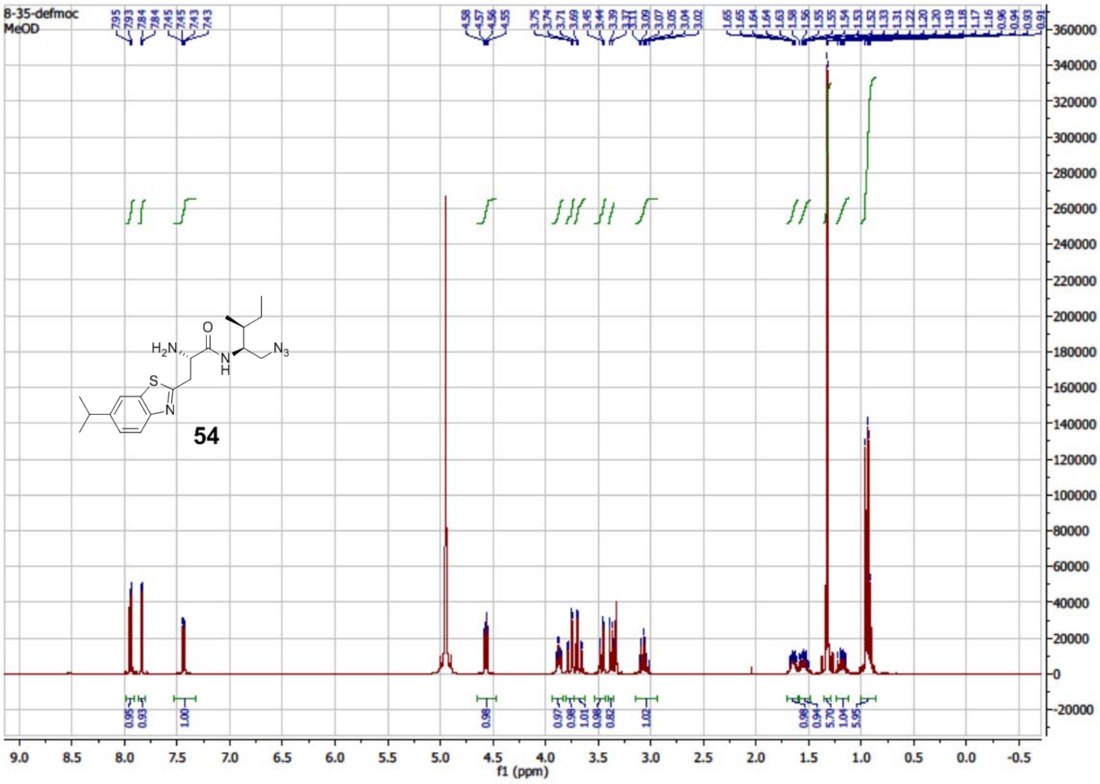

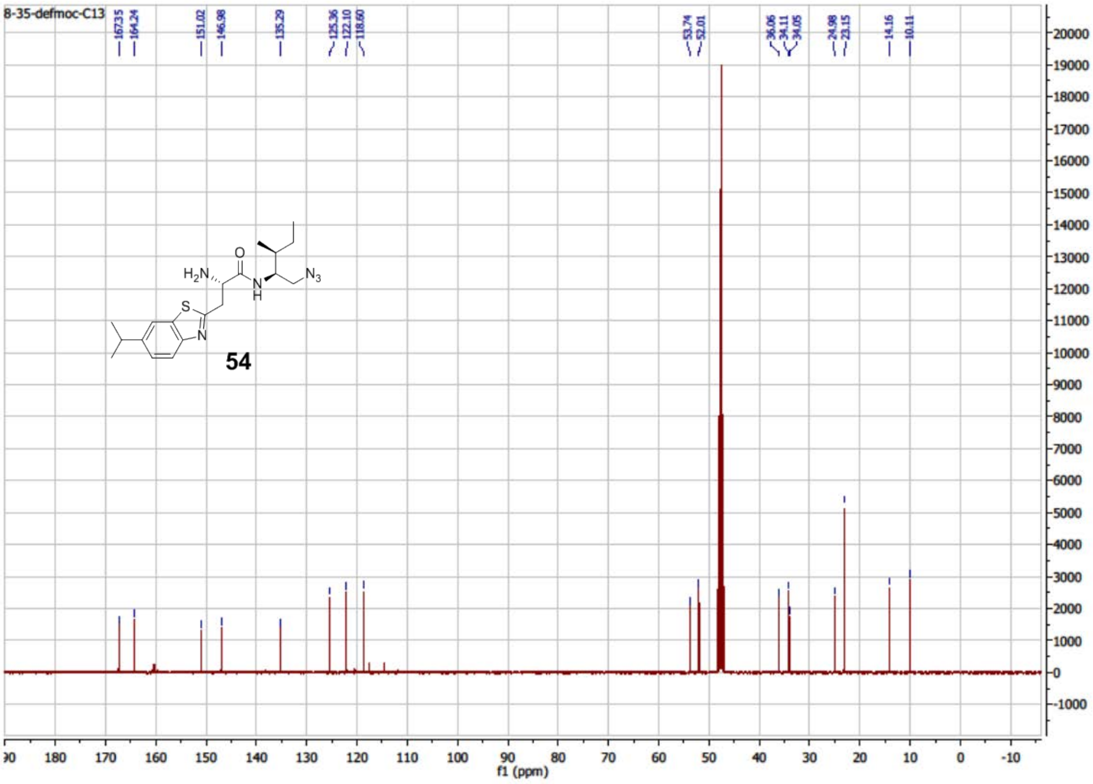

Supplementary Figure 53. ^{1}H NMR and ^{13}C NMR for Compound **55**.

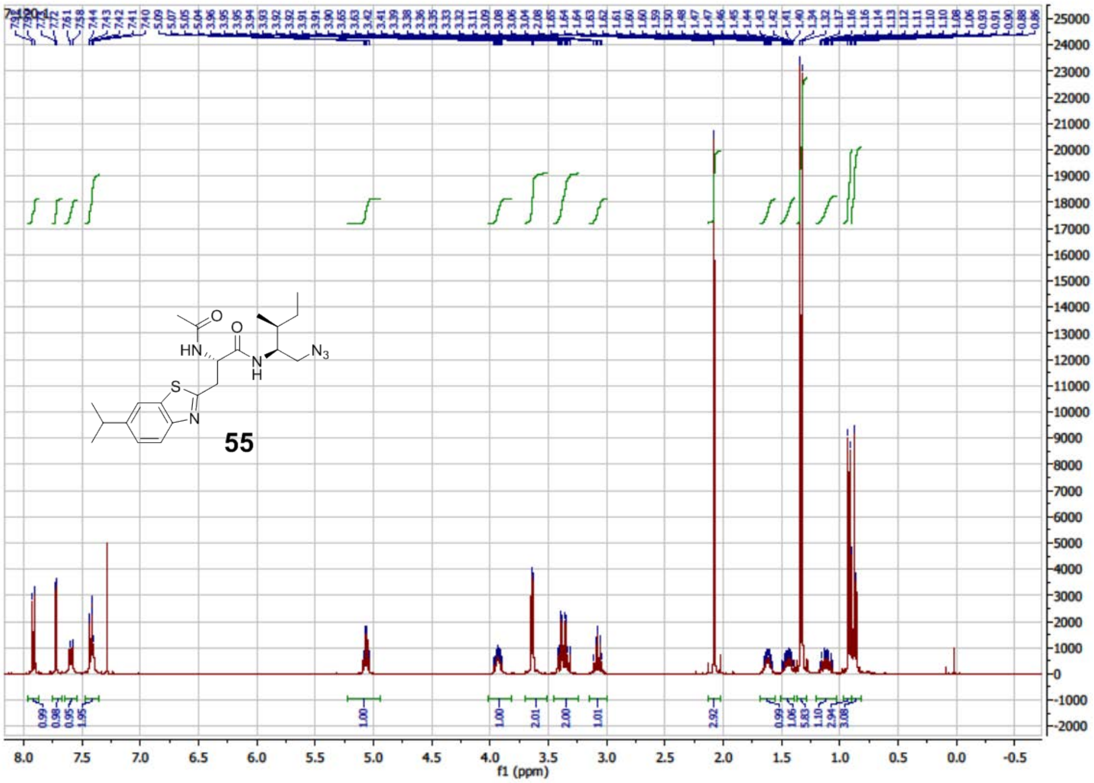

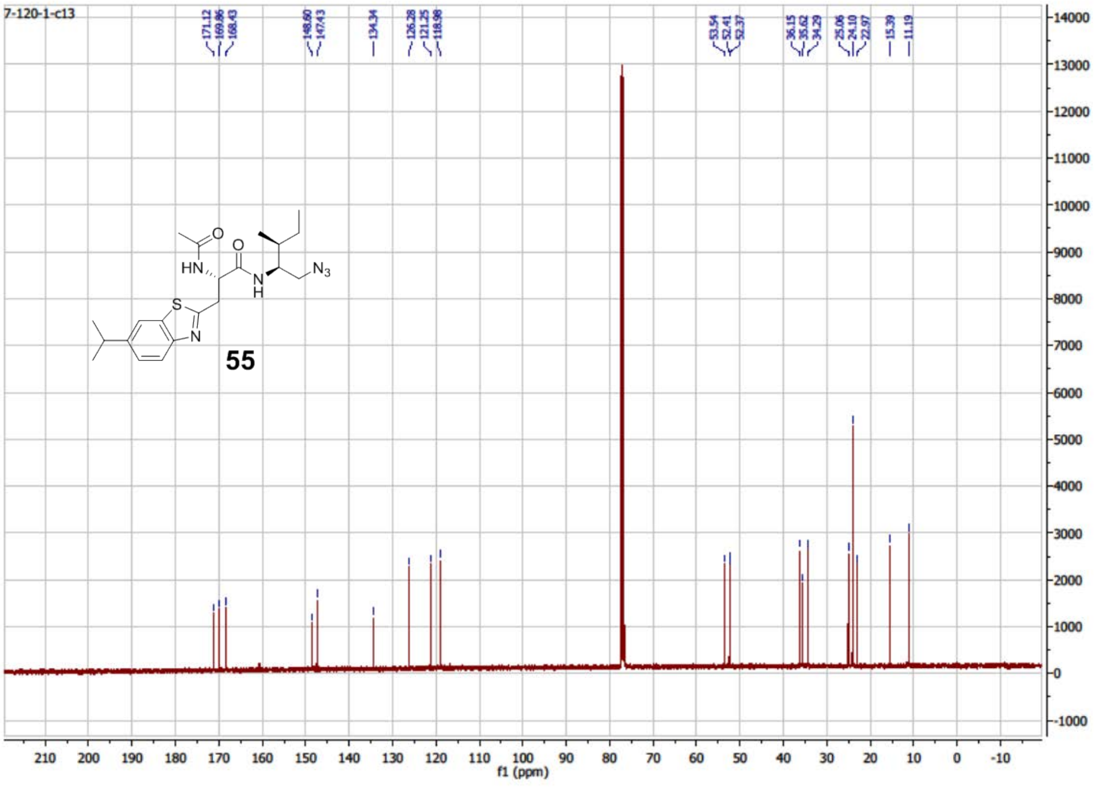

Supplementary Figure 54. ^{1}H NMR and ^{13}C NMR for Compound **56**.

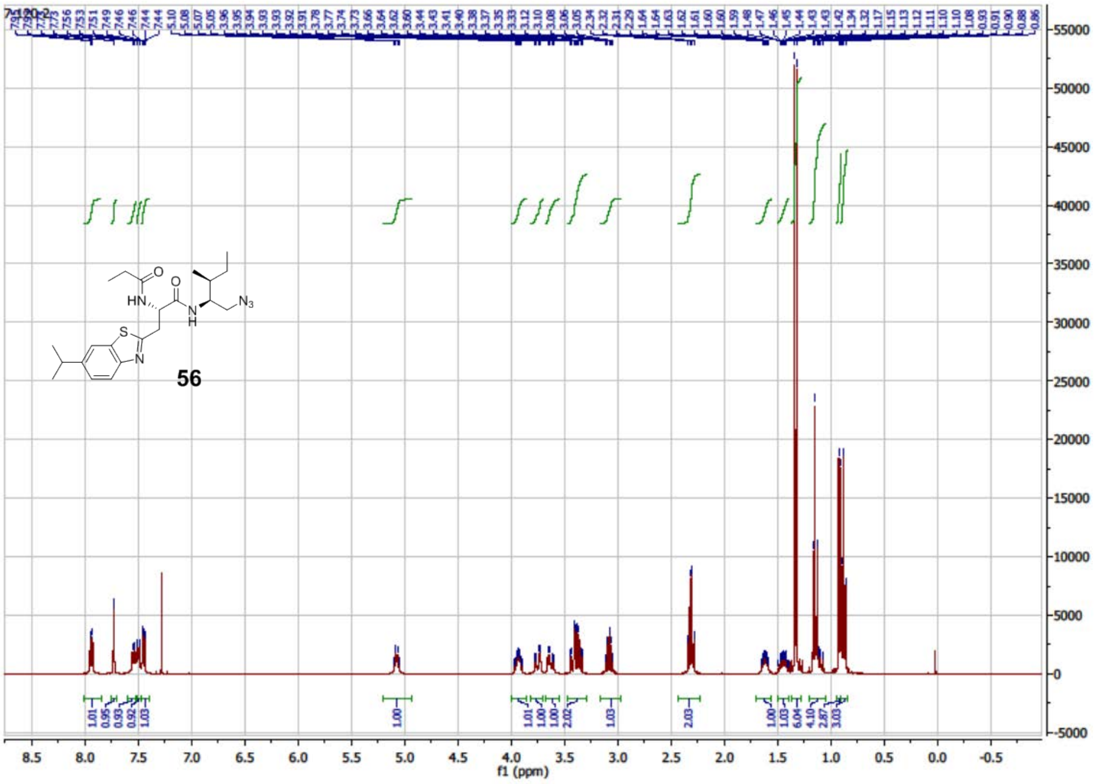

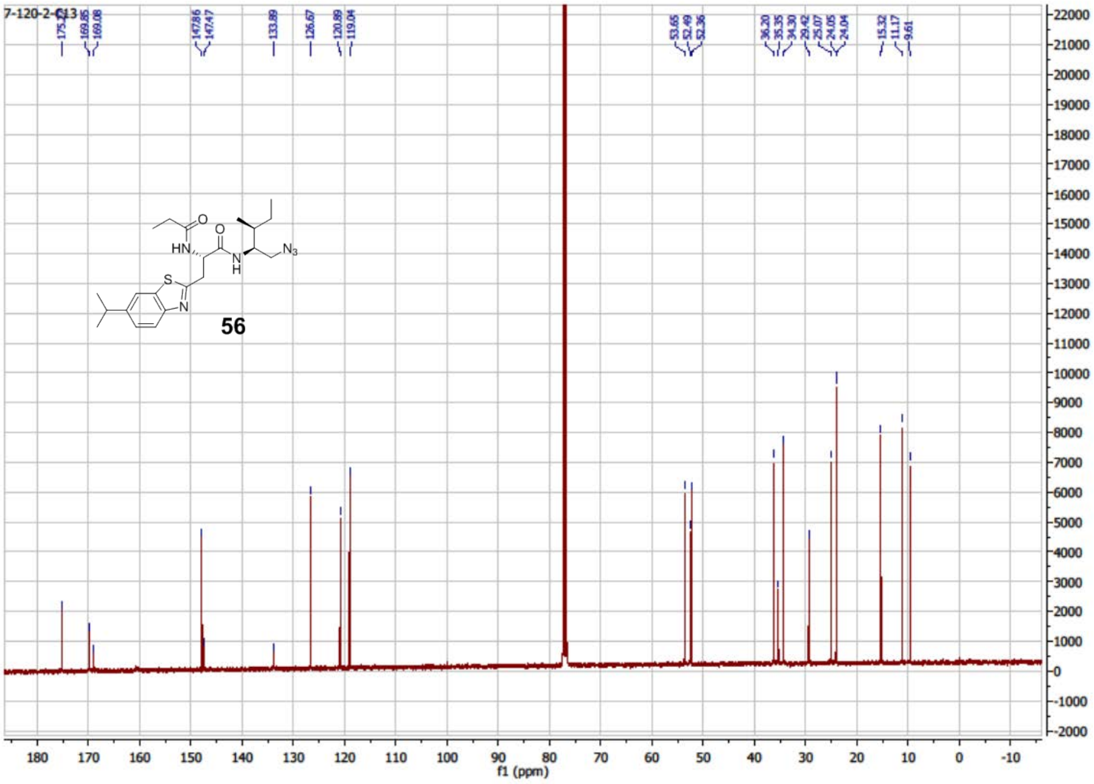

Supplementary Figure 55. ^{1}H NMR and ^{13}C NMR for Compound **57**.

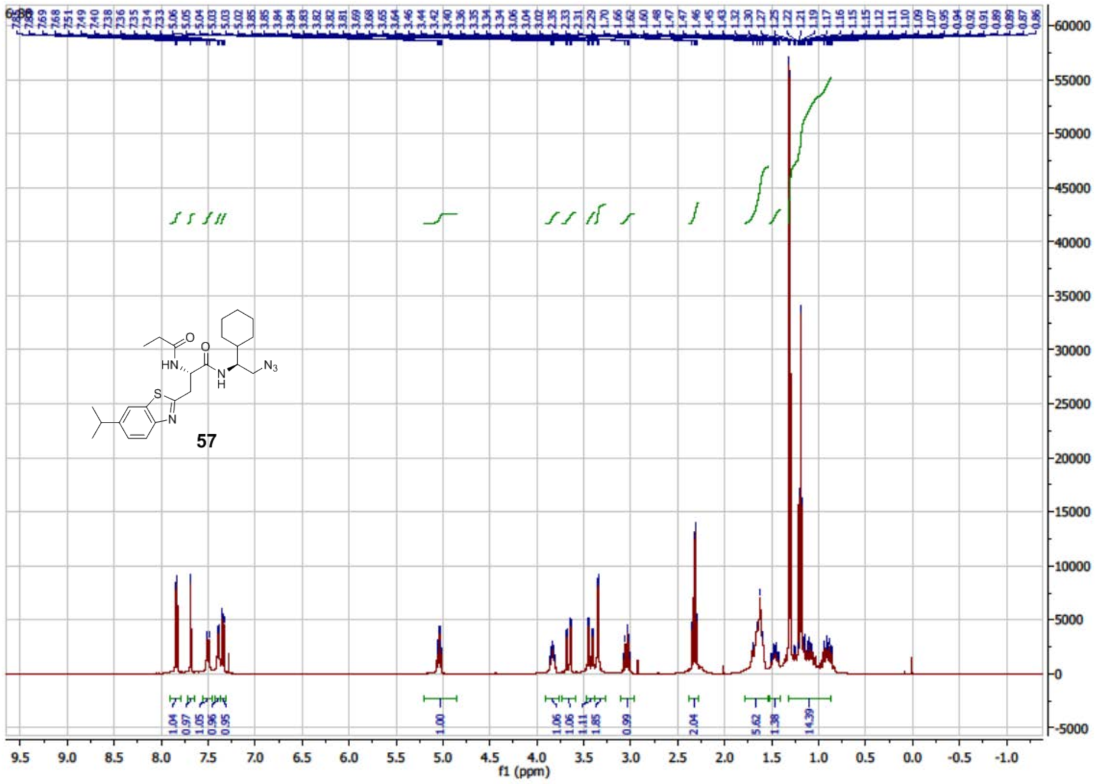

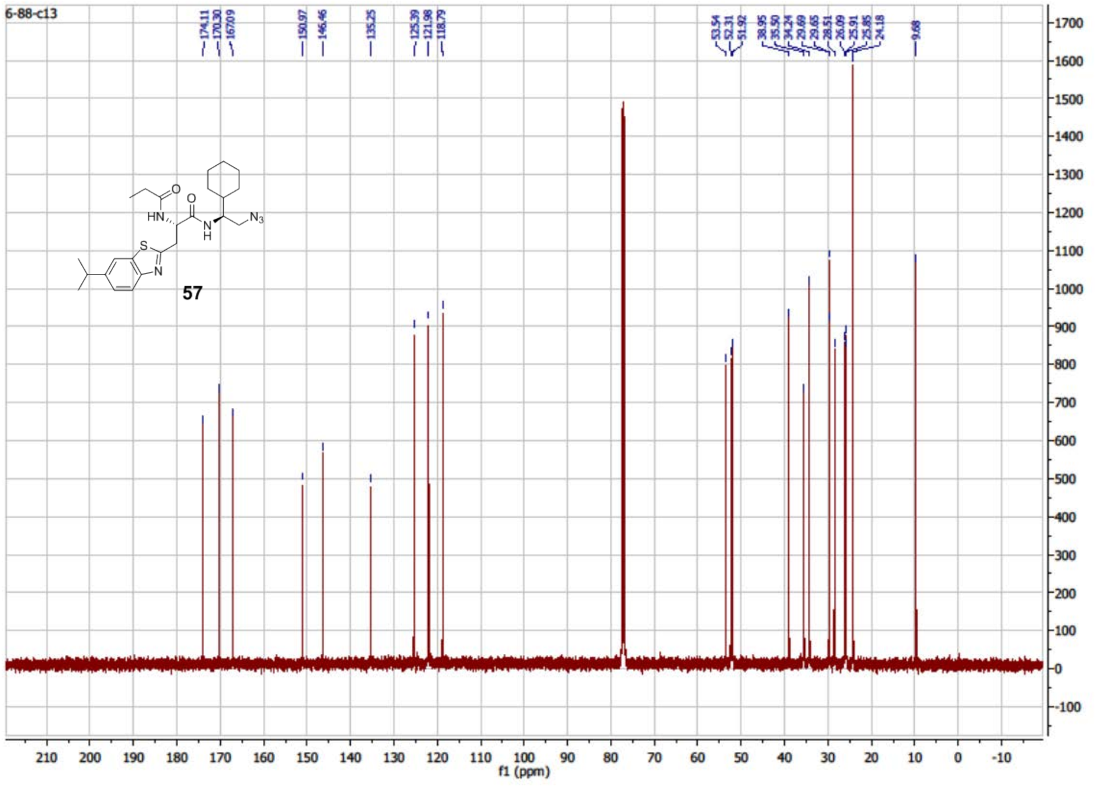

Supplementary Figure 56. ^{1}H NMR and ^{13}C NMR for Compound **60**.

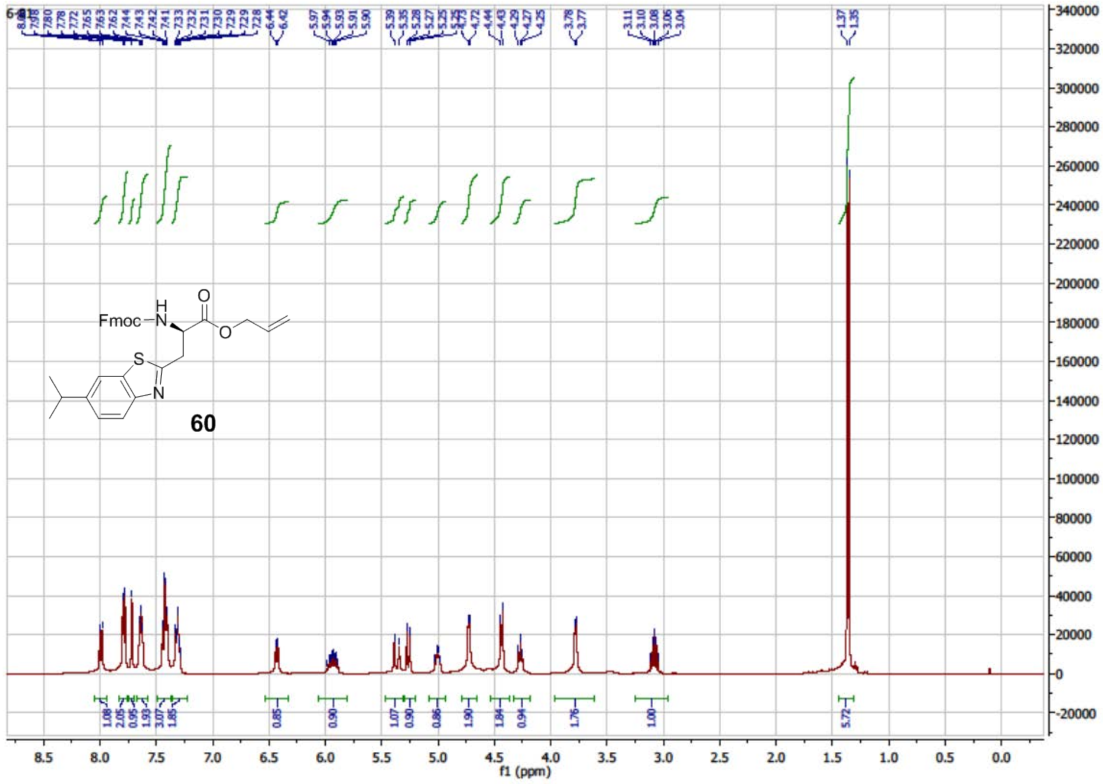

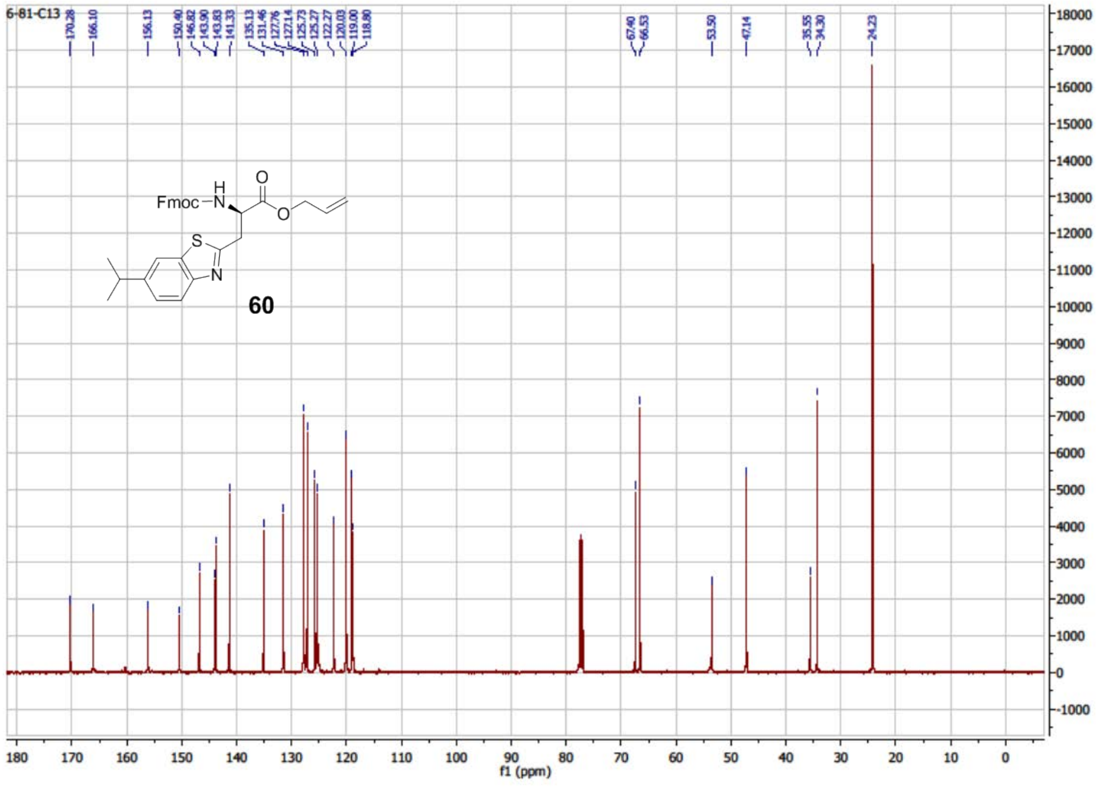

Supplementary Figure 57. ^{1}H NMR and ^{13}C NMR for Compound **61**.

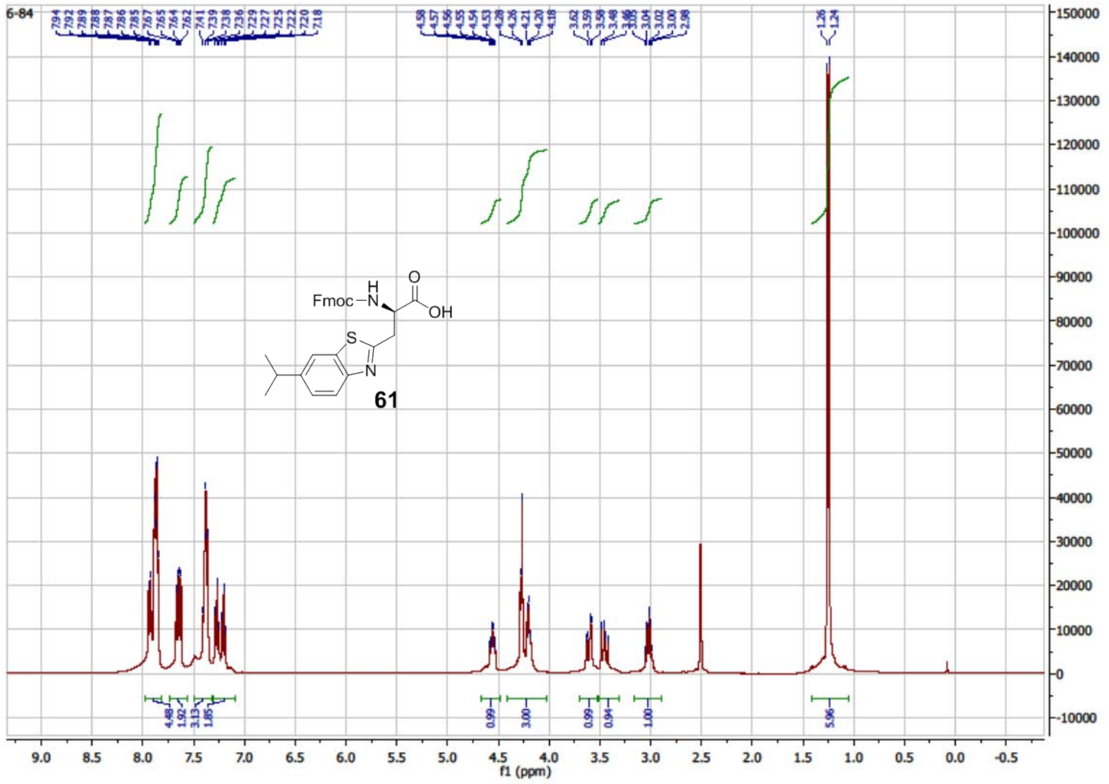

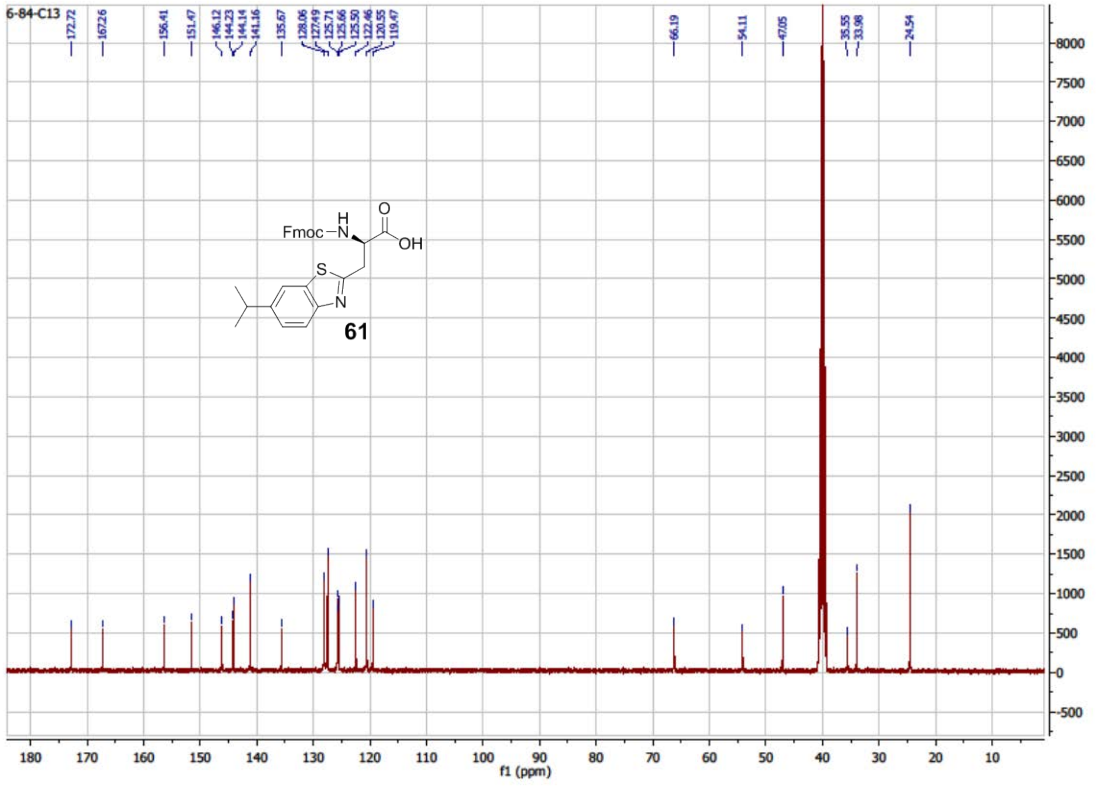

Supplementary Figure 58. ^{1}H NMR and ^{13}C NMR for Compound **63**.

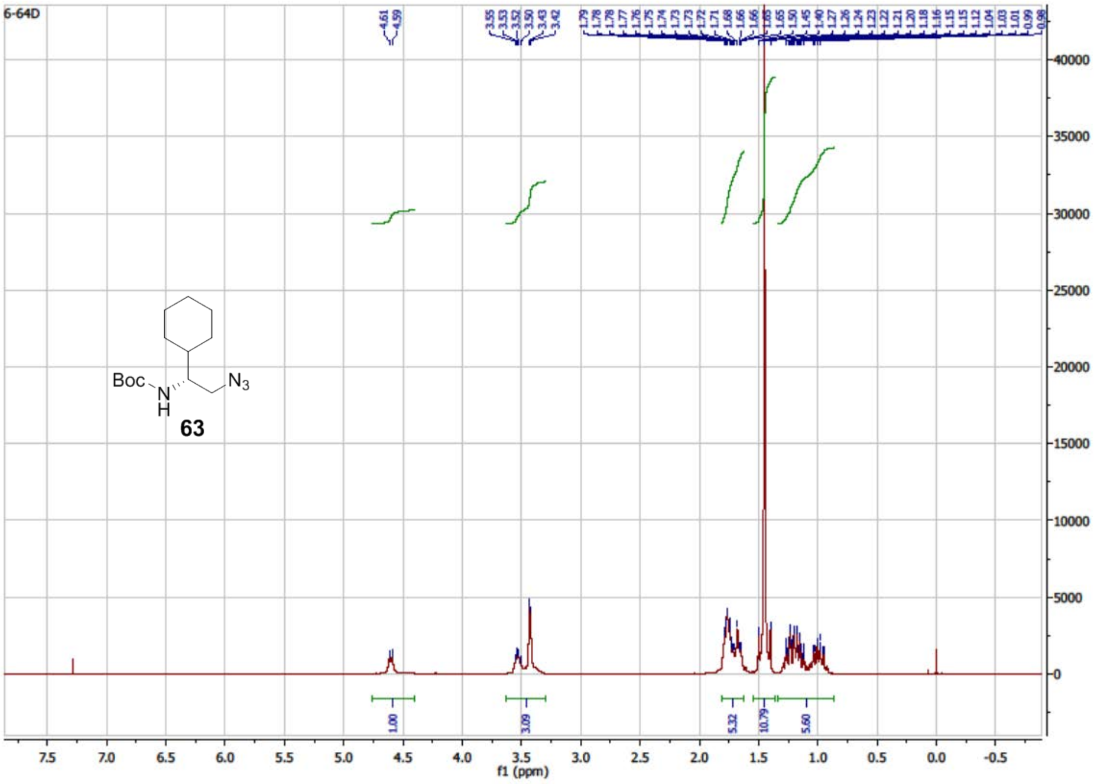

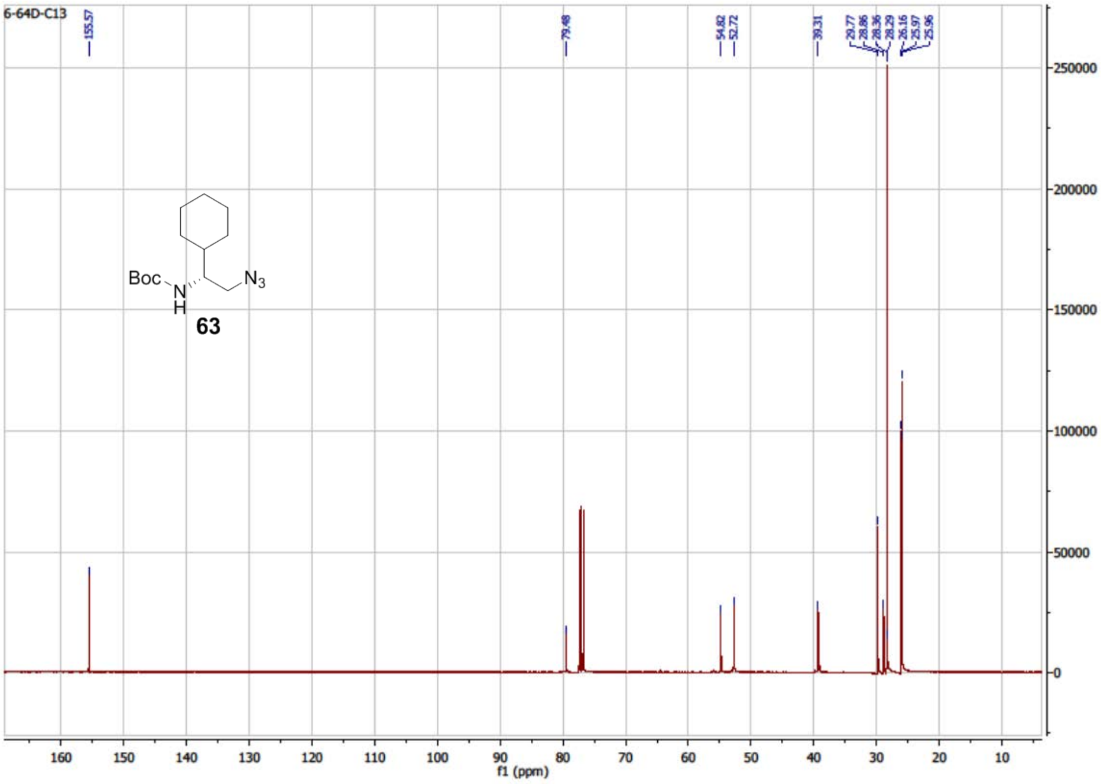

Supplementary Figure 59. ^{1}H NMR and ^{13}C NMR for Compound **64**.

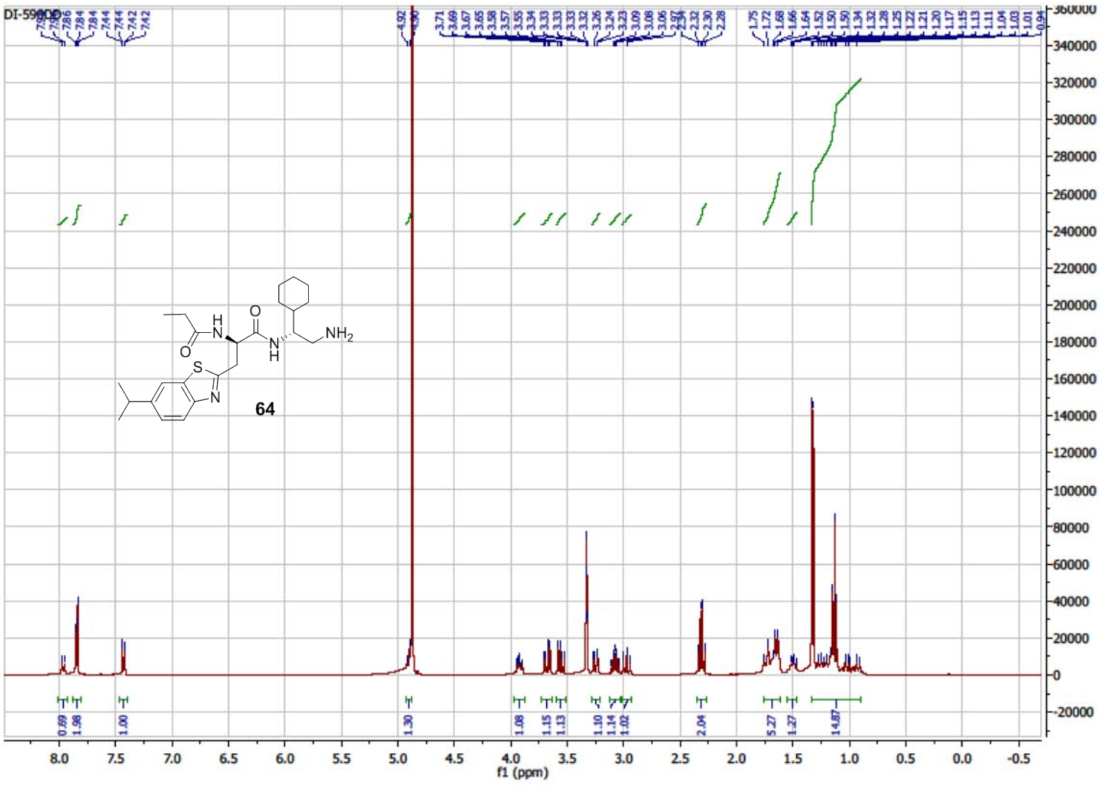

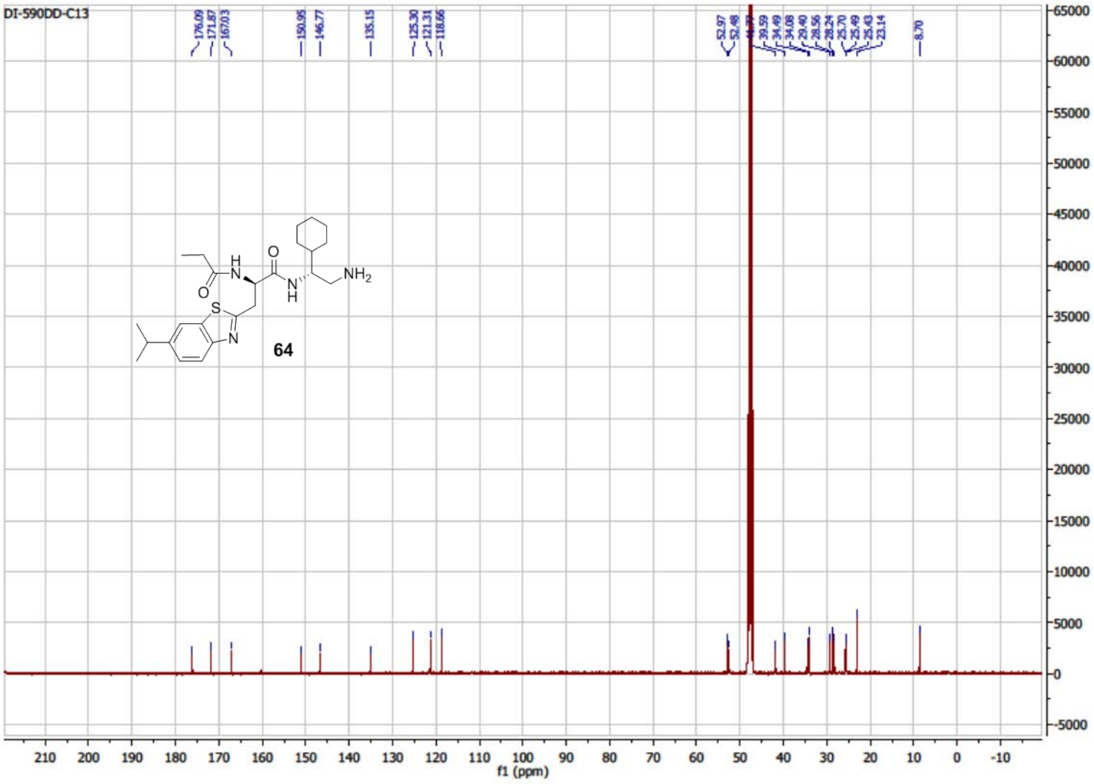

Supplementary Figure 60. ^{1}H NMR and ^{13}C NMR for Compound **65**.

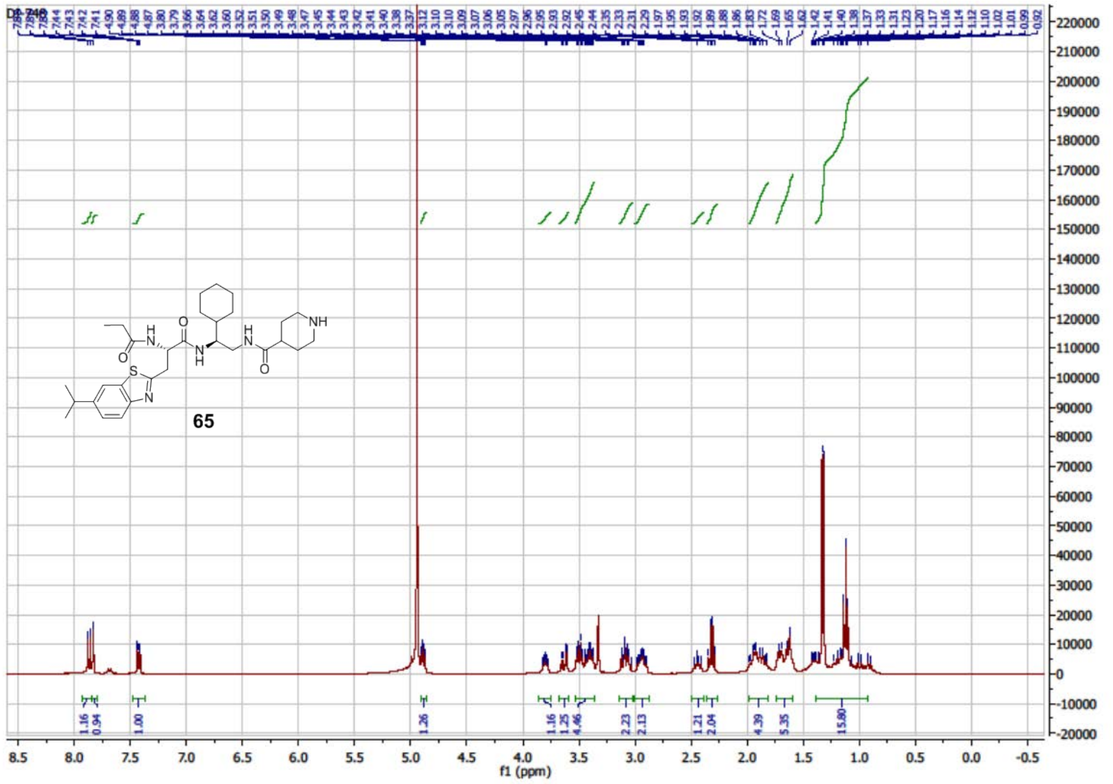

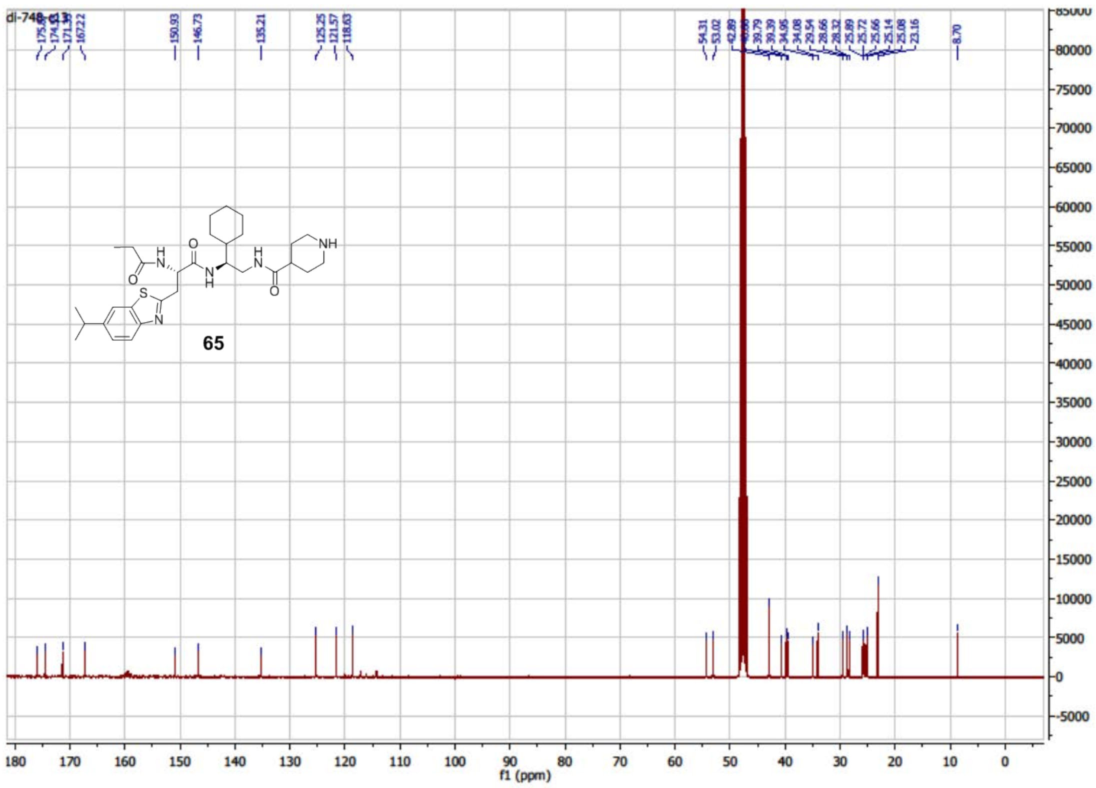

Supplementary Figure 61. ^{1}H NMR for Compound **66**.

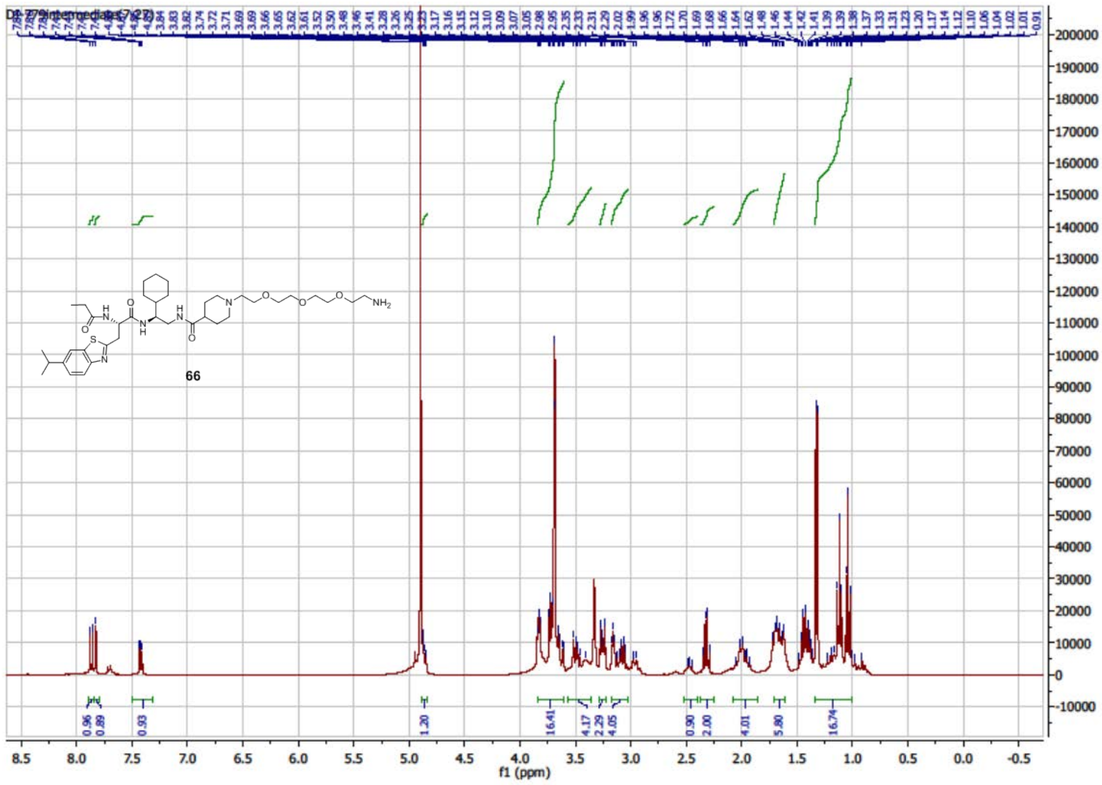

Supplementary Methods:

General Information

Unless otherwise noted, all reagents and solvents were used as supplied without further purification and the reactions were performed under nitrogen atmosphere in anhydrous solvents. The final products were purified by a reverse phase HPLC (RP-HPLC) with solvent A (0.1% of TFA in water) and solvent B (0.1% of TFA in CH_3CN) as eluents. The purity was determined by Waters ACQUITY UPLC and all the final compounds were > 95% pure. ^{1}H-NMR, ^{13}C-NMR spectra of the synthetic compounds were acquired at a proton frequency of 300 or 400 MHz and chemical shifts are reported in parts per million (ppm) relative to an internal standard. High resolution mass spectra (HRMS) were obtained from Agilent Q-TOF Electrospray mass spectrometer and low resolution mass spectra analyses were determined by Waters ACQUITY UPLC-MS or by a Thermo-Scientific LCQ Fleet mass spectrometer.

Synthesis of intermediates 51-53:

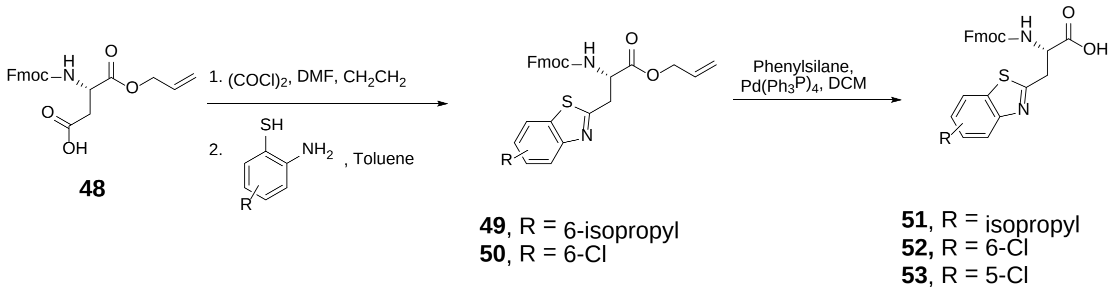

Compounds **49** and **50** were afforded by transforming the carboxylic acid of compound **48** to benzothiazoles. A reported method[1] was employed for forming the benzothiazole ring.

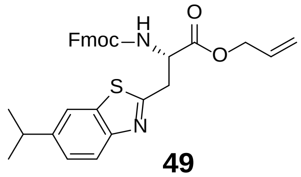

49

Allyl (S)-2-((((9H-fluoren-9-yl)methoxy)carbonyl)amino)-3-(6-isopropylbenzo[d]thiazol-2-yl)propanoate (49): To a solution of (S)-3-((((9H-fluoren-9-yl)methoxy)carbonyl)amino)-4-(allyloxy)-4-oxobutanoic acid (**48**, 5 g, 12.6 mmol) in CH_2Cl_2 (300 mL), oxalyl chloride (3.3 mL, 38.0 mmol) and catalytic amounts of DMF were added at 0^oC. The reaction mixture was concentrated after being stirred for 0.5 h. The residue was suspended in toluene (250 mL) and treated with 2-amino-5-isopropylbenzenethiol (2.1 g, 12.6 mmol). The resultant mixture was stirred overnight at room temperature. The solution was diluted with EtOAc and washed with saturated sodium bicarbonate, 1.0 M HCl, brine and dried over sodium sulfate. The solvent was evaporated and the crude product was purified by flash chromatography on silica gel to afford intermediate **49** (3.5 g, 53%). ^1H NMR (400 MHz, $CDCl_3$) δ 7.98 (d, *J* = 8.4 Hz, 1H), 7.86 – 7.75 (m, 2H), 7.72 (s, 1H), 7.65 (t, *J* = 7.1 Hz, 2H), 7.49 – 7.36 (m, 3H), 7.33-7.27 (m, 2H), 6.46 (d, *J* = 8.5 Hz, 1H), 5.98-5.88 (m, 1H), 5.36 (d, *J* = 17.2 Hz, 1H), 5.25 (dd, *J* = 10.4, 0.8 Hz, 1H), 5.02 (dt, *J* = 8.5, 5.3 Hz, 1H), 4.72 (d, *J* = 4.9 Hz, 2H), 4.46 (d, *J* = 7.3 Hz, 2H), 4.30 (t, *J* = 7.3 Hz, 1H), 3.75 (qd, *J* = 15.7, 5.3 Hz, 2H), 3.08 (dt, *J* = 13.7, 6.9 Hz, 1H), 1.37 (d, *J* = 6.9 Hz, 6H). ^{13}C NMR (101 MHz, $CDCl_3$) δ 170.46, 165.13, 156.11, 151.53, 146.40, 143.98, 143.89, 141.35, 135.55, 131.60, 127.75, 127.14, 125.38, 125.30, 122.69, 120.04, 118.86, 118.74, 67.36, 66.42, 53.35, 47.19, 35.76, 34.30, 24.30. UPLC-MS (ESI-MS) *m/z*: calculated for $C_{31}H_{31}N_2O_4S^+$ 527.20, found 527.26 $[M+H]^+$.

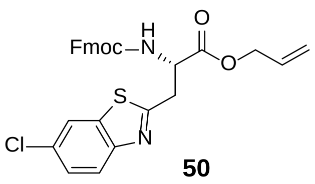

50

Allyl (S)-2-((((9H-fluoren-9-yl)methoxy)carbonyl)amino)-3-(6-chlorobenzo[d]thiazol-2-yl)propanoate (50): Intermediate **50** was prepared in 46% yield by a similar procedure as that for **49**. ^{1}H NMR (300 MHz, $CDCl_3$) δ 7.91 (d, *J* = 8.7 Hz, 1H), 7.84 (d, *J* = 1.4 Hz, 1H), 7.78 (d, *J* = 7.3 Hz, 2H), 7.67 – 7.54 (m, 2H), 7.52 – 7.37 (m, 3H), 7.36 – 7.22 (m, 2H), 6.12 (d, *J* = 8.1 Hz, 1H), 5.95-5.82(m, 1H), 5.32 (d, *J* = 17.3 Hz, 1H), 5.23 (d, *J* = 10.3 Hz, 1H), 5.03 – 4.85 (m, 1H), 4.68 (d, *J* = 5.1 Hz, 2H), 4.43 (d, *J* = 7.1 Hz, 2H), 4.27 (t, *J* = 7.0 Hz, 1H), 3.71 (qd, *J* = 15.8, 4.9 Hz, 2H). ^{13}C NMR (75 MHz, $CDCl_3$) δ 170.16, 166.49, 155.90, 151.56, 143.72, 141.31, 136.40, 131.32, 131.20, 127.74, 127.07, 126.97, 125.15, 123.70, 121.17, 120.02, 119.05, 67.30, 66.52, 52.98, 47.11, 35.79.

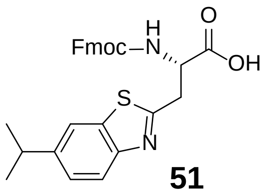

51

(S)-2-((((9H-fluoren-9-yl)methoxy)carbonyl)amino)-3-(6-isopropylbenzo[d]thiazol-2-yl)propanoic acid (51): Phenylsilane (1.9 g, 17.1 mmol) was added to a solution of **49** (3.0 g, 5.7 mmol) and Tetrakis(triphenylphosphine)palladium(0) (658 mg, 0.57mmol) in DCM. The resultant solution was stirred 1 h before being concentrated. The residue was purified by flash chromatography on silica gel to afford **51** (2.24 g, 81%). ^{1}H NMR (400 MHz, DMSO) δ 8.01 – 7.82 (m, 5H), 7.64 (dd, *J* = 11.7, 7.6 Hz, 2H), 7.41-7.37 (m, 3H), 7.29-7.25 (m, 1H), 7.23 – 7.13 (m, 1H), 4.57-4.51 (m, 1H), 4.30 – 4.16 (m, 3H), 3.60 (dd, *J* = 15.1, 4.6 Hz, 1H), 3.44 (dd, *J* = 15.0, 9.9 Hz, 1H), 3.03 (dt, *J* = 13.7, 6.8 Hz, 1H), 1.26 (d, *J* = 6.9 Hz, 6H). ^{13}C NMR (101 MHz, DMSO) δ 172.71, 167.25, 156.39, 151.46, 146.13, 144.23, 144.14, 141.15, 135.65, 128.06, 127.49, 125.71, 125.65, 125.51, 122.45, 120.55, 119.48, 66.18, 54.10, 47.04, 35.53, 33.97, 24.55, 24.54. UPLC-MS (ESI-MS) *m/z*: calculated for $C_{28}H_{27}N_2O_4S^{+}$ 487.17, found 487.19 $[M+H]^{+}$.

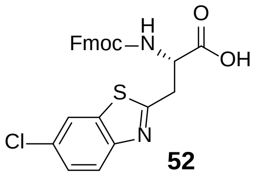

52

(S)-2-((((9H-fluoren-9-yl)methoxy)carbonyl)amino)-3-(6-chlorobenzo[d]thiazol-2-yl)propanoic acid (52): Intermediate **52** was prepared from **50** in 79% yield by a similar procedure as that for **51**. ^{1}H NMR (300 MHz, $CD_3OD:CCl_3D$=1:10) δ 7.84 (d, *J* = 8.7 Hz, 1H), 7.78 (d, *J* = 2.0 Hz, 1H), 7.72 (d, *J* = 7.5 Hz, 2H), 7.57-7.54 (m, 2H), 7.43 – 7.30 (m, 3H), 7.24 (t, *J* = 7.4 Hz, 2H), 4.95 – 4.66 (m, 1H), 4.45 – 4.25 (m, 2H), 4.19 (t, *J* = 7.0 Hz, 1H), 3.67-3.64 (m, 2H). ^{13}C NMR (75 MHz, $CD_3OD:CCl_3D$=1:10) δ 172.19, 167.48, 156.13, 151.13, 143.72, 143.66, 141.24, 136.32, 131.19, 127.68, 127.02, 126.96, 125.06, 123.37, 121.15, 119.93, 67.15, 52.92, 47.03, 35.67. UPLC-MS (ESI-MS) *m/z*: calculated for $C_{25}H_{20}ClN_2O_4S^+$ 479.08, found 479.19 $[M+H]^+$.

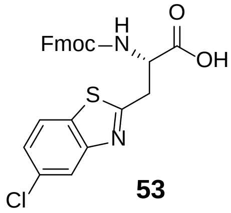

53

(S)-2-((((9H-fluoren-9-yl)methoxy)carbonyl)amino)-3-(5-chlorobenzo[d]thiazol-2-yl)propanoic acid (53): Intermediate **53** was prepared from **48** in 41% yield in two steps using a similar procedure as that for **51**. ^{1}H NMR (400 MHz, DMSO) δ 13.04 (br, 1H), 8.11 (d, *J* = 8.6 Hz, 1H), 8.02 (d, *J* = 1.9 Hz, 1H), 7.93 (d, *J* = 8.6 Hz, 1H), 7.88 (d, *J* = 7.5 Hz, 2H), 7.67-7.94 (m, 2H), 7.48 (dd, *J* = 8.6, 2.0 Hz, 1H), 7.41-7.38 (m, 2H), 7.30-7.22 (m, 2H), 4.54 (td, *J* = 9.5, 4.6 Hz, 1H), 4.29 – 4.27 (m, 2H), 4.20 (t, *J* = 6.8 Hz, 1H), 3.63 (dd, *J* = 15.2, 4.5 Hz, 1H), 3.47 (dd, *J* = 15.1, 9.9 Hz, 1H). ^{13}C NMR (101 MHz, DMSO) δ 172.59, 170.94, 156.41, 153.89,

144.21, 144.15, 141.17, 134.25, 131.33, 128.07, 127.48, 125.64, 125.57, 124.05, 122.28, 120.57, 66.15, 53.92, 47.05, 35.62. UPLC-MS (ESI-MS) *m/z*: calculated for $C_{25}H_{20}ClN_2O_4S^+$ 479.08, found 479.22[M+H] $^+$.

Solid-phase synthesis of peptides 1-29.

General method for synthesis of peptides 1-29: The peptides **1-29** were synthesized on an ABI 433 Peptide Synthesizer using Fmoc chemistry. Rink amide resin was used as the solid support and the coupling reagents were HBTU (*O*-(Benzotriazol-1-yl)-*N,N,N',N'*-tetramethyluronium hexafluorophosphate) and HOBt (1-Hydroxybenzotriazole hydrate). The crude peptides were cleaved from the resin by cleavage cocktail (TFA:TES:H_2O, 18 mL:0.5 mL:1 mL), which also led to removal of the protecting groups. The solution containing the cleaved peptides was evaporated and purified by RP-HPLC to give peptides **1-29**.

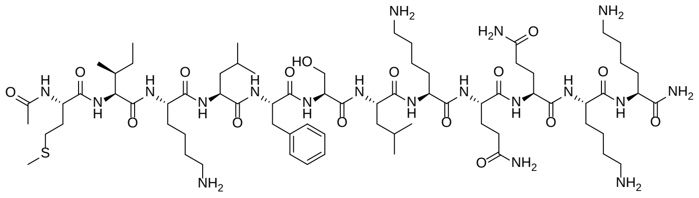

1

(S)-2-((5S,8S,11S,14S,17S,20S,23S,26S)-5-acetamido-11,26-bis(4-aminobutyl)-17-benzyl-8-((S)-sec-butyl)-20-(hydroxymethyl)-14,23-diisobutyl-6,9,12,15,18,21,24-heptaoxo-2-thia-7,10,13,16,19,22,25-heptaazaheptacosan-27-amido)-N1-((S)-5-amino-1-(((S)-6-amino-1-(((S)-1,6-diamino-1-oxohexan-2-yl)amino)-1-oxohexan-2-yl)amino)-1,5-dioxopentan-2-yl)pentanediamide (1): HRMS (ESI-MS) *m/z*: calculated for $C_{71}H_{127}N_{19}O_{16}S^{2+}$ 766.9709, found 766.9711 [M+2H] $^{2+}$.

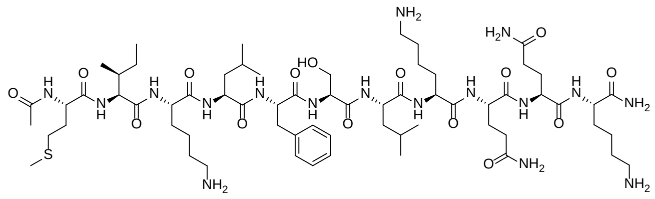

2

(S)-2-((5S,8S,11S,14S,17S,20S,23S,26S)-5-acetamido-11,26-bis(4-aminobutyl)-17-benzyl-8-((S)-sec-butyl)-20-(hydroxymethyl)-14,23-diisobutyl-6,9,12,15,18,21,24-heptaoxo-2-thia-7,10,13,16,19,22,25-heptaazaheptacosan-27-amido)-N1-((S)-5-amino-1-(((S)-1,6-diamino-1-oxohexan-2-yl)amino)-1,5-dioxopentan-2-yl)pentanediamide (2): HRMS (ESI-MS) *m/z*: calculated for $C_{65}H_{114}N_{17}O_{15}S^{+}$ 1404.8396, found 1404.8443 $[M+H]^{+}$.

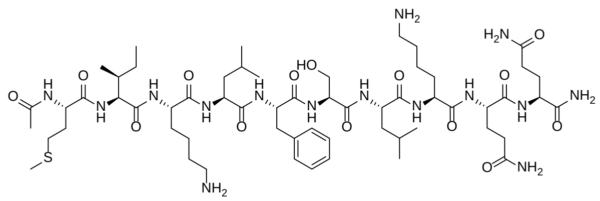

3

(S)-2-((5S,8S,11S,14S,17S,20S,23S,26S)-5-acetamido-11,26-bis(4-aminobutyl)-17-benzyl-8-((S)-sec-butyl)-20-(hydroxymethyl)-14,23-diisobutyl-6,9,12,15,18,21,24-heptaoxo-2-thia-7,10,13,16,19,22,25-heptaazaheptacosan-27-amido)-N1-((S)-1,5-diamino-1,5-dioxopentan-2-yl)pentanediamide (3): HRMS (ESI-MS) *m/z*: calculated for $C_{59}H_{102}N_{15}O_{14}S^{+}$ 1276.7446, found 1276.7440 $[M+H]^{+}$.

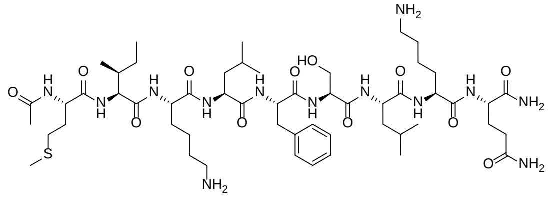

4

(S)-2-((5S,8S,11S,14S,17S,20S,23S,26S)-5-acetamido-11,26-bis(4-aminobutyl)-17-benzyl-8-((S)-sec-butyl)-20-(hydroxymethyl)-14,23-diisobutyl-6,9,12,15,18,21,24-heptaoxo-2-thia-7,10,13,16,19,22,25-

heptaazaheptacosan-27-amido)pentanediamide (4): HRMS (ESI-MS) *m/z*: calculated for $C_{54}H_{94}N_{13}O_{12}S^+$ 1148.6860, found 1148.6858 $[M+H]^+$.

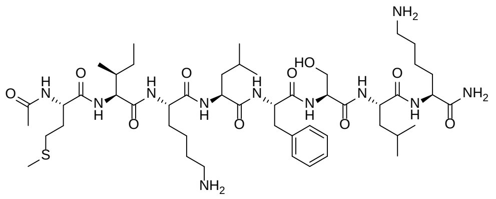

5

(S)-2-((2S,3S)-2-((S)-2-acetamido-4-(methylthio)butanamido)-3-methylpentanamido)-6-amino-N-((4S,7S,10S,13S,16S)-20-amino-7-benzyl-16-carbamoyl-10-(hydroxymethyl)-13-isobutyl-2-methyl-5,8,11,14-tetraoxo-6,9,12,15-tetraazaicosan-4-yl)hexanamide (5): HRMS (ESI-MS) *m/z*: calculated for $C_{49}H_{86}N_{11}O_{10}S^+$ 1020.6274, found 1020.6273 $[M+H]^+$.

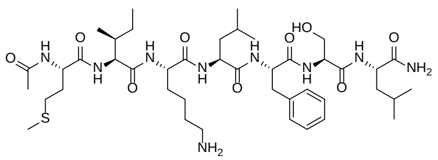

6

(S)-2-((2S,3S)-2-((S)-2-acetamido-4-(methylthio)butanamido)-3-methylpentanamido)-6-amino-N-((S)-1-(((S)-1-(((S)-1-(((S)-1-amino-4-methyl-1-oxopentan-2-yl)amino)-3-hydroxy-1-oxopropan-2-yl)amino)-1-oxo-3-phenylpropan-2-yl)amino)-4-methyl-1-oxopentan-2-yl)hexanamide (6): UPLC-MS (ESI-MS) *m/z*: calculated for $C_{43}H_{74}N_9O_9S^+$ 892.5325, found 892.5324 $[M+H]^+$.

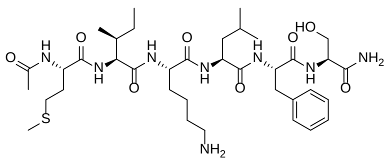

7

(S)-2-((2S,3S)-2-((S)-2-acetamido-4-(methylthio)butanamido)-3-methylpentanamido)-6-amino-N-((S)-1-(((S)-1-(((S)-1-amino-3-hydroxy-1-oxopropan-2-yl)amino)-1-oxo-3-phenylpropan-2-

yl)amino)-4-methyl-1-oxopentan-2-yl)hexanamide (7): HRMS (ESI-MS) *m/z*: calculated for $C_{37}H_{63}N_8O_8S^+$ 779.4484, found 779.4488 $[M+H]^+$.

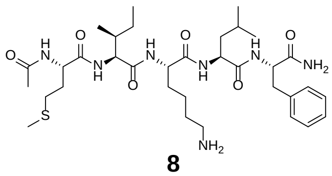

8

(S)-2-((2S,3S)-2-((S)-2-acetamido-4-(methylthio)butanamido)-3-methylpentanamido)-6-amino-N-((S)-1-(((S)-1-amino-1-oxo-3-phenylpropan-2-yl)amino)-4-methyl-1-oxopentan-2-yl)hexanamide (8): HRMS (ESI-MS) *m/z*: calculated for $C_{34}H_{58}N_7O_6S^+$ 692.4164, found 692.4164 $[M+H]^+$.

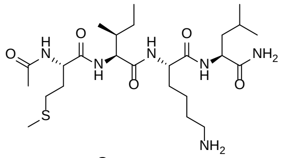

9

(S)-2-((2S,3S)-2-((S)-2-acetamido-4-(methylthio)butanamido)-3-methylpentanamido)-6-amino-N-((S)-1-amino-4-methyl-1-oxopentan-2-yl)hexanamide (9): HRMS (ESI-MS) *m/z*: calculated for $C_{25}H_{49}N_6O_5S^+$ 545.3480, found 545.3481 $[M+H]^+$.

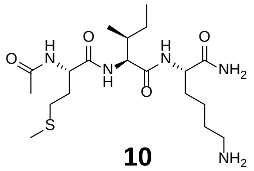

10

(S)-2-((2S,3S)-2-((S)-2-acetamido-4-(methylthio)butanamido)-3-methylpentanamido)-6-aminohexanamide (10): UPLC-MS (ESI-MS) *m/z*: calculated for $C_{19}H_{38}N_5O_4S^+$ 432.2639, found 432.2643 $[M+H]^+$.

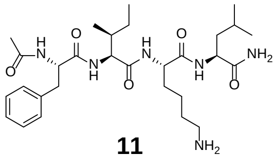

11

(S)-2-((2S,3S)-2-((S)-2-acetamido-3-phenylpropanamido)-3-methylpentanamido)-6-amino-N-((S)-1-amino-4-methyl-1-oxopentan-2-yl)hexanamide (11): HRMS (ESI-MS) *m/z*: calculated for $C_{29}H_{49}N_6O_5^+$ 561.3759, found 561.3760 $[M+H]^+$.

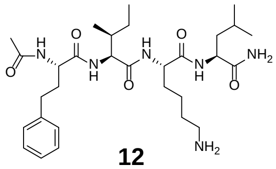

12

(S)-2-((2S,3S)-2-((S)-2-acetamido-4-phenylbutanamido)-3-methylpentanamido)-6-amino-N-((S)-1-amino-4-methyl-1-oxopentan-2-yl)hexanamide (12): HRMS (ESI-MS) *m/z*: calculated for $C_{30}H_{51}N_6O_5^+$ 575.3915, found 575.3918 $[M+H]^+$.

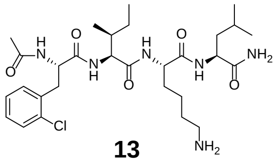

13

(S)-2-((2S,3S)-2-((S)-2-acetamido-3-(2-chlorophenyl)propanamido)-3-methylpentanamido)-6-amino-N-((S)-1-amino-4-methyl-1-oxopentan-2-yl)hexanamide (13): HRMS (ESI-MS) *m/z*: calculated for $C_{29}H_{48}ClN_6O_5^+$ 595.3369, found 595.3374 $[M+H]^+$.

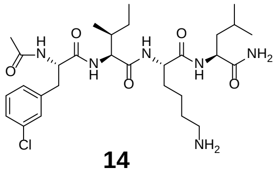

14

(S)-2-((2S,3S)-2-((S)-2-acetamido-3-(3-chlorophenyl)propanamido)-3-methylpentanamido)-6-amino-N-((S)-1-amino-4-methyl-1-oxopentan-2-yl)hexanamide (14): HRMS (ESI-MS) *m/z*: calculated for $C_{29}H_{48}ClN_6O_5^+$ 595.3369, found 595.3372 $[M+H]^+$.

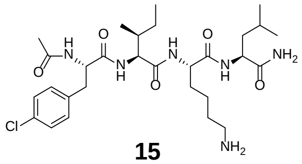

15

(S)-2-((2S,3S)-2-((S)-2-acetamido-3-(4-chlorophenyl)propanamido)-3-methylpentanamido)-6-amino-N-((S)-1-amino-4-methyl-1-oxopentan-2-yl)hexanamide (15): HRMS (ESI-MS) *m/z*: calculated for $C_{29}H_{48}ClN_6O_5^+$ 595.3369, found 595.3375 $[M+H]^+$.

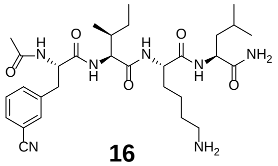

16

(S)-2-((2S,3S)-2-((S)-2-acetamido-3-(3-cyanophenyl)propanamido)-3-methylpentanamido)-6-amino-N-((S)-1-amino-4-methyl-1-oxopentan-2-yl)hexanamide (16): HRMS (ESI-MS) *m/z*: calculated for $C_{30}H_{48}N_7O_5^+$ 586.3711, found 586.3713 $[M+H]^+$.

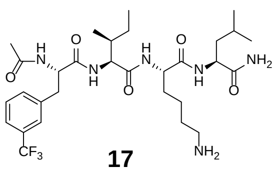

17

(S)-2-((2S,3S)-2-((S)-2-acetamido-3-(3-(trifluoromethyl)phenyl)propanamido)-3-methylpentanamido)-6-amino-N-((S)-1-amino-4-methyl-1-oxopentan-2-yl)hexanamide (17): HRMS (ESI-MS) *m/z*: calculated for $C_{30}H_{48}F_3N_6O_5^+$ 629.3633, found 629.3636 $[M+H]^+$.

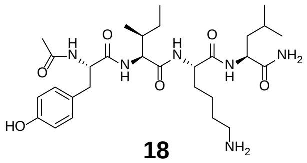

18

(S)-2-((2S,3S)-2-((S)-2-acetamido-3-(4-hydroxyphenyl)propanamido)-3-methylpentanamido)-6-amino-N-((S)-1-amino-4-methyl-1-oxopentan-2-yl)hexanamide (18): HRMS (ESI-MS) *m/z*: calculated for $C_{29}H_{49}N_6O_6^+$ 577.3708, found 577.3713 $[M+H]^+$.

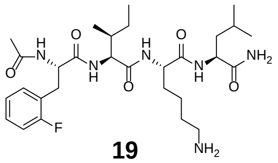

19

(S)-2-((2S,3S)-2-((S)-2-acetamido-3-(2-fluorophenyl)propanamido)-3-methylpentanamido)-6-amino-N-((S)-1-amino-4-methyl-1-oxopentan-2-yl)hexanamide (19): HRMS (ESI-MS) *m/z*: calculated for $C_{29}H_{48}FN_6O_5^+$ 579.3665, found 579.3671 $[M+H]^+$.

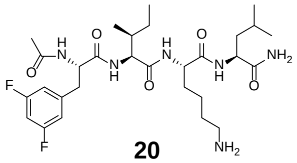

20

(S)-2-((2S,3S)-2-((S)-2-acetamido-3-(3,5-difluorophenyl)propanamido)-3-methylpentanamido)-6-amino-N-((S)-1-amino-4-methyl-1-oxopentan-2-yl)hexanamide (20): HRMS (ESI-MS) *m/z*: calculated for $C_{29}H_{47}F_2N_6O_5^+$ 597.3571, found 597.3570 $[M+H]^+$.

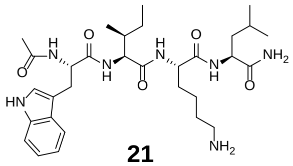

21

(S)-2-((2S,3S)-2-((S)-2-acetamido-3-(1H-indol-3-yl)propanamido)-3-methylpentanamido)-6-amino-N-((S)-1-amino-4-methyl-1-oxopentan-2-yl)hexanamide (21): HRMS (ESI-MS) *m/z*: calculated for $C_{31}H_{50}N_7O_5^+$ 600.3868, found 600.3867 $[M+H]^+$.

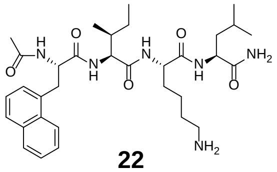

22

(S)-2-((2S,3S)-2-((S)-2-acetamido-3-(naphthalen-1-yl)propanamido)-3-methylpentanamido)-6-amino-N-((S)-1-amino-4-methyl-1-oxopentan-2-yl)hexanamide (22): HRMS (ESI-MS) *m/z*: calculated for $C_{33}H_{51}N_6O_5^+$ 611.3915, found 611.3916 $[M+H]^+$.

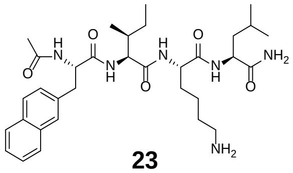

23

(S)-2-((2S,3S)-2-((S)-2-acetamido-3-(naphthalen-2-yl)propanamido)-3-methylpentanamido)-6-amino-N-((S)-1-amino-4-methyl-1-oxopentan-2-yl)hexanamide (23): HRMS (ESI-MS) *m/z*: calculated for $C_{33}H_{51}N_6O_5^+$ 611.3915, found 611.3914 $[M+H]^+$.

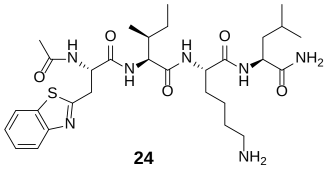

24

(S)-2-((2S,3S)-2-((S)-2-acetamido-3-(benzo[d]thiazol-2-yl)propanamido)-3-methylpentanamido)-6-amino-N-((S)-1-amino-4-methyl-1-oxopentan-2-yl)hexanamide (24): ^{1}H NMR (400 MHz, MeOD) δ 7.97 (d, *J* = 9.0 Hz, 2H), 7.60 – 7.48 (m, 1H), 7.48 – 7.38 (m, 1H), 4.98 (dd, *J* = 8.5, 5.2 Hz, 1H), 4.39 (ddd, *J* = 12.5, 9.2, 5.6 Hz, 2H), 4.22 (d, *J* = 7.6 Hz, 1H), 3.65 (dd, *J* = 15.4, 5.2 Hz, 1H), 3.50 (dd, *J* = 15.4, 8.5 Hz, 1H), 2.92 (t, *J* = 7.4 Hz, 2H), 2.01 (s,

3H), 1.91 – 1.77 (m, 2H), 1.76 – 1.38 (m, 9H), 1.21-1.15 (m, 1H), 1.01 – 0.83 (m, 12H). ^{13}C NMR (101 MHz, MeOD) δ 176.01, 172.18, 172.03, 171.35, 167.73, 152.61, 135.10, 126.01, 125.09, 122.04, 121.47, 58.17, 53.04, 52.78, 51.40, 40.76, 39.11, 36.63, 34.97, 30.76, 26.52, 24.62, 24.48, 22.15, 22.11, 21.14, 20.37, 14.51, 9.97. UPLC-MS (ESI-MS) *m/z*: calculated for $C_{30}H_{48}N_7O_5S^+$ 618.34, found 618.29 $[M+H]^+$.

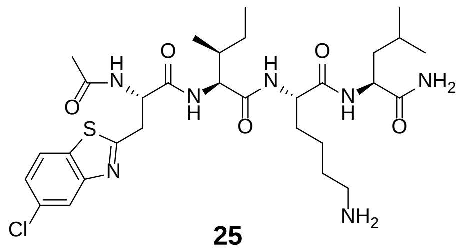

25

(S)-2-((2S,3S)-2-((S)-2-acetamido-3-(5-chlorobenzo[d]thiazol-2-yl)propanamido)-3-methylpentanamido)-6-amino-N-((S)-1-amino-4-methyl-1-oxopentan-2-yl)hexanamide (25): ^{1}H NMR (400 MHz, MeOD) δ 8.54 (d, *J* = 7.7 Hz, 1H), 8.23 (d, *J* = 7.7 Hz, 1H), 8.06 (d, *J* = 7.0 Hz, 2H), 7.98-7.95 (m, 2H), 7.45 (dd, *J* = 8.6, 2.1 Hz, 1H), 5.00 – 4.95 (m, 1H), 4.48 – 4.32 (m, 2H), 4.20 (t, *J* = 7.7 Hz, 1H), 3.65 (dd, *J* = 15.5, 5.3 Hz, 1H), 3.50 (dd, *J* = 15.5, 8.4 Hz, 1H), 2.93 (t, *J* = 7.4 Hz, 2H), 2.01 (s, 3H), 1.91 – 1.77 (m, 2H), 1.74 – 1.39 (m, 9H), 1.20-1.12 (m, 1H), 1.00 – 0.82 (m, 12H). ^{13}C NMR (101 MHz, MeOD) δ 176.00, 172.21, 172.12, 171.97, 171.21, 170.07, 153.57, 133.71, 131.95, 125.36, 122.63, 121.78, 58.10, 52.88, 52.71, 51.36, 40.80, 39.13, 36.65, 35.00, 30.79, 26.55, 24.58, 24.48, 22.14, 22.10, 21.13, 20.37, 14.49, 9.95. UPLC-MS (ESI-MS) *m/z*: calculated for $C_{30}H_{47}ClN_7O_5S^+$ 652.30, found 652.25 $[M+H]^+$.

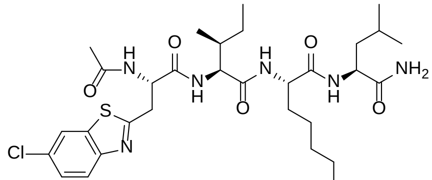

26

(S)-2-((2S,3S)-2-((S)-2-acetamido-3-(6-chlorobenzo[d]thiazol-2-yl)propanamido)-3-methylpentanamido)-6-amino-N-((S)-1-amino-4-methyl-1-oxopentan-2-yl)hexanamide (26): ^{1}H NMR (400 MHz, MeOD) δ 8.53 (d, *J* = 7.6 Hz, 1H), 8.23 (d, *J* = 7.5 Hz, 1H), 8.08-8.06 (m, 2H), 8.03 (d, *J* = 2.0 Hz, 1H), 7.92 (d, *J* = 8.7 Hz, 1H), 7.52 (dd, *J* = 8.7, 2.1 Hz, 1H), 5.00 – 4.93 (m, 1H), 4.50 – 4.30 (m, 2H), 4.20 (t, *J* = 7.7 Hz, 1H), 3.64 (dd, *J* = 15.5, 5.2 Hz, 1H), 3.48 (dd, *J* = 15.5, 8.4 Hz, 1H), 2.93 (t, *J* = 7.4 Hz, 2H), 2.01 (s, 3H), 1.90 – 1.78 (m, 2H), 1.75 – 1.34 (m, 9H), 1.23 – 1.09 (m, 1H), 1.06 – 0.73 (m, 12H). ^{13}C NMR (101 MHz, MeOD) δ 176.00, 172.18, 172.13, 171.96, 171.22, 168.56, 151.37, 136.58, 130.86, 126.64, 123.07, 121.14, 58.10, 52.90, 52.73, 51.36, 40.79, 39.13, 36.63, 34.92, 30.77, 26.55, 24.58, 24.47, 22.14, 22.10, 21.12, 20.36, 14.49, 9.95. UPLC-MS (ESI-MS) *m/z*: calculated for $C_{30}H_{47}ClN_7O_5S^+$ 652.30, found 652.28 $[M+H]^+$.

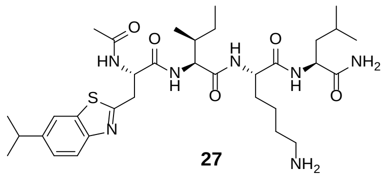

27

(S)-2-((2S,3S)-2-((S)-2-acetamido-3-(6-isopropylbenzo[d]thiazol-2-yl)propanamido)-3-methylpentanamido)-6-amino-N-((S)-1-amino-4-methyl-1-oxopentan-2-yl)hexanamide (27): ^{1}H NMR (400 MHz, MeOD) δ 7.87 (d, *J* = 8.5 Hz, 1H), 7.82 (d, *J* = 1.7 Hz, 1H), 7.42 (dd, *J* = 8.5, 1.7 Hz, 1H), 4.94 (dd, *J* = 8.6, 5.0 Hz, 1H), 4.43-4.36 (m, 2H), 4.20 (d, *J* = 7.6 Hz, 1H), 3.62 (dd, *J* = 15.4, 5.0 Hz, 1H), 3.47 (dd, *J* = 15.4, 8.7 Hz, 1H), 3.08 (dt, *J* = 13.8, 6.9 Hz, 1H), 2.93 (t,

J = 7.4 Hz, 2H), 2.01 (s, 3H), 1.90 – 1.79 (m, 2H), 1.77 – 1.39 (m, 9H), 1.33 (d, *J* = 6.9 Hz, 6H), 1.19-1.12 (m, 1H), 0.98-0.85 (m, 12H). ^{13}C NMR (101 MHz, MeOD) δ 176.00, 172.19, 172.15, 172.05, 171.39, 166.88, 150.96, 146.66, 135.29, 125.15, 121.69, 118.56, 58.20, 53.11, 52.73, 51.36, 40.79, 39.13, 36.56, 34.88, 34.09, 30.74, 26.51, 24.60, 24.48, 23.16, 22.15, 22.09, 21.13, 20.36, 14.50, 9.95. UPLC-MS (ESI-MS) *m/z*: calculated for $C_{33}H_{54}N_7O_5S^+$ 660.39, found 661.01 $[M+H]^+$.

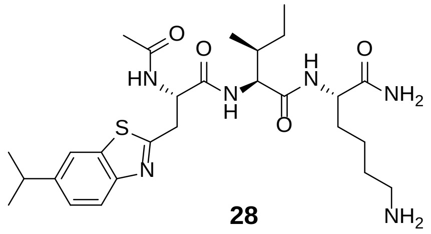

28

(S)-2-((2S,3S)-2-((S)-2-acetamido-3-(6-isopropylbenzo[d]thiazol-2-yl)propanamido)-3-methylpentanamido)-6-aminohexanamide (28): ^{1}H NMR (400 MHz, MeOD) δ 8.53 (d, *J* = 7.7 Hz, 1H), 8.21 (d, *J* = 8.0 Hz, 1H), 8.08 (d, *J* = 7.7 Hz, 1H), 7.87 (d, *J* = 8.5 Hz, 1H), 7.82 (d, *J* = 1.7 Hz, 1H), 7.42 (dd, *J* = 8.5, 1.7 Hz, 1H), 4.94 (dd, *J* = 8.6, 5.1 Hz, 1H), 4.44 – 4.30 (m, 1H), 4.19 (t, *J* = 7.7 Hz, 1H), 3.63 (dd, *J* = 15.4, 5.1 Hz, 1H), 3.46 (dd, *J* = 15.4, 8.6 Hz, 1H), 3.08 (dt, *J* = 13.8, 6.9 Hz, 1H), 2.93 (t, *J* = 7.5 Hz, 2H), 2.01 (s, 3H), 1.94 – 1.80 (m, 2H), 1.76 – 1.60 (m, 3H), 1.56 – 1.38 (m, 3H), 1.33 (d, *J* = 6.9 Hz, 6H), 1.20-1.13 (m, 1H), 0.95 (d, *J* = 6.8 Hz, 3H), 0.88 (t, *J* = 7.4 Hz, 3H). ^{13}C NMR (101 MHz, MeOD) δ 174.86, 172.20, 172.10, 171.48, 166.94, 150.94, 146.68, 135.28, 125.16, 121.67, 118.57, 58.35, 53.07, 52.35, 39.16, 36.38, 34.85, 34.09, 30.97, 26.53, 24.59, 23.16, 22.25, 21.14, 14.51, 9.89. UPLC-MS (ESI-MS) *m/z*: calculated for $C_{27}H_{43}N_6O_4S^+$ 547.31, found 547.22 $[M+H]^+$.

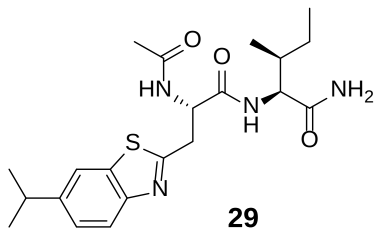

29

(2S,3S)-2-((S)-2-acetamido-3-(6-isopropylbenzo[d]thiazol-2-yl)propanamido)-3-methylpentanamide (29): ^{1}H NMR (400 MHz, MeOD) δ 7.86 (d, *J* = 8.5 Hz, 1H), 7.81 (d, *J* = 1.7 Hz, 1H), 7.41 (dd, *J* = 8.5, 1.7 Hz, 1H), 5.01 – 4.95 (m, 1H), 4.32 – 4.22 (m, 1H), 3.64 (dd, *J* = 15.3, 5.7 Hz, 1H), 3.46 (dd, *J* = 15.3, 8.0 Hz, 1H), 3.07 (dt, *J* = 13.8, 6.9 Hz, 1H), 1.99 (s, 3H), 1.93 – 1.80 (m, 1H), 1.56-1.50 (m, 1H), 1.33 (d, *J* = 6.9 Hz, 6H), 1.22-1.14 (m, 1H), 0.95 (d, *J* = 6.8 Hz, 3H), 0.91 (t, *J* = 7.4 Hz, 3H). ^{13}C NMR (101 MHz, MeOD) δ 174.65, 172.01, 171.07, 167.02, 150.89, 146.59, 135.26, 125.14, 121.58, 118.52, 57.81, 52.73, 36.70, 34.95, 34.08, 24.26, 23.15, 21.06, 14.58, 10.27. UPLC-MS (ESI-MS) *m/z*: calculated for $C_{21}H_{31}N_4O_3S^+$ 419.21, found 419.27 $[M+H]^+$.

Synthesis of compounds 30-36.

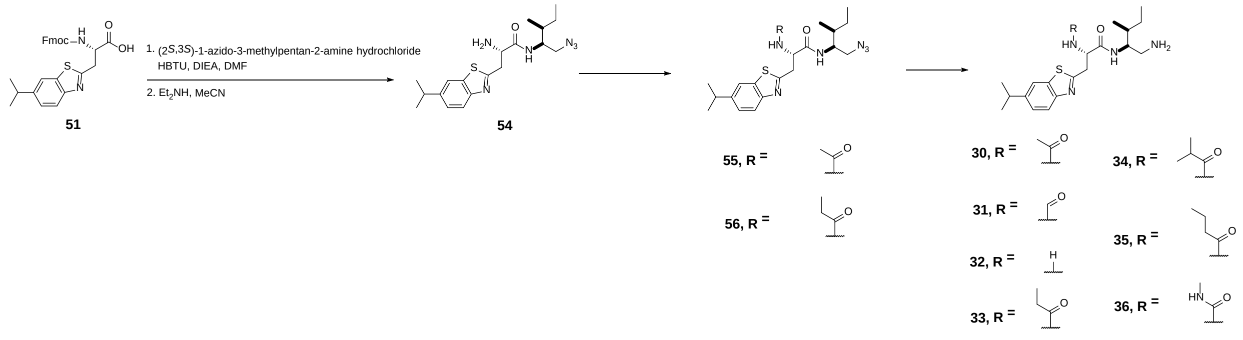

(2S,3S)-1-azido-3-methylpentan-2-amine hydrochloride was synthesized as reported[2].

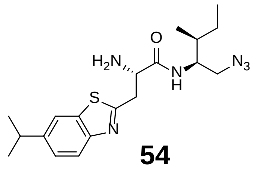

54

(S)-2-amino-N-((2S,3S)-1-azido-3-methylpentan-2-yl)-3-(6-isopropylbenzo[d]thiazol-2-yl)propanamide (54): To a solution of **51** (2.0 g, 4.1 mmol, 1 equiv.), HBTU (2.3 g, 6.2 mmol, 1.5 equiv.) and DIEA (2.1 mL, 12.3 mmol, 3 equiv.) in DMF (20 mL) was added (2S,3S)-1-azido-3-methylpentan-2-amine hydrochloride (0.8 g, 4.5 mmol, 1.1 equiv.). The resultant mixture was stirred at room temperature for 1 h. The solution was diluted with EtOAc and washed with H_2O, saturated sodium bicarbonate, 1.0 M HCl, brine and dried over sodium sulfate. After removal of the solvent under vacuum, the residue was treated with 3 mL diethylamine in Actonitrile (27 mL) for 1 h. The reaction mixture was evaporated and the residue was purified by flash chromatography on silica gel to afford **54** (1.2 g 77%). ^{1}H NMR (400 MHz, MeOD) δ 7.94 (d, *J* = 8.5 Hz, 1H), 7.84 (d, *J* = 1.7 Hz, 1H), 7.44 (dd, *J* = 8.5, 1.7 Hz, 1H), 4.57 (dd, *J* = 7.8, 5.2 Hz, 1H), 3.87 (td, *J* = 7.3, 3.8 Hz, 1H), 3.77 (dd, *J* = 16.6, 5.2 Hz, 1H), 3.68 (dd, *J* = 16.6, 7.8 Hz, 1H), 3.47 (dd, *J* = 12.8, 3.9 Hz, 1H), 3.41 – 3.35 (m, 1H), 3.06 (dq, *J* = 13.6, 6.8 Hz, 1H), 1.70 – 1.60 (m, 1H), 1.58-1.50 (m, 1H), 1.32 (d, *J* = 6.9 Hz, 6H), 1.24 – 1.12 (m, 1H), 0.96-0.91 (m, 6H). ^{13}C NMR (101 MHz, MeOD) δ 167.35, 164.24, 151.02, 146.98, 135.29, 125.36, 122.10, 118.60, 53.74, 52.01, 36.06, 34.11, 34.05, 24.98, 23.15, 14.16, 10.11. UPLC-MS (ESI-MS) *m/z*: calculated for $C_{19}H_{29}N_6OS^+$ 389.21, found 389.36[M+H] $^+$.

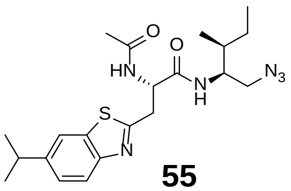

55

(S)-2-acetamido-N-((2S,3S)-1-azido-3-methylpentan-2-yl)-3-(6-isopropylbenzo[d]thiazol-2-yl)propanamide (55): Acetic anhydride (46 mg, 0.45 mmoL, 2 equiv.) was added to a solution of **54** (87 mg, 0.22 mmoL, 1 equiv.) and DIEA (156 µL, 0.89 mmol, 4 equiv.) in DCM (10 mL).

The resulting reaction mixture was stirred for half an hour and then was evaporated. The residue was purified by flash chromatography on silica gel to afford compound **55** (89 mg, 92% yields). ^{1}H NMR (400 MHz, $CDCl_3$) δ 7.91 (d, *J* = 8.5 Hz, 1H), 7.72 (d, *J* = 1.6 Hz, 1H), 7.59 (d, *J* = 9.1 Hz, 1H), 7.47 – 7.36 (m, 2H), 5.06 (q, *J* = 6.4 Hz, 1H), 4.02 – 3.82 (m, 1H), 3.64 (d, *J* = 6.3 Hz, 2H), 3.37 (qd, *J* = 12.6, 5.3 Hz, 2H), 3.08 (dt, *J* = 13.8, 6.9 Hz, 1H), 2.08 (s, 3H), 1.65-1.58 (m, 1H), 1.51 – 1.39 (m, 1H), 1.33 (d, *J* = 6.9 Hz, 6H), 1.21 – 1.03 (m, 1H), 0.92 (d, *J* = 6.8 Hz, 3H), 0.88 (t, *J* = 7.4 Hz, 3H). ^{13}C NMR (101 MHz, $CDCl_3$) δ 171.12, 169.86, 168.43, 148.60, 147.43, 134.34, 126.28, 121.25, 118.98, 53.54, 52.41, 52.37, 36.15, 35.62, 34.29, 25.06, 24.10, 22.97, 15.39, 11.19. UPLC-MS (ESI-MS) *m/z*: calculated for $C_{21}H_{31}N_6O_2S^+$ 431.22, found 431.36[M+H]$^+$.

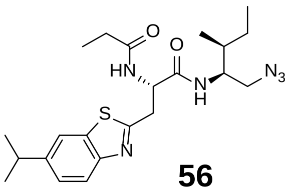

56

(S)-N-((2S,3S)-1-azido-3-methylpentan-2-yl)-3-(6-isopropylbenzo[d]thiazol-2-yl)-2-propionamidopropanamide (56): Propionic anhydride (58 mg, 0.45 mmoL, 2 equiv.) was added to a solution of **54** (87 mg, 0.22 mmoL, 1 equiv.) and DIEA (156 µL, 0.89 mmol, 4 equiv.) in DCM (10 mL). The resulting reaction mixture was stirred for half an hour and then was evaporated. The residue was purified by flash chromatography on silica gel to afford compound **56** (86 mg, 89% yields). ^{1}H NMR (400 MHz, $CDCl_3$) δ 7.94 (d, *J* = 8.5 Hz, 1H), 7.73 (d, *J* = 1.0 Hz, 1H), 7.54 (d, *J* = 9.0 Hz, 1H), 7.50 (d, *J* = 7.0 Hz, 1H), 7.45 (dd, *J* = 8.4, 1.5 Hz, 1H), 5.08 (dd, *J* = 12.7, 6.8 Hz, 1H), 4.00 – 3.88 (m, 1H), 3.75 (dd, *J* = 15.3, 5.3 Hz, 1H), 3.63 (dd, *J* = 15.3, 7.2 Hz, 1H), 3.39 (qd, *J* = 12.6, 5.4 Hz, 2H), 3.08 (dt, *J* = 13.8, 6.9 Hz, 1H), 2.31 (q, *J* = 7.6 Hz, 2H), 1.68 – 1.56 (m, 1H), 1.48-1.42 (m, 1H), 1.33 (d, *J* = 6.9 Hz, 6H), 1.20 – 1.05 (m, 4H),

0.92 (d, J = 6.8 Hz, 3H), 0.88 (t, J = 7.4 Hz, 3H). ^{13}C NMR (101 MHz, $CDCl_3$) δ 175.27, 169.85, 169.08, 147.86, 147.47, 133.89, 126.67, 120.89, 119.04, 53.65, 52.49, 52.36, 36.20, 35.35, 34.30, 29.42, 25.07, 24.05, 24.04, 15.32, 11.17, 9.61. UPLC-MS (ESI-MS) *m/z*: calculated for $C_{22}H_{33}N_6O_2S^+$ 445.24, found 445.37[M+H]$^+$.

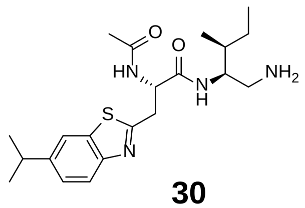

30

(S)-2-acetamido-N-((2S,3S)-1-amino-3-methylpentan-2-yl)-3-(6-isopropylbenzo[d]thiazol-2-yl)propanamide, (30): To a solution of compound **55** (45 mg, 0.11 mmol) in MeOH (10 mL), 10% Pd-C (20 mg) was added. The solution was stirred under 1 atm of H_2 at room temperature for 3 hours before filtering through celite and being concentrated. The resulting amine was purified by HPLC to afford compound **30** (38 mg, 91%). ^{1}H NMR (400 MHz, MeOD) δ 7.92 – 7.77 (m, 2H), 7.49 – 7.36 (m, 1H), 4.87-4.85 (m, 1H), 4.01-3.96 (m, 1H), 3.69 (dd, J = 15.2, 5.9 Hz, 1H), 3.55 (dd, J = 15.2, 6.9 Hz, 1H), 3.30 – 3.19 (m, 1H), 3.08 (dt, J = 13.8, 6.9 Hz, 1H), 2.97 (dd, J = 12.6, 11.3 Hz, 1H), 2.03 (s, 3H), 1.68 – 1.54 (m, 1H), 1.48-1.41 (m, 1H), 1.33 (d, J = 6.9 Hz, 6H), 1.21 – 1.06 (m, 1H), 0.94 (d, J = 6.8 Hz, 3H), 0.86 (t, J = 7.4 Hz, 3H). ^{13}C NMR (101 MHz, MeOD) δ 172.45, 171.81, 167.06, 150.90, 146.80, 135.18, 125.31, 121.29, 118.67, 53.09, 52.09, 41.76, 36.51, 34.60, 34.08, 24.77, 23.14, 21.18, 14.22, 9.75. UPLC-MS (ESI-MS) *m/z*: calculated for $C_{21}H_{33}N_4O_2S^+$ 405.23, found 405.25[M+H]$^+$.

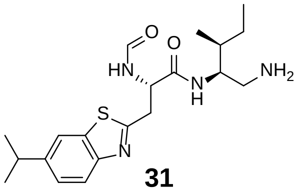

31

(S)-N-((2S,3S)-1-amino-3-methylpentan-2-yl)-2-formamido-3-(6-isopropylbenzo[d]thiazol-2-yl)propanamide (31): Compound **54** (100 mg, 0.26 mmol) was dissolved in a mixture of DIEA (1 mL) and Ethyl formate (5 mL) and the resulting reaction mixture was left stirring for 3 days at room temperature. The solvents were removed *in vacuo* and the residue was dissolved in MeOH (10ml). Then 10% Pd-C (20 mg) was added and the resulting reaction mixture was stirred under 1 atm of H_2 at room temperature for 3 hours before filtering through celite and being concentrated. The resulting amine was purified by HPLC to afford compound **31** (58 mg, 58%). ^{1}H NMR (400 MHz, MeOD) δ 8.18 (d, *J* = 0.7 Hz, 1H), 7.87 – 7.81 (m, 2H), 7.43 (dd, *J* = 8.5, 1.7 Hz, 1H), 4.99 (t, *J* = 5.6 Hz, 1H), 4.04-3.98 (m, 1H), 3.69 (dd, *J* = 15.3, 5.8 Hz, 1H), 3.63 (dd, *J* = 15.3, 6.1 Hz, 1H), 3.27 (dd, *J* = 12.9, 2.4 Hz, 1H), 3.08 (dt, *J* = 13.8, 6.9 Hz, 1H), 2.98 (dd, *J* = 12.9, 11.1 Hz, 1H), 1.68 – 1.55 (m, 1H), 1.48-1.42 (m, 1H), 1.33 (d, *J* = 6.9 Hz, 6H), 1.21 – 1.08 (m, 1H), 0.94 (d, *J* = 6.8 Hz, 3H), 0.85 (t, *J* = 7.4 Hz, 3H). ^{13}C NMR (101 MHz, MeOD) δ 171.17, 166.62, 162.68, 150.95, 146.81, 135.20, 125.30, 121.29, 118.67, 52.15, 52.06, 51.53, 41.84, 36.56, 34.73, 34.07, 24.76, 23.13, 14.20, 9.73. UPLC-MS (ESI-MS) *m/z*: calculated for $C_{20}H_{31}N_4O_2S^+$ 391.22, found 391.22 $[M+H]^+$.

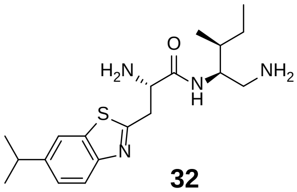

32

(S)-2-amino-N-((2S,3S)-1-amino-3-methylpentan-2-yl)-3-(6-isopropylbenzo[d]thiazol-2-yl)propanamide (32): To a solution of compound **54** (36 mg, 0.09 mmol) in MeOH (10 mL), was added 10% Pd-C (20 mg). The solution was stirred under 1 atm of H_2 at room temperature for 3 hours before filtering through celite and being concentrated. The resulting amine was purified by HPLC to afford **32** (28 mg, 83%). ^{1}H NMR (400 MHz, MeOD) δ 7.97 – 7.83 (m, 2H), 7.48 (dd, *J* = 8.6, 1.6 Hz, 1H), 4.54 (t, *J* = 5.8 Hz, 1H), 4.14-4.09 (m, 1H), 3.82-3.80(m, 2H), 3.29-3.28 (m, 1H), 3.13-3.03 (m, 2H), 1.73-1.67 (m, 1H), 1.60 – 1.47 (m, 1H), 1.34 (d, *J* = 6.9 Hz, 6H), 1.28-1.20 (m, 1H), 1.01 (d, *J* = 6.8 Hz, 3H), 0.95 (t, *J* = 7.4 Hz, 3H). ^{13}C NMR (101 MHz, MeOD) δ 168.43, 164.33, 151.03, 147.22, 135.21, 125.60, 121.54, 118.81, 52.49, 52.02, 41.45, 36.70, 34.10, 33.67, 24.83, 23.10, 14.05, 10.01. UPLC-MS (ESI-MS) *m/z*: calculated for $C_{19}H_{32}N_4OS^{2+}$ 182.11, found 182.21 $[M+2H]^{2+}$.

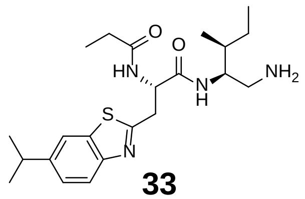

33

(S)-N-((2S,3S)-1-amino-3-methylpentan-2-yl)-3-(6-isopropylbenzo[d]thiazol-2-yl)-2-propionamidopropanamide (33): To a solution of compound **56** (52 mg, 0.12 mmol) in MeOH (10 mL) was added 10% Pd-C (20 mg). The solution was stirred under 1 atm of H_2 at room temperature for 3 hours before filtering through celite and being concentrated. The resulting amine was purified by HPLC to afford compound **33** (36 mg, 86%). ^{1}H NMR (400 MHz, MeOD) δ 7.90 – 7.80 (m, 2H), 7.43 (dd, *J* = 8.5, 1.7 Hz, 1H), 4.91-4.89 (m, 1H), 4.09 – 3.92 (m, 1H), 3.69 (dd, *J* = 15.2, 5.9 Hz, 1H), 3.55 (dd, *J* = 15.2, 7.0 Hz, 1H), 3.29 – 3.20 (m, 1H), 3.08 (dt, *J* = 13.8, 6.9 Hz, 1H), 3.02 – 2.93 (m, 1H), 2.30 (q, *J* = 7.6 Hz, 2H), 1.67 – 1.56 (m, 1H), 1.48-1.42

(m, 1H), 1.33 (d, *J* = 6.9 Hz, 6H), 1.20 – 1.07 (m, 4H), 0.95 (d, *J* = 6.8 Hz, 3H), 0.86 (t, *J* = 7.4 Hz, 3H). ^{13}C NMR (101 MHz, MeOD) δ 176.05, 171.86, 167.09, 150.90, 146.78, 135.16, 125.30, 121.28, 118.66, 52.92, 52.07, 41.77, 36.54, 34.55, 34.07, 28.54, 24.74, 23.14, 14.24, 9.76, 8.71. UPLC-MS (ESI-MS) *m/z*: calculated for $C_{22}H_{35}N_4O_2S^+$ 419.25, found 419.29 $[M+H]^+$.

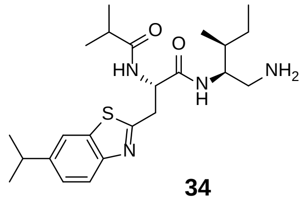

34

(S)-N-((2S,3S)-1-amino-3-methylpentan-2-yl)-2-isobutyramido-3-(6-isopropylbenzo[d]thiazol-2-yl)propanamide (34): Compound **34** was prepared from **54** in 72% yield over two steps by a similar procedure as that for compound **30**. ^{1}H NMR (400 MHz, MeOD) δ 8.33 (d, *J* = 7.3 Hz, 1H), 7.99 (d, *J* = 8.9 Hz, 1H), 7.85-7.83 (m, 2H), 7.43 (dd, *J* = 8.6, 1.6 Hz, 1H), 4.91 – 4.88 (m, 1H), 4.09 – 3.90 (m, 1H), 3.69 (dd, *J* = 15.2, 5.9 Hz, 1H), 3.55 (dd, *J* = 15.2, 7.1 Hz, 1H), 3.26 (dd, *J* = 13.4, 3.1 Hz, 1H), 3.08 (dt, *J* = 13.8, 6.9 Hz, 1H), 3.02 – 2.93 (m, 1H), 2.53 (dt, *J* = 13.7, 6.9 Hz, 1H), 1.68 – 1.57 (m, 1H), 1.50-1.42 (m, 1H), 1.33 (d, *J* = 6.9 Hz, 6H), 1.23 – 1.04 (m, 7H), 0.95 (d, *J* = 6.8 Hz, 3H), 0.88 (t, *J* = 7.4 Hz, 3H). ^{13}C NMR (101 MHz, MeOD) δ 179.04, 171.85, 167.11, 150.91, 146.78, 135.14, 125.30, 121.24, 118.67, 52.71, 52.02, 41.85, 36.59, 34.59, 34.45, 34.07, 24.69, 23.13, 18.48, 18.17, 14.25, 9.78. UPLC-MS (ESI-MS) *m/z*: calculated for $C_{23}H_{3}7N_4O_2S^+$ 433.26, found 433.29 $[M+H]^+$.

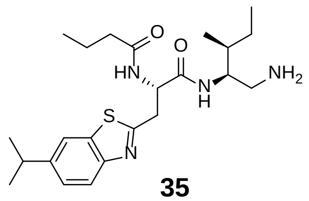

35

N-((S)-1-(((2S,3S)-1-amino-3-methylpentan-2-yl)amino)-3-(6-isopropylbenzo[d]thiazol-2-yl)-1-oxopropan-2-yl)butyramide (35): Compound **35** was prepared from **54** in 70% yield over two steps by a similar procedure as that for compound **30**. ^{1}H NMR (400 MHz, MeOD) δ 7.91 – 7.75 (m, 2H), 7.43 (dd, *J* = 8.6, 1.6 Hz, 1H), 4.91 – 4.88 (m, 1H), 4.02-3.96 (m, 1H), 3.69 (dd, *J* = 15.2, 5.9 Hz, 1H), 3.54 (dd, *J* = 15.2, 7.2 Hz, 1H), 3.25 (dd, *J* = 13.1, 3.1 Hz, 1H), 3.08 (dt, *J* = 13.8, 6.9 Hz, 1H), 2.98 (dd, *J* = 12.8, 11.1 Hz, 1H), 2.35 – 2.18 (m, 2H), 1.71 – 1.55 (m, 3H), 1.49-1.41 (m, 1H), 1.33 (d, *J* = 6.9 Hz, 6H), 1.19-1.12 (m, 1H), 0.96-0.85 (m, 9H). ^{13}C NMR (101 MHz, MeOD) δ 175.14, 171.86, 167.08, 150.93, 146.77, 135.17, 125.29, 121.28, 118.66, 52.90, 52.06, 41.78, 37.32, 36.56, 34.61, 34.07, 24.74, 23.14, 18.76, 14.23, 12.56, 9.78. UPLC-MS (ESI-MS) *m/z*: calculated for $C_{23}H_{3}7N_{4}O_{2}S^{+}$ 433.26, found 433.29 [M+H]$^{+}$.

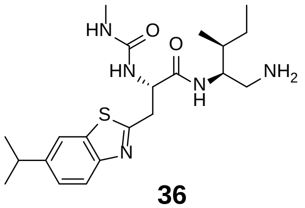

36

(S)-N-((2S,3S)-1-amino-3-methylpentan-2-yl)-3-(6-isopropylbenzo[d]thiazol-2-yl)-2-(3-methylureido)propanamide (36): Methyl isocyanate (18 mg, 0.31 mmol, 2 equiv.) was added to a solution of **54** (60 mg, 0.15 mmol, 1 equiv.) and DIEA (54 µL, 0.31 mmol, 2 equiv.) in CH_2Cl_2 (5 mL) and the resulting solution was stirred at room temperature overnight. The reaction mixture was concentrated and the residue was dissolved in MeOH (10ml). Then 10% Pd-C (20 mg) was added and the resulting reaction mixture was stirred under 1 atm of H_2 at room temperature for 3 hours before filtering through celite and being concentrated. The resulting amine was purified by HPLC to afford compound **36** (58 mg, 74%). ^{1}H NMR (400 MHz, MeOD) δ 7.85 (d, *J* = 8.5 Hz, 1H), 7.83 (d, *J* = 1.7 Hz, 1H), 7.42 (dd, *J* = 8.5, 1.7 Hz, 1H),

4.74 (t, J = 5.8 Hz, 1H), 3.99-3.94 (m, 1H), 3.61 (d, J = 5.8 Hz, 2H), 3.24 (dd, J = 12.9, 3.0 Hz, 1H), 3.07 (dt, J = 13.8, 6.9 Hz, 1H), 2.98 (dd, J = 12.8, 11.3 Hz, 1H), 2.74 (s, 3H), 1.58 (dtd, J = 8.8, 7.3, 3.7 Hz, 1H), 1.44 – 1.35 (m, 1H), 1.32 (d, J = 6.9 Hz, 6H), 1.12-1.04 (m, 1H), 0.91 (d, J = 6.8 Hz, 3H), 0.79 (t, J = 7.4 Hz, 3H). ^{13}C NMR (101 MHz, MeOD) δ 172.94, 167.09, 159.77, 150.97, 146.68, 135.23, 125.18, 121.40, 118.59, 53.78, 52.00, 41.73, 36.52, 35.24, 34.07, 25.54, 24.79, 23.15, 14.24, 9.70. UPLC-MS (ESI-MS) *m/z*: calculated for $C_{21}H_{34}N_5O_2S^+$ 420.24, found 419.29 [M+H]$^+$.

Synthesis of compounds 37-39.

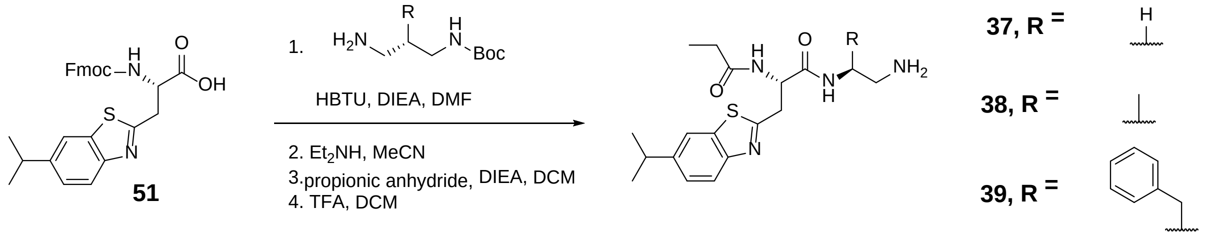

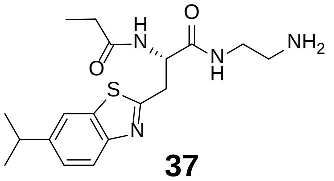

37

(S)-N-(2-aminoethyl)-3-(6-isopropylbenzo[d]thiazol-2-yl)-2-propionamidopropanamide (37): To a solution of **51** (120 mg, 0.25 mmol, 1 equiv.), HBTU (140 mg, 0.37 mmol, 1.5 equiv.) and DIEA (129 µL, 0.74 mmol, 3 equiv.) in DMF (5 mL) was added tert-butyl (2-aminoethyl)carbamate (43 mg, 0.27 mmol, 1.1 equiv.). The resultant mixture was stirred at room temperature for 1 h. The solution was diluted with EtOAc and washed with H_2O, saturated

sodium bicarbonate, 1.0 M HCl, brine and dried over sodium sulfate. After removal of the solvent under vacuum, the residue was treated with 1 mL diethylamine in Actonitrile (9 mL) for 1 h. The reaction mixture was evaporated and dissolved in DCM (5 mL). This solution was treated with propionic anhydride (64 mg, 0.49 mmoL, 2 equiv.) and DIEA (171 µL, 0.99 mmol, 4 equiv.). The resulting reaction mixture was stirred for half an hour and then was evaporated. The residue was treated with TFA (1 ml) in DCM (5 mL) and stirred for 5 h. This reaction mixture was concentrated and purified by HPLC to afford **37** (54 mg, 61%). ^{1}H NMR (400 MHz, MeOD) δ 7.86 (d, *J* = 8.5 Hz, 1H), 7.82 (d, *J* = 1.7 Hz, 1H), 7.42 (dd, *J* = 8.5, 1.7 Hz, 1H), 4.83 (dd, *J* = 8.2, 5.5 Hz, 1H), 3.67 (dd, *J* = 15.1, 5.5 Hz, 1H), 3.56 – 3.43 (m, 3H), 3.10-3.03 (m, 3H), 2.28 (q, *J* = 7.6 Hz, 2H), 1.32 (d, *J* = 6.9 Hz, 6H), 1.09 (t, *J* = 7.6 Hz, 3H). ^{13}C NMR (101 MHz, MeOD) δ 176.04, 172.38, 166.94, 150.80, 146.73, 135.22, 125.26, 121.46, 118.60, 53.15, 39.46, 36.83, 34.93, 34.07, 28.50, 23.14, 8.56. UPLC-MS (ESI-MS) *m/z*: calculated for $C_{18}H_{27}N_4O_2S^+$ 363.18, found 363.18 $[M+H]^+$.

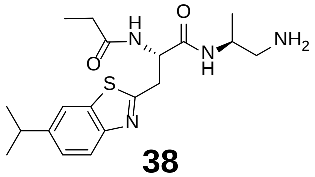

38

(S)-N-((S)-1-aminopropan-2-yl)-3-(6-isopropylbenzo[d]thiazol-2-yl)-2-propionamidopropanamide (38): Compound **38** was prepared from **51** in 57% yield over four steps by a similar procedure as that for compound **37**. ^{1}H NMR (400 MHz, MeOD) δ 7.86-7.83 (m, 2H), 7.42 (dd, *J* = 8.5, 1.7 Hz, 1H), 4.84 (dd, *J* = 7.1, 5.8 Hz, 1H), 4.31 – 4.15 (m, 1H), 3.69 (dd, *J* = 15.0, 5.8 Hz, 1H), 3.51 (dd, *J* = 15.0, 7.2 Hz, 1H), 3.17 – 2.91 (m, 3H), 2.28 (q, *J* = 7.6 Hz, 2H), 1.32 (d, *J* = 6.9 Hz, 6H), 1.25 (d, *J* = 6.9 Hz, 3H), 1.10 (t, *J* = 7.6 Hz, 3H). ^{13}C NMR (101 MHz, MeOD) δ 175.90, 171.51, 166.97, 150.86, 146.75, 135.21, 125.29, 121.29, 118.65,

52.91, 44.65, 43.71, 35.02, 34.07, 28.52, 23.14, 16.39, 8.65. UPLC-MS (ESI-MS) *m/z*: calculated for $C_{19}H_{29}N_4O_2S^+$ 377.20, found 377.23 $[M+H]^+$.

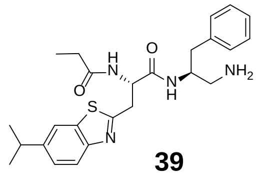

39

(S)-N-((S)-1-amino-3-phenylpropan-2-yl)-3-(6-isopropylbenzo[d]thiazol-2-yl)-2-propionamidopropanamide (39): Compound **39** was prepared from **51** in 53% yield over four steps by a similar procedure as that for compound **37**. ^{1}H NMR (400 MHz, MeOD) δ 7.86 – 7.80 (m, 2H), 7.42 (dd, *J* = 8.5, 1.6 Hz, 1H), 7.33 – 7.18 (m, 5H), 4.81 (dd, *J* = 7.6, 5.5 Hz, 1H), 4.47 – 4.32 (m, 1H), 3.58 (dd, *J* = 15.2, 5.5 Hz, 1H), 3.42 (dd, *J* = 15.2, 7.6 Hz, 1H), 3.18 (dd, *J* = 13.0, 3.6 Hz, 1H), 3.10-3.04 (m, 2H), 2.93 – 2.84 (m, 2H), 2.24 (q, *J* = 7.6 Hz, 2H), 1.32 (d, *J* = 6.9 Hz, 6H), 1.08 (t, *J* = 7.6 Hz, 3H). ^{13}C NMR (101 MHz, MeOD) δ 175.96, 171.68, 167.04, 150.86, 146.74, 136.78, 135.18, 128.77, 128.31, 126.58, 125.27, 121.31, 118.64, 52.86, 49.38, 43.08, 37.43, 34.73, 34.07, 28.49, 23.14, 8.60. UPLC-MS (ESI-MS) *m/z*: calculated for $C_{25}H_{33}N_4O_2S^+$ 453.23, found 453.24 $[M+H]^+$.

Synthesis of compound 40.

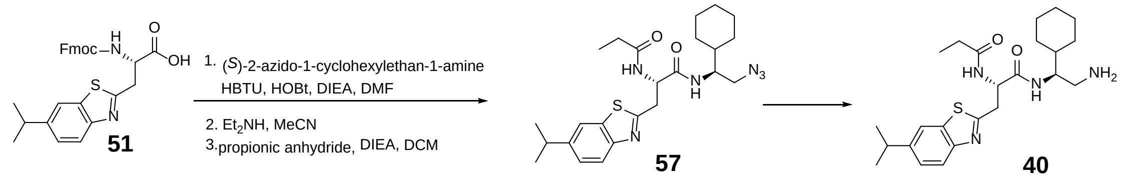

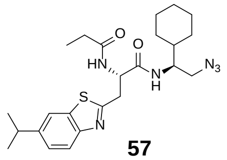

57

(S)-N-((S)-2-azido-1-cyclohexylethyl)-3-(6-isopropylbenzo[d]thiazol-2-yl)-2-propionamidopropanamide (57): Compound **57** was prepared from **51** in 65% yield over three steps by a similar procedure as that for compound **56**. ^{1}H NMR (400 MHz, $CDCl_3$) δ 7.84 (d, *J* = 8.4 Hz, 1H), 7.69 (d, *J* = 1.7 Hz, 1H), 7.50 (d, *J* = 9.1 Hz, 1H), 7.39 (d, *J* = 7.1 Hz, 1H), 7.34 (dd, *J* = 8.5, 1.7 Hz, 1H), 5.04 (td, *J* = 7.0, 4.8 Hz, 1H), 3.91 – 3.76 (m, 1H), 3.66 (dd, *J* = 15.9, 4.7 Hz, 1H), 3.43 (dd, *J* = 15.9, 7.0 Hz, 1H), 3.35 (dd, *J* = 5.0, 1.1 Hz, 2H), 3.04 (dt, *J* = 13.8, 6.9 Hz, 1H), 2.32 (q, *J* = 7.6 Hz, 2H), 1.70-1.60 (dd, *J* = 28.1, 15.3 Hz, 6H), 1.52 – 1.41 (m, 1H), 1.32 – 0.87 (m, 14H). ^{13}C NMR (101 MHz, $CDCl_3$) δ 174.11, 170.30, 167.09, 150.97, 146.46, 135.25, 125.39, 121.98, 118.79, 53.54, 52.31, 51.92, 38.95, 35.50, 34.24, 29.69, 29.65, 28.51, 26.09, 25.91, 25.85, 24.18, 9.68. UPLC-MS (ESI-MS) *m/z*: calculated for $C_{24}H_{35}N_6O_2S^+$ 471.25, found 471.27 $[M+H]^+$.

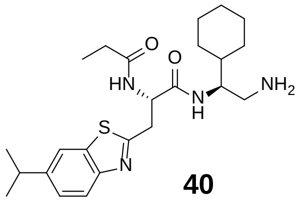

40

(S)-N-((S)-2-amino-1-cyclohexylethyl)-3-(6-isopropylbenzo[d]thiazol-2-yl)-2-propionamidopropanamide (40): Compound **40** was prepared from **57** in 88% yield by a similar procedure as that for compound **33.** ^{1}H NMR (400 MHz, MeOD) δ 7.99 (d, *J* = 8.9 Hz, 1H), 7.86-7.84 (m, 2H), 7.43 (d, *J* = 8.5 Hz, 1H), 4.87-4.84 (m, 1H), 3.94-3.90 (m, 1H), 3.68 (dd, *J* = 15.3, 5.8 Hz, 1H), 3.56 (dd, *J* = 15.2, 6.9 Hz, 1H), 3.29 – 3.20 (m, 1H), 3.12-2.91 (m, 2H), 2.31 (q, *J* = 7.6 Hz, 2H), 1.75-1.63 (m, 5H), 1.54 – 1.46 (m, 1H), 1.36 – 0.90 (m, 14H). ^{13}C NMR (101 MHz, MeOD) δ 176.08, 171.86, 167.03, 150.94, 146.76, 135.15, 125.30, 121.33, 118.65, 52.99, 52.48, 41.73, 39.59, 34.51, 34.08, 29.39, 28.57, 28.25, 25.70, 25.50, 25.43, 23.14, 8.70. UPLC-MS (ESI-MS) *m/z*: calculated for $C_{24}H_{37}N_4O_2S^+$ 445.26, found 445.27 $[M+H]^+$.

Synthesis of compound 41.

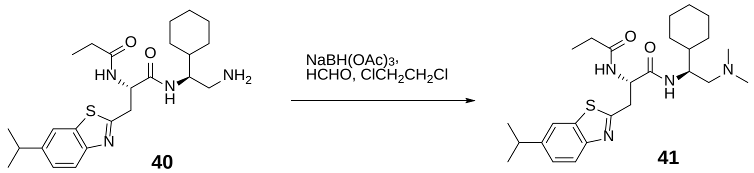

(S)-N-((S)-1-cyclohexyl-2-(dimethylamino)ethyl)-3-(6-isopropylbenzo[d]thiazol-2-yl)-2-propionamidopropanamide (41): Compound **40** (45 mg, 0.1 mmol) and formaldehyde solution 37 wt. % in H_2O (66 mg, 0.8 mmol) were mixed in 1,2-dichloroethane (5 mL) and then treated with sodium triacetoxyborohydride (86 mg, 0.4 mmol). The mixture was stirred at room temperature for 3 h until compound **40** was consumed. Then the reaction mixture was quenched by adding 1N NaOH, and concentrated. The residue was purified by HPLC to give compound **41** (40 mg, 83%). ^{1}H NMR (400 MHz, MeOD) δ 7.87 (d, *J* = 8.4 Hz, 1H), 7.83 (d, *J* = 0.5 Hz, 1H), 7.42 (d, *J* = 8.4 Hz, 1H), 4.92-4.90 (m, 1H), 4.01-3.96 (m, 1H), 3.67 (dd, *J* = 15.5, 6.1 Hz, 1H), 3.55 (dd, *J* = 15.4, 7.6 Hz, 1H), 3.36-3.33 (m, 1H), 3.11-3.02 (m, 2H), 2.93 (s, 6H), 2.32 (q, *J* = 7.6 Hz, 2H), 1.74 – 1.47 (m, 6H), 1.36 – 0.84 (m, 14H). ^{13}C NMR (101 MHz, MeOD) δ 176.63, 172.01, 166.81, 151.01, 146.72, 135.19, 125.24, 121.61, 118.62, 59.32, 53.34, 50.15, 40.15, 34.09, 34.06, 29.23, 28.55, 28.02, 25.63, 25.49, 25.42, 23.15, 23.14, 8.62. UPLC-MS (ESI-MS) *m/z*: calculated for $C_{26}H_{41}N_4O_2S^+$ 473.29, found 473.32 [M+H]$^+$.

Synthesis of compound 42.

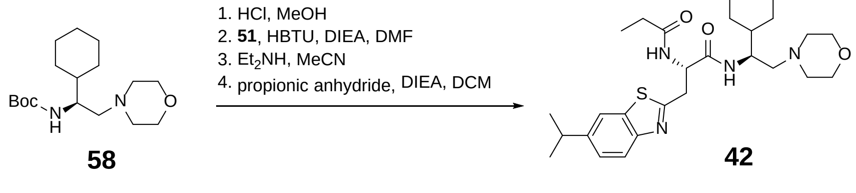

(S)-N-((S)-1-cyclohexyl-2-morpholinoethyl)-3-(6-isopropylbenzo[d]thiazol-2-yl)-2-propionamidopropanamide (42): The synthesis of tert-butyl (S)-(1-cyclohexyl-2-morpholinoethyl)carbamate (**58**) has been reported previously.[3] Hydrogen chloride solution (4.0 M in dioxane, 1 mL) was added to a solution of **58** (30 mg, 0.1 mmol, 1 equiv.) in MeOH (5 mL). The solution was stirred overnight and concentrated. The residue in DMF (2 mL) was added to a solution of **51** (47 mg, 0.1 mmol, 1 equiv.), HBTU (55 mg, 0.14 mmol, 1.5 equiv.) and DIEA (50 µL, 0.29mmol, 3 equiv.) in DMF (20 mL). The resultant mixture was stirred at room temperature for 1 h. The solution was diluted with EtOAc and washed with H_2O, saturated sodium bicarbonate, 1.0 M HCl, brine and dried over sodium sulfate. After removal of the solvent under vacuum, the residue was treated with 0.5 mL diethylamine in Acetonitrile (5 mL) for 1 h. The reaction mixture was evaporated and dissolved in DCM (5 mL). This solution was treated with propionic anhydride (25 mg, 0.2 mmoL, 2 equiv.) and DIEA (69 µL, 0.4 mmol, 4 equiv.). The resulting reaction mixture was stirred for half an hour and then was evaporated. The residue was purified by HPLC to afford compound **42** (31 mg, 63%). ^1H NMR (400 MHz, MeOD) δ 7.87 (d, *J* = 8.4 Hz, 1H), 7.82 (d, *J* = 1.2 Hz, 1H), 7.41 (dd, *J* = 8.4, 1.5 Hz, 1H), 4.78 (t, *J* = 6.9 Hz, 1H), 4.18 – 3.71 (m, 6H), 3.68-3.63 (m, 2H), 3.56 (dd, *J* = 15.4, 7.0 Hz, 1H), 3.41-3.33 (m, 2H), 3.25 – 3.01 (m, 3H), 2.35 (q, *J* = 7.6 Hz, 2H), 1.70 – 1.45 (m, 6H), 1.35 – 0.87 (m, 14H). ^{13}C NMR (101 MHz, MeOD) δ 176.80, 171.98, 166.40, 151.02, 146.77, 135.22, 125.26, 121.71, 118.63, 63.35, 59.00, 53.99, 49.33, 39.92, 34.10, 33.98, 29.21, 28.46, 28.12, 25.59, 25.46, 25.39, 23.16, 8.62. UPLC-MS (ESI-MS) *m/z*: calculated for $C_{28}H_{43}N_4O_3S^+$ 515.31, found 515.26 $[M+H]^+$.

Synthesis of compounds 43 and 44.

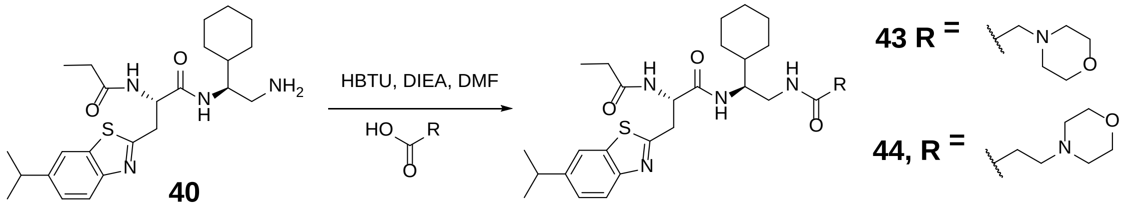

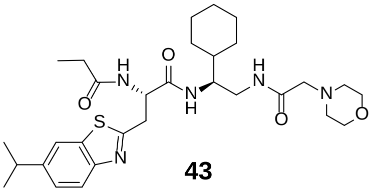
43

(S)-N-((S)-1-cyclohexyl-2-(2-morpholinoacetamido)ethyl)-3-(6-isopropylbenzo[d]thiazol-2-yl)-2-propionamidopropanamide (43): Compound **40** (40 mg, 0.09 mmol, 1 equiv.) was added to a solution of 2-morpholinoacetic acid (16 mg, 0.11 mmol, 1.2 equiv.), HBTU (51 mg, 0.13 mmol, 1.5 equiv.) and DIEA (47 µL, 0.27 mmol, 3 equiv.) in DCM (5 mL). The resultant mixture was stirred at room temperature for 1 h and concentrated. The residue was purified by HPLC to afford compound **43** (45 mg, 87%). ^{1}H NMR (400 MHz, MeOD) δ 7.88 (d, *J* = 8.4 Hz, 1H), 7.84 (s, 1H), 7.44 (d, *J* = 8.5 Hz, 1H), 4.93-4.91 (s, 1H), 4.14 – 3.75 (m, 8H), 3.65 (dd, *J* = 15.5, 5.4 Hz, 1H), 3.58-3.46 (m, 4H), 3.17-3.04 (m, 3H), 2.35 (q, *J* = 7.5 Hz, 2H), 1.73-1.62 (m, 6H), 1.38 – 0.86 (m, 15H). ^{13}C NMR (101 MHz, MeOD) δ 176.25, 171.48, 167.27, 164.01, 150.97, 146.76, 135.17, 125.30, 121.58, 118.68, 63.38, 56.94, 53.98, 53.03, 52.57, 40.82, 39.59, 34.73, 34.09, 29.59, 28.74, 28.41, 25.85, 25.62, 25.54, 23.16, 23.14, 8.75. UPLC-MS (ESI-MS) *m/z*: calculated for $C_{30}H_{46}N_5O_4S^+$ 572.33, found 572.21[M+H]$^+$.

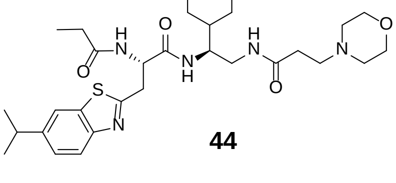
44

(S)-N-((S)-1-cyclohexyl-2-(3-morpholinopropanamido)ethyl)-3-(6-isopropylbenzo[d]thiazol-2-yl)-2-propionamidopropanamide (44): Compound **44** was prepared from **40** in 81% yield by a similar procedure as that for compound **43.** ^{1}H NMR (400 MHz, MeOD) δ 7.87 (d, *J* = 8.5 Hz, 1H), 7.83 (d, *J* = 1.6 Hz, 1H), 7.43 (dd, *J* = 8.5, 1.7 Hz, 1H), 4.86 (dd, *J* = 7.8, 5.4 Hz, 1H), 4.07-4.04 (m, 2H), 3.88 – 3.76 (m, 3H), 3.64 (dd, *J* = 15.4, 5.3 Hz, 1H), 3.57 – 3.37 (m, 6H), 3.17-3.06 (m, 4H), 2.69 (t, *J* = 6.4 Hz, 2H), 2.34 (q, *J* = 7.5 Hz, 2H), 1.72-1.60 (m, 5H), 1.39 – 0.86 (m, 15H). ^{13}C NMR (101 MHz, MeOD) δ 176.00, 171.58, 170.51, 167.14, 150.98, 146.74, 135.17, 125.28, 121.57, 118.63, 63.61, 54.19, 53.25, 53.18, 51.89, 40.78, 39.66, 34.80, 34.09, 29.55, 28.72, 28.47, 28.43, 25.87, 25.66, 25.58, 23.16, 23.15, 8.73. UPLC-MS (ESI-MS) *m/z*: calculated for $C_{31}H_{48}N_5O_4S^+$ 586.34, found 586.29 [M+H]$^+$.

Synthesis of compounds 45.

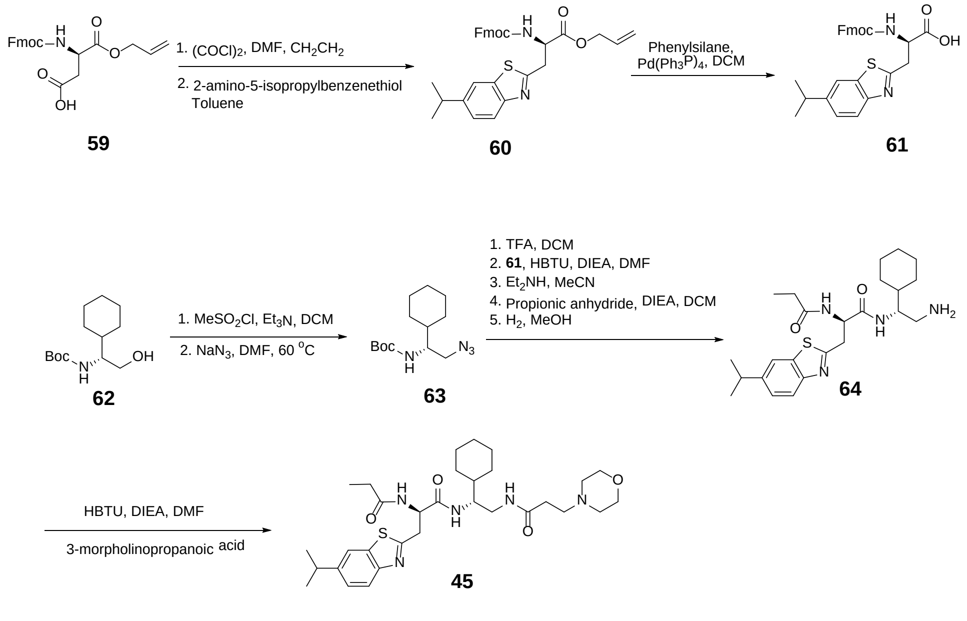

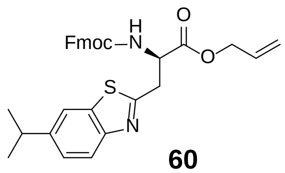

60

Allyl (R)-2-((((9H-fluoren-9-yl)methoxy)carbonyl)amino)-3-(6-isopropylbenzo[d]thiazol-2-yl)propanoate (60): Intermediate **60** was prepared from (R)-3-((((9H-fluoren-9-yl)methoxy)carbonyl)amino)-4-(allyloxy)-4-oxobutanoic acid (**59**) in 56% yield by a similar procedure as that for **49**. ^{1}H NMR (400 MHz, $CDCl_3$) δ 7.99 (d, *J* = 8.4 Hz, 1H), 7.79 (d, *J* = 7.5 Hz, 2H), 7.72 (s, 1H), 7.68 – 7.58 (m, 2H), 7.45-7.40 (m, 3H), 7.36 – 7.22 (m, 2H), 6.43 (d, *J* = 8.4 Hz, 1H), 5.98-5.89 (m, 1H), 5.37 (d, *J* = 17.2 Hz, 1H), 5.26 (dd, *J* = 10.4, 0.9 Hz, 1H), 5.08 – 4.94 (m, 1H), 4.73 (d, *J* = 4.5 Hz, 2H), 4.44 (d, *J* = 7.3 Hz, 2H), 4.27 (t, *J* = 7.2 Hz, 1H), 3.78 (d, *J* = 5.0 Hz, 2H), 3.08 (dt, *J* = 13.8, 6.9 Hz, 1H), 1.36 (d, *J* = 6.9 Hz, 6H). ^{13}C NMR (101 MHz, $CDCl_3$) δ 170.28, 166.10, 156.13, 150.40, 146.82, 143.90, 143.83, 141.33, 135.13, 131.46, 127.76, 127.14, 125.73, 125.27, 122.27, 120.03, 119.00, 118.80, 67.40, 66.53, 53.50, 47.14, 35.55, 34.30, 24.23. UPLC-MS (ESI-MS) *m/z*: calculated for $C_{31}H_{31}N_2O_4S^+$ 527.20, found 527.15 $[M+H]^+$.

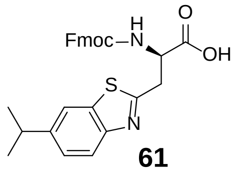

61

(R)-2-((((9H-fluoren-9-yl)methoxy)carbonyl)amino)-3-(6-isopropylbenzo[d]thiazol-2-yl)propanoic acid (61): Intermediate **61** was prepared from **60** in 84% yield by a similar procedure as that for **51**. ^{1}H NMR (400 MHz, DMSO) δ 8.08 – 7.79 (m, 5H), 7.65 (dd, *J* = 11.4, 7.7 Hz, 2H), 7.41-7.36 (m, 3H), 7.27 (t, *J* = 7.4 Hz, 1H), 7.20 (t, *J* = 7.4 Hz, 1H), 4.56 (td, *J* = 9.1, 4.6 Hz, 1H), 4.28-4.18 (m, 3H), 3.61 (dd, *J* = 15.0, 4.2 Hz, 1H), 3.45 (dd, *J* = 14.9, 10.0 Hz, 1H), 3.02 (dt, *J* = 13.6, 6.8 Hz, 1H), 1.25 (d, *J* = 6.7 Hz, 6H). ^{13}C NMR (101 MHz, DMSO) δ 172.72, 167.26, 156.41, 151.47, 146.12, 144.23, 144.14, 141.16, 135.67, 128.06, 127.49, 125.71,

125.66, 125.50, 122.46, 120.55, 119.47, 66.19, 54.11, 47.05, 35.55, 33.98, 24.54. UPLC-MS (ESI-MS) *m/z*: calculated for $C_{28}H_{27}N_2O_4S^+$ 487.17, found 487.18 $[M+H]^+$.

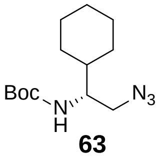

63

tert-butyl (R)-(2-azido-1-cyclohexylethyl)carbamate (63): Compound **63** was made by utilizing a reported method.[2] MsCl (710 mg, 6.2 mmol, 1.5 equiv.) was added dropwise to a solution of N-Boc-D-cyclohexylglycinol (1.00 g, 4.1 mmol, 1 equiv.) and Et_3N (1.7 mL, 12.3 mmol, 3 equiv.) in CH_2Cl_2 (20 mL) at 0 °C. The mixture was stirred 3 h at 0 °C and diluted with CH_2Cl_2. The mixture was washed with sat. aq $NaHCO_3$ (2 x 20 mL), 1M HCl, and brine. The organic layer was dried (Na_2SO_4) and the solvent was removed *in vacuo*. The residue was dissolved in DMF and NaN_3 (802 mg, 12.3 mmol, 3 equiv.) was added. This reaction mixture was stirred at 60°C overnight and cooled to room temperature. EtOAc and H_2O were added to this mixture and the aqueous layer was extracted with EtOAc. The organic layer was washed with H_2O and brine. The organic layer was dried (Na_2SO_4) and the solvent was removed under vacuum. The crude product was purified by flash chromatography to produce compound **63** (617 mg, 56% over two steps). ^{1}H NMR (400 MHz, $CDCl_3$) δ 4.60 (d, *J* = 8.6 Hz, 1H), 3.63 – 3.29 (m, 3H), 1.79-1.65 (m, 5H), 1.54 – 1.36 (m, 11H), 1.33 – 0.86 (m, 6H). ^{13}C NMR (101 MHz, $CDCl_3$) δ 155.57, 79.48, 54.82, 52.72, 39.31, 29.77, 28.86, 28.36, 28.29, 26.16, 25.97, 25.96. UPLC-MS (ESI-MS) *m/z*: calculated for $C_{13}H_{25}N_4O_2^+$ 269.20, found $[M+H]^+$.

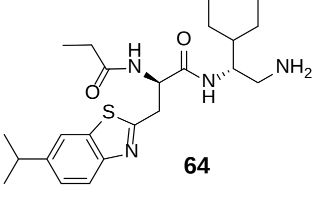

64

(R)-N-((R)-2-amino-1-cyclohexylethyl)-3-(6-isopropylbenzo[d]thiazol-2-yl)-2-propionamidopropanamide (64): Compound **64** was prepared from **61** and **62** in 51% yield over 5 steps by a similar procedure as that for compound **40.** ^{1}H NMR (400 MHz, MeOD) δ 7.97 (d, *J* = 9.0 Hz, 1H), 7.88 – 7.81 (m, 2H), 7.43 (dd, *J* = 8.6, 1.5 Hz, 1H), 4.91 (m, 1H), 3.97 – 3.87 (m, 1H), 3.68 (dd, *J* = 15.3, 5.9 Hz, 1H), 3.56 (dd, *J* = 15.2, 6.9 Hz, 1H), 3.25 (dd, *J* = 12.9, 3.0 Hz, 1H), 3.08 (dt, *J* = 13.8, 6.9 Hz, 1H), 3.01 – 2.93 (m, 1H), 2.31 (q, *J* = 7.6 Hz, 2H), 1.76 – 1.61 (m, 5H), 1.55 – 1.47 (m, 1H), 1.34 – 0.90 (m, 14H). ^{13}C NMR (101 MHz, MeOD) δ 176.09, 171.87, 167.03, 150.95, 146.77, 135.15, 125.30, 121.31, 118.66, 52.97, 52.48, 41.77, 39.59, 34.49, 34.08, 29.40, 28.56, 28.24, 25.70, 25.49, 25.43, 23.14, 8.70. UPLC-MS (ESI-MS) *m/z*: calculated for $C_{24}H_{37}N_4O_2S^+$ 445.26, found 445.41 $[M+H]^+$.

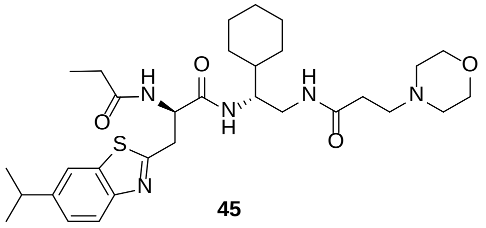

45

(R)-N-((R)-1-cyclohexyl-2-(3-morpholinopropanamido)ethyl)-3-(6-isopropylbenzo[d]thiazol-2-yl)-2-propionamidopropanamide (45): Compound **45** was prepared from **64** in 83% yield by a similar procedure as that for compound **44**. ^{1}H NMR (400 MHz, MeOD) δ 7.87 (d, *J* = 8.5 Hz, 1H), 7.84 (d, *J* = 1.6 Hz, 1H), 7.43 (dd, *J* = 8.5, 1.6 Hz, 1H), 4.87 – 4.82 (m, 1H), 4.08-4.05 (m, 2H), 3.87 – 3.76 (m, 3H), 3.64 (dd, *J* = 15.4, 5.4 Hz, 1H), 3.56 – 3.37 (m, 6H), 3.20 – 3.03 (m, 4H), 2.69 (t, *J* = 6.3 Hz, 2H), 2.34 (q, *J* = 7.6 Hz, 2H), 1.72-1.60 (m, 5H), 1.38 – 0.87 (m, 16H). ^{13}C NMR (101 MHz, MeOD) δ 176.01, 171.60, 170.52, 167.11, 151.01, 146.74, 135.17, 125.28, 121.57, 118.63, 63.61, 54.19, 53.25, 53.20, 51.89, 40.80,

39.67, 34.78, 34.09, 29.55, 28.72, 28.47, 28.38, 25.86, 25.65, 25.58, 23.15, 8.73. UPLC-MS (ESI-MS) *m/z*: calculated for $C_{31}H_{48}N_5O_4S^+$ 586.34, found 586.28 $[M+H]^+$.

Synthesis of compounds 46 and 47.

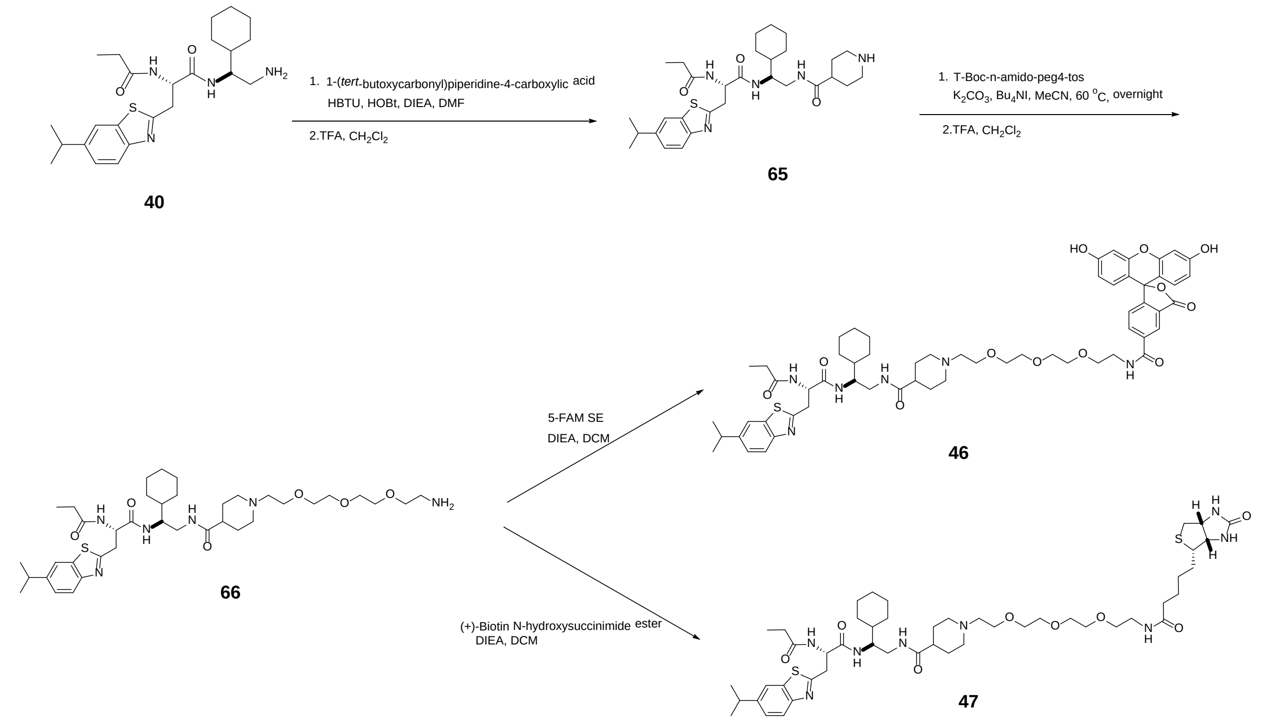

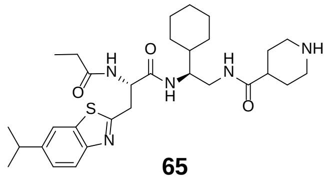

65

N-((S)-2-cyclohexyl-2-((S)-3-(6-isopropylbenzo[d]thiazol-2-yl)-2-propionamidopropanamido)ethyl)piperidine-4-carboxamide (65): Compound **40** (150 mg, 0.34 mmol, 1 equiv.) was added to a solution of 1-(tert-butoxycarbonyl)piperidine-4-carboxylic acid (93 mg, 0.40 mmol, 1.2 equiv.), HBTU (192 mg, 0.51 mmol, 1.5 equiv.) and DIEA (176 µL, 1.01 mmol, 3 equiv.) in DCM (10 mL). The resultant mixture was stirred at room temperature for 1 h and concentrated. The residue was dissolve in EtOAc and washed with H_2O, saturated

sodium bicarbonate, 1.0 M HCl, brine and dried over sodium sulfate. After removal of the solvent under vacuum, the residue was treated with TFA (2 ml) in DCM (10 mL) and stirred for 5 h. This reaction mixture was concentrated and purified by HPLC to afford compound **65** (139 mg, 74%). ^{1}H NMR (400 MHz, MeOD) δ 7.87 (d, *J* = 8.5 Hz, 1H), 7.83 (s, 1H), 7.42 (dd, *J* = 8.5, 1.5 Hz, 1H), 4.89 (dd, *J* = 7.8, 5.4 Hz, 1H), 3.80 (ddd, *J* = 10.2, 7.0, 3.5 Hz, 1H), 3.63 (dd, *J* = 15.4, 5.5 Hz, 1H), 3.53 – 3.36 (m, 4H), 3.12-3.04 (m, 2H), 2.98-2.90 (m, 2H), 2.49 – 2.38 (m, 1H), 2.32 (q, *J* = 7.6 Hz, 2H), 1.98 – 1.81 (m, 4H), 1.72-1.62 (m, 5H), 1.39 – 0.92 (m, 15H). ^{13}C NMR (101 MHz, MeOD) δ 175.87, 174.55, 171.35, 167.22, 150.93, 146.73, 135.21, 125.25, 121.57, 118.63, 54.31, 53.02, 42.89, 40.66, 39.79, 39.39, 34.95, 34.08, 29.54, 28.66, 28.32, 25.89, 25.72, 25.66, 25.14, 25.08, 23.16, 8.70. UPLC-MS (ESI-MS) *m/z*: calculated for $C_{30}H_{46}N_5O_3S^+$ 556.33, found 556.22 [M+H] $^+$.

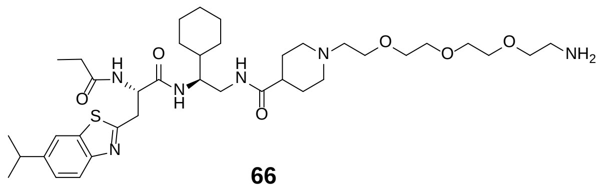

66

1-(2-(2-(2-(2-aminoethoxy)ethoxy)ethoxy)ethyl)-N-((S)-2-cyclohexyl-2-((S)-3-(6-isopropylbenzo[d]thiazol-2-yl)-2-propionamidopropanamido)ethyl)piperidine-4-carboxamide (66): A suspension of compound **65** (120 mg, 0.22 mmol, 1 equiv.), T-Boc-n-amido-peg4-tos (126 mg, 0.28 mmol, 1.3 equiv.), K_2CO_3 (89 mg, 0.65 mmol, 3 equiv.) and Tetrabutylammonium iodide (8.0 mg, 0.02 mmol, 0.1 equiv.) in DMF (10 mL) was stirred at 60°C overnight. The reaction was cooled and diluted with EtOAc and H_2O. The organic layer was washed with saturated sodium bicarbonate, 1.0 M HCl, brine and dried over sodium sulfate. After removal of the solvent under vacuum, the residue was treated with TFA (2 ml) in DCM (10

mL) and stirred for 5 h at room temperature. This reaction mixture was concentrated and purified by HPLC to afford compound **66** (103 mg, 65%). ^{1}H NMR (400 MHz, MeOD) δ 7.87 (d, *J* = 8.4 Hz, 1H), 7.83 (s, 1H), 7.42 (dd, *J* = 8.5, 1.4 Hz, 1H), 4.86 (dd, *J* = 8.9, 3.5 Hz, 1H), 3.85 – 3.60 (m, 16H), 3.57 – 3.36 (m, 4H), 3.28-3.23 (m, 2H), 3.17-3.05 (m, 4H), 2.53 – 2.40 (m, 1H), 2.32 (q, *J* = 7.6 Hz, 2H), 2.08 – 1.86 (m, 4H), 1.78 – 1.56 (m, 5H), 1.50 – 0.86 (m, 15H). HRMS (ESI-MS) *m/z*: calculated for $C_{38}H_{63}N_6O_6S^+$ 731.4524, found 731.4515 $[M+H]^+$.

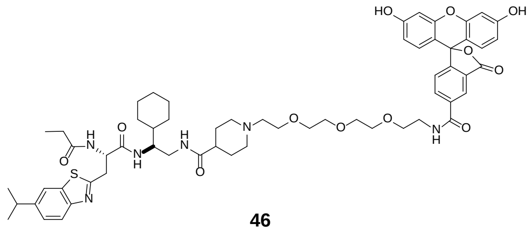

46

N-((S)-2-cyclohexyl-2-((S)-3-(6-isopropylbenzo[d]thiazol-2-yl)-2-propionamidopropanamido)ethyl)-1-(1-(3',6'-dihydroxy-3-oxo-3H-spiro[isobenzofuran-1,9'-xanthen]-5-yl)-1-oxo-5,8,11-trioxa-2-azatridecan-13-yl)piperidine-4-carboxamide (46): To a solution of **66** (30 mg, 0.04 mmol, 1 equiv.) and 5-Carboxyfluorescein N-hydroxysuccinimide ester (29 mg, 0.06 mmol, 1.5 equiv.) in DCM (5 mL) was added DIEA (21 µL, 0.12 mmol, 3 equiv.). The reaction was stirred at room temperature overnight and concentrated. The residue was purified by HPLC to get compound **46** (22 mg, 49%). ^{1}H NMR (400 MHz, MeOD) δ 8.49 (s, 1H), 8.23 (dd, *J* = 8.0, 1.4 Hz, 1H), 7.84 (d, *J* = 8.4 Hz, 1H), 7.80 (s, 1H), 7.40 (dd, *J* = 8.5, 1.4 Hz, 1H), 7.34 (d, *J* = 8.0 Hz, 1H), 6.78 (d, *J* = 2.2 Hz, 2H), 6.68 (d, *J* = 8.7 Hz, 2H), 6.61 (dd, *J* = 8.7, 2.1 Hz, 2H), 4.85-4.83 (m, 1H), 3.94 – 3.56 (m, 18H), 3.51-3.38 (m, 4H), 3.19 – 2.77 (m, 4H), 2.48-2.42 (m, 1H), 2.30 (q, *J* = 7.6 Hz, 2H), 2.13 – 1.87 (m, 4H), 1.68-1.59 (m, 5H), 1.40 – 0.88 (m, 15H). HRMS (ESI-MS) *m/z*: calculated for $C_{59}H_{73}N_6O_{12}S^+$1089.5002, found 1089.4994 $[M+H]^+$.

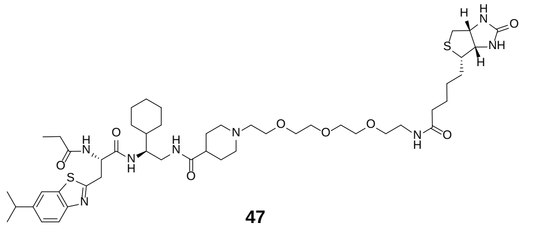

47

N-((S)-2-cyclohexyl-2-((S)-3-(6-isopropylbenzo[d]thiazol-2-yl)-2-propionamidopropanamido)ethyl)-1-(13-oxo-17-((3aS,4S,6aR)-2-oxohexahydro-1H-thieno[3,4-d]imidazol-4-yl)-3,6,9-trioxa-12-azaheptadecyl)piperidine-4-carboxamide (47): To a solution of **66** (30 mg, 0.04 mmol, 1 equiv.) and (+)-Biotin N-hydroxysuccinimide ester (21 mg, 0.06 mmol, 1.5 equiv.) in DCM (5 mL) was added DIEA (21 µL, 0.12 mmol, 3 equiv.). The reaction was stirred at room temperature overnight and concentrated. The residue was purified by HPLC to get compound **47** (25 mg, 64%). ^{1}H NMR (400 MHz, MeOD) δ 7.88 (d, *J* = 8.4 Hz, 1H), 7.84 (s, 1H), 7.67 (d, *J* = 9.5 Hz, 1H), 7.43 (d, *J* = 8.4 Hz, 1H), 5.18-5.11 (m, 1H), 4.51 (dd, *J* = 7.8, 5.0 Hz, 1H), 4.31 (dd, *J* = 7.8, 4.5 Hz, 1H), 3.87 – 3.79 (m, 3H), 3.71 – 3.59 (m, 10H), 3.56-3.36 (m, 10H), 3.26 – 3.18 (m, 1H), 3.13-3.04 (m, 2H), 3.00 – 2.89 (m, 2H), 2.72 (d, *J* = 12.8 Hz, 1H), 2.48-2.41 (m, 1H), 2.32 (q, *J* = 7.6 Hz, 2H), 2.23 (t, *J* = 7.3 Hz, 2H), 2.07-1.93 (m, 4H), 1.78 – 1.55 (m, 9H), 1.48 – 0.90 (m, 17H). HRMS (ESI-MS) *m/z*: calculated for $C_{48}H_{77}N_8O_8S_2^+$ 957.5300, found 957.5298 [M+H]$^+$.

Supplementary References

1. Rudrawar, S., Kondaskar, A. & Chakraborti, A.K. An efficient acid- and metal-free one-pot synthesis of benzothiazoles from carboxylic acids. *Synthesis-Stuttgart*, 2521-2526 (2005).
2. Wirtz, L., Auerbach, D., Jung, G. & Kazmaier, U. Fluorescence Labeling of Amino Acids and Peptides with 7-Aminocoumarins. *Synthesis-Stuttgart* **44**, 2005-2012 (2012).
3. Wang, S. & Seto, C.T. Enantioselective addition of vinylzinc reagents to 3,4-dihydroisoquinoline N-oxide. *Org Lett* **8**, 3979-3982 (2006).
